# Determination of inter-individual differences in humeral head centre translations in subjects with healthy shoulders

**DOI:** 10.1098/rsos.252309

**Published:** 2026-10-07

**Authors:** Eva C. Herbst, Heath B. Henninger, Flavia Stettler, Maximilian Modelhart, Matteo Tanadini, Stephen J. Ferguson, Philipp Moroder

**Affiliations:** ^1^ Laboratory for Orthopaedic Technology, Institute for Biomechanics, Department of Health Sciences and Technology, ETH Zurich, Zürich, Switzerland; ^2^ Schulthess Klinik, Zürich, Switzerland; ^3^ Department of Orthopaedics, The University of Utah, Salt Lake City, UT, USA; ^4^ Paracelsus Medical University, Salzburg, Austria; ^5^ Zurich Data Scientists GmbH, Zürich, Switzerland

**Keywords:** glenohumeral translations, shoulder, functional morphology, joint kinematics, biomechanics, biplanar fluoroscopy

## Abstract

The aims of this study were to (i) quantify glenohumeral (GH) joint translations in healthy shoulders, (ii) assess subject-specific variability in GH translations, (iii) determine whether bony joint geometry (glenoid height, width, retroversion, inclination, GH asphericity and congruence), upright scapular posture, motion and handheld load affect translation magnitude and (iv) analyse the position of the humeral head centre (HHC) starting point relative to different glenoid centre definitions. We measured translations of the HHC relative to the glenoid axes in 19 healthy subjects during five arm motions. Mean 3D, superoinferior (SI), anteroposterior (AP), and mediolateral (ML) translation magnitudes were 14.7% of glenoid height, 9.5% of glenoid height, 15.4% of glenoid width, and 7.4% of glenoid height, respectively. Glenoid height affected 3D and SI translation magnitude inclination angle, glenoid asphericity and scapular tilt affected 3D and AP translation magnitudes and motion affected 3D, AP and SI translation magnitudes (*p*≤ 0.05, but effect sizes are small and large confidence intervals warrant further experiments). The starting point of the HHC was closer to the glenoid geometric mean than to the inferior glenoid circle center, which may be useful clinically when attempting to restore healthy HHC positioning.

## 1. Introduction

Unlike many other joints in the human body, the glenohumeral (GH) joint has a large amount of mobility and relatively low passive stability at the mid range of motion [1]. Only a small region of the humeral articular surface is enclosed by the glenoid [2]. At the mid-ranges of motion, stability is thought to be mainly achieved through muscular forces that compress the humeral head into the glenoid, rather than through ligamentous constraints [1,3,4]. In functional shoulder instability cases, abnormal neuromuscular control causes humeral subluxation, a phenomenon which can be mitigated with functional electrostimulation that restores muscular activity [5,6]. These studies highlight the role of muscle activity in stabilizing the joint.

Nonetheless, even healthy/asymptomatic shoulders exhibit translations of a few millimeters [7–17]. These translations are probably enabled by the lack of GH congruence (i.e. a mismatch between the glenoid and humeral head radii of curvature), causing the humerus to translate before being restricted by the glenoid rim. This mismatch is most apparent in the bony glenoid and humeral head radii [2,18], and although cartilage reduces this mismatch [18,19], other studies report that a mismatch of 2–3 mm persists in the articular surfaces [20]. In addition to incongruence of the articular surfaces, the low enclosure of the glenoid may allow the humeral head to approach the glenoid rim, initiating rolling about the rim contact point, which could also result in translations of the humeral head centre. In healthy subjects, muscles would be recruited to prevent excessive translations that could result in subluxation.

GH translations vary between subjects. Inter-subject variation (standard deviation (s.d.) of translations) exceeded intra-subject variation between biceps tenodesis and contralateral healthy shoulders [10], but the cause of this variation remains unknown. (s.d. in GH translations is typically around 1–2 mm in superoinferior (SI), anteroposterior (AP) and mediolateral (ML) directions, but can be up to 4.2 mm in the AP direction [7,9,10,14–16,21,22].) However, reporting means and s.d. does not reveal whether different subjects have different baselines (i.e. a different starting point of humerus relative to glenoid, but with the same humeral translation trajectories across elevation angles) or whether the translation patterns themselves vary between subjects. Few studies account for different subject baselines [7] and subject-specific starting points and patterns [15].

A comprehensive analysis of subject-specific variability in GH translations and potential underlying causes for such variability is warranted. Translational variability may result from various factors, including joint geometry. Variation in GH congruence exists between subjects and affects translation magnitude during actively positioned cadaver experiments, although sample sizes of these studies remain small [4,19]. More downward glenoid inclination may reduce superior translations, but other studies show no effect [23–26]. Discrepancies in results might be due to variation in experimental protocols and populations studied. Retroversion has also been shown to affect GH translations in cadaveric studies, with increased retroversion increasing posterior translation [27]. Future work is needed to explore the effect of inclination angle, as well as glenoid retroversion angle, on GH translations.

The type of motion performed may also affect GH translations. GH excursion measured with biplanar fluoroscopy differed between coronal abduction, flexion and scapular plane abduction, but this difference could also be due to inter-subject variation, since the abduction group was made up of different subjects [9]. Therefore, further studies are needed to investigate the effect of motion while controlling for subject variability. External load could also affect GH translation. Some studies report that external loads affect scapular kinematics and scapulo-humeral rhythm (SHR) [28,29], which could, in turn, produce changes in GH kinematics, although in a uniplanar fluoroscopy study of SI translations, load only changed GH translations in rotator cuff tendinopathy subjects but not in controls [17].

The effect of scapular posture on GH translations also remains unexplored, but supine scapulothoracic orientation has been shown to correlate with static posterior humeral head decentring, and scapular posture impacts impingement simulations and correlates with clinical outcomes in reverse total shoulder arthroplasty [30–35]. Considering that a different scapular orientation in global space changes the GH joint pose for the same hand position in global space and the gravity vector relative to the glenoid surface, the effect of upright scapula posture with respect to a global coordinate system (i.e. in reference to the gravity vector) on GH translations should be investigated.

Subjects in existing studies are predominantly male; more equal representation is needed when characterizing healthy translations in the population, especially given that scapular size and shape differ significantly between males and females [36]. Furthermore, most studies report translations in absolute metrics (mm), but glenoid height has been reported to vary by several millimetres [2] and glenoid area varies with age [37]. Therefore, GH translation analyses should control for glenoid size differences when analysing the effects of other anatomical factors and operative interventions.

Given that previous studies are limited by lack of subject-specific analysis, small sample sizes, unequal sex distribution, isolated investigation of single anatomical factors and have not investigated posture or glenoid size effects, further work is warranted to evaluate several possible factors simultaneously to determine which anatomical factors affect GH translations, and would, therefore, be the most sensitive to target clinically. The aims of this study were to (i) analyse the *in vivo* translational kinematics of the humerus relative to the glenoid coordinate system in the healthy GH joint, (ii) assess subject variability in GH translation magnitude and pattern, (iii) determine whether differences in joint geometry, posture, load, and motion account for variability in translation magnitude and (iv) analyse the position of the humeral head starting point relative to different glenoid centre (GC) definitions. We hypothesized that subjects would exhibit variation in translation magnitudes and patterns and that translation magnitude correlates positively with more superior glenoid inclination, more retroversion, increased asphericity of the glenoid and humeral head, decreased joint congruence (ratio of humeral to glenoid radius) and increased glenoid size, and that, based on previous studies, handheld load would not affect translations. We also hypothesized that posture would influence translation patterns, e.g. a more posterior translation trajectory in subjects with more anterior scapular tilt. Regarding the humeral head starting point, this analysis did not have an *a priori* hypothesis but was conducted to provide healthy reference values that can serve as a baseline for surgical procedures aiming to restore humeral head centring.

Comprehensive assessment of GH translations and subject-specific variability contributes to better understanding of GH biomechanics, specifically how form relates to kinematics in the GH joint, can serve as a healthy baseline target for implant designs, and is important for computational biomechanical model input and validation. If strong correlations exist between anatomic parameters and *in vivo* translation measurements, such factors may be used to predict translations in new subjects, and may be factored into treatment planning on a subject-specific basis.

## 2. Materials and methods

### 2.1. Dataset

This study used an open-source biplanar fluoroscopy dataset of 20 healthy participants performing five different motions: coronal abduction (CA), scapular abduction (SA), forward elevation (FE), low internal/external rotation with the arm hanging at the side (ERaR) and high internal/external rotation (ERa90, arm at roughly 90^∘^ of humerothoracic coronal plane elevation and elbow flexed at 90^∘^) [38–41]. CA, SA and FE were also performed with a 2.2 kg handheld load (WCA, WSA and WFE, respectively). Healthy shoulders were defined as those without history of shoulder surgery or pain, and were screened for pathologies using radiographic imaging (see [39] for details). The data were recorded at 100 Hz, CT models were created with a slice thickness of 1.0 mm and models were registered to the biplane images using semi-automatic registration [39–41]. All subjects analysed had data collected for the right shoulder.

Subject O45_003_F_51_R (subject naming conventions based on [38]) was excluded, due to failure to establish the thumbs up position consistent with the other subjects [40,41], leaving 19 subjects in the analysis (ages 22–66 years, mean age 41.5 years, 9 female and 10 male). The ERaR trial of U35_001_F_26 was missing in the dataset and was, therefore, excluded, and the FE trial of U35_010_M_27 was excluded, due to a recording gap [40,41]. For all mesh analyses, scapula and humerus meshes were upsampled to 30,000 vertices using the 3D Slicer [42] Surface Toolbox to ensure even sampling and higher vertex density for calculations of anatomical parameters.

### 2.2. Quantification of translations

GH translations were quantified as movements of the humeral head centre (§2.2.1) along the glenoid axes (figure 1, §2.2.2). Using the glenoid coordinate system is a deviation from the International Society of Biomechanics (ISB) recommendation [43], but necessary to capture the translations along the joint surface [44], since glenoid axes do not necessarily align with scapular axes (figure 1), and glenoid inclination and retroversion vary among individuals. Scapula and humerus rigid body kinematics in the biplane coordinate system were calculated from biplanar bony landmarks [38] in Artisynth [45], and movement of the humeral head was then transformed into the glenoid coordinate system. For statistical analyses, translations were quantified as the translation magnitude (maximum humeral head excursion) across the entire motion trial. For three-dimensional (3D) translations, the humeral head translation magnitude was calculated as the maximum Euclidean distance in 3D. SI, AP and ML translation magnitudes were quantified as humeral head centre translation magnitudes in the glenoid SI, AP and ML axes. Subject-specific translation patterns were plotted as the humeral head position in the glenoid SI and AP axes across time (i.e. including patterns and not just maximum excursion). Analyses included the complete motion trajectory (e.g. adduction to abduction to adduction and internal to external to internal rotation).

**Figure 1.**
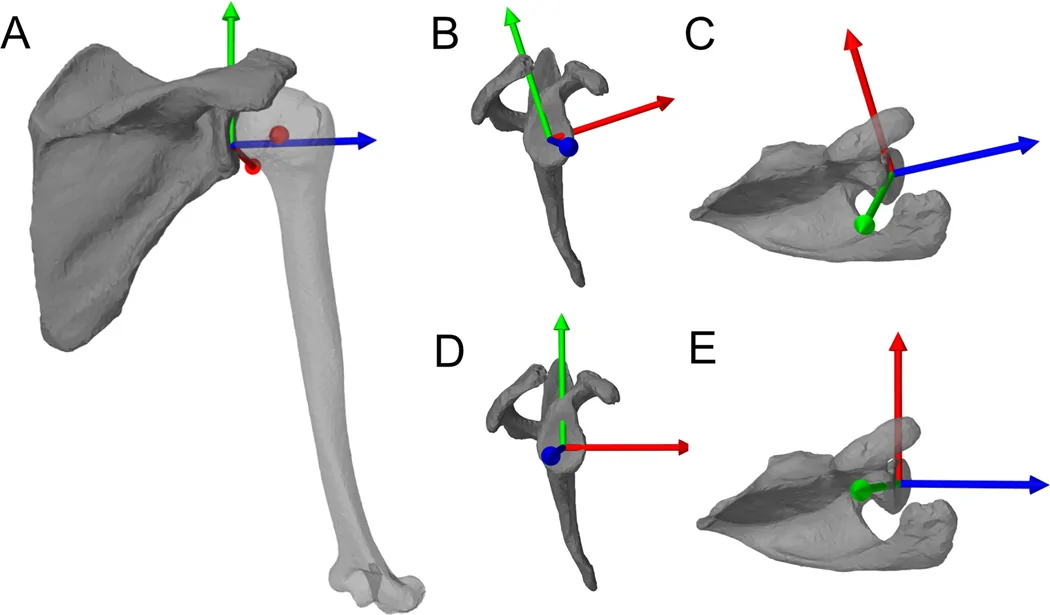
(A) Glenohumeral (GH) translations are quantified as excursions of the humeral head centre (red sphere) relative to the glenoid axes. The scapular axis-based, glenoid-centred coordinate system from the original dataset (B,C) was transformed to a glenoid axis-based coordinate system (D,E) for the analysis in this study. The X-axis (red) is the anteroposterior (AP) direction, the Y-axis (green) indicates the superoinferior (SI) direction and the Z-axis (blue) indicates the mediolateral (ML) direction.

#### 2.2.1. Humeral head centre

The perimeter of the humeral head along the anatomical neck was selected by a surgeon (M.M.) in 3D Slicer [42] to isolate the humeral head surface. A sphere was then fitted to this surface using the SciKit-Surgery sphere fitting algorithm [46], and the humeral head centre was defined as the centre of the sphere. The humeral head centre was recalculated (i.e. not the same as in [38]) to ensure methodological consistency with other studies in our group.

#### 2.2.2. Glenoid coordinate system

The perimeter of the subchondral glenoid was selected on the surface of the 3D scapular mesh by a surgeon (M.M.) in 3D Slicer [42]. The glenoid was then cut out using this perimeter, and singular value decomposition was performed to identify the main axes of the glenoid (numpy.linalg.svd v1.26.2, Python 3.11.9), with the Y-axis forming the first principal axis of the glenoid surface (glenoid SI direction), the X-axis forming the orthogonal axis (glenoid AP direction) and the Z-axis pointing away from the glenoid (glenoid ML direction). The GC was defined using an in-house algorithm to replicate, in 3D, the inferior glenoid ‘best-fit circle’ to maximize clinical compatibility of our measurements. Clinically, the GC is often defined as the center of this best-fit circle in two dimensions [47,48]. This GC measurement achieves better agreement when measured manually than defining the GC based on the intersect of the greatest glenoid longitudinal and transverse lines [48], and can be inferred from the glenoid bare spot in cases of severe bone erosion [49]. To determine the circle centre in 3D, the boundary of the glenoid was resampled to 49 points, and the most inferior point and the 13 adjacent points on each side of it were used to create an inferior glenoid curve, whose mean curvature was used to estimate the radius of the inferior best-fit circle. Each point on the inferior glenoid curve was projected along its normal by that radius, and the mean of the resulting points was then projected along the glenoid Z-axis to the glenoid surface using ray casting to define the glenoid inferior circle centre. Note that the GC was recalculated from the original dataset (i.e. not the same as in [38]) to ensure methodological consistency with other studies in our group.

#### 2.2.3. Starting position of humeral head

We also analysed whether the starting position of the humeral head was closer to the centre of the inferior glenoid best-fit circle (the centre of the glenoid coordinate system used in this study, which is often used clinically in two dimensions) or closer to the geometric mean of the glenoid. The starting position was calculated as the humeral head position in the first frame of the trial. The geometric mean was calculated as the mean position of all points on the glenoid perimeter curve loop that was used for calculating the inferior glenoid best-fit circle (see §2.2.2). Distances were calculated in the XY plane of the glenoid. The AP (X) position of the humeral head relative to the midline was also quantified. The end point (last frame) was also analysed to determine whether the analysis was sensitive to using the start or end point, both of which should represent a resting position (before and after performing the motion).

### 2.3. Quantification of anatomical parameters

#### 2.3.1. Glenoid height and width

Glenoid height and width were defined as the maximal dimensions along the glenoid Y and X axes, respectively (see §2.2.2), obtained by projecting all points into the relevant axis and calculating the maximum distance between the projected points.

#### 2.3.2. Asphericity and joint congruence

The glenoid and humeral head radii of curvature were determined by fitting spheres to the bony glenoid and humeral head (see §2.2.1) and calculating their radii. The joint congruence (match between the glenoid and humeral head radii) was defined as the ratio of humeral to glenoid radius. Asphericity of the glenoid and humeral head was defined as the mean absolute distance in millimetres between the surface mesh of the bony region and the sphere fitted to that mesh, i.e. deviation from a sphere (larger values indicate greater asphericity). The mean absolute distance was calculated as the mean of the absolute Hausdorff distances from each point on the bony mesh to the sphere using the ModelToModel Distance module in 3D Slicer (absolute_closest_point setting).

#### 2.3.3. Glenoid inclination angle and retroversion angle

Glenoid inclination and retroversion were calculated by implementing the 3D method from [50] in 3D Slicer. The scapular coronal plane was defined as the plane fitted to GC, trigonum spinae (TS) and the inferior angle (IA). The scapular axial plane was defined as the plane perpendicular to the scapular coronal plane and passing through TS and GC. Glenoid inclination was defined as the angle between the scapular axial plane, and the line connecting the GC and the centre of the glenoid sphere fit. Glenoid retroversion was defined as the angle between the scapular coronal plane and the line connecting the GC and the centre of the glenoid sphere fit. Negative retroversion angles indicate anteversion.

#### 2.3.4. Posture analysis

Scapular posture was defined using a subject-global coordinate system (see supplementary material, Appendix A, figure A1 D–F) with the SI axis defined by gravity and the ML and AP axes defined by the subject's anatomy [31], based on the right shoulder in an upright position using static trials [38]. All landmarks were used from the original dataset [38] with the exception of the GC, which was recalculated with our method (§2.2.2). The subject-global coordinate system originated on the incisura jugularis (IJ), and the Y-axis was aligned to the gravity vector (i.e. the global Z-axis from the Vicon calibration, which was oriented along the room's gravity axis), with positive Y being up. The AP axis (X-axis) was defined as the line from IJ to T5 (thoracic vertebra 5), projected into the plane orthogonal to Y, i.e. the transverse plane (positive X = anterior). The ML axis (Z-axis) was orthogonal to the Y and X axes (positive Z pointing to the right-hand side of the subject). The scapular Z-axis was defined as the line between the TS and GC. The scapular coronal plane was defined as the plane between the TS, GC and IA. Scapular upward rotation was defined as the angle between the global ML axis (Z-axis) and the local Z-axis of the scapula projected to the global YZ (coronal) plane (positive values indicate upward rotation of the lateral side of the scapula) (see supplementary material, Appendix A, figure A1 D). Scapular internal rotation was defined as the angle between the global ML axis and the local Z-axis of the scapula projected to the global XZ (transverse) plane (higher values indicating more internal rotation of the anterior scapula) (see supplementary material, Appendix A, figure A1 E). Scapular tilt was defined as the angle between the global positive anterior axis (X-axis) and the line orthogonal to the scapular coronal plane projected to the global XY (sagittal) plane (positive angles indicate anterior tilt of the superior scapula) (see supplementary material, Appendix A, figure A1 F). Note that these scapular orientation definitions differ from ISB definitions [43] to maximize clinical interpretability.

### 2.4. Statistical analysis

Statistical analyses were performed in R (R version 4.4.2, [51]) using RStudio [52] with a significance threshold of *p*≤ 0.05. To test if and how anatomical parameters and motions affect humeral head centre translation magnitudes, a linear mixed-effects model was used (lme4 package, [53]), with translation magnitude (log transformed) as the outcome variable. Separate models were created for 3D, AP, and SI translation magnitudes. Fixed factors (i.e. possible predictors of translations) were: motion, posture, glenoid height/width, glenoid inclination angle, retroversion angle, GH joint congruence (defined as the ratio between humeral head and glenoid radius of curvature), bony asphericity of the humerus, bony asphericity of the glenoid, scapular upward rotation, scapular internal rotation, scapular tilt, and whether an external load was applied.

Subjects were included as random factors to account for multiple motions tested per subject, and possible different baselines in translations between subjects. Absolute measures (glenoid height, glenoid width, glenoid and humeral head asphericity) were log transformed to normalize data. Variance inflation factor (VIF) analysis was used to assess multi-collinearity. Collinearity between glenoid width and glenoid height, and collinearity between scapular upward rotation and scapular tilt were observed (VIF > 5, Appendix B in the supplementary material). Glenoid height and width were positively correlated (linear regression coefficient of 0.81, i.e. 0.8 mm increase in width for every 1 mm increase in height, 95% CI [0.55, 1.08], *p* < 0.00001, Appendix B in the supplementary material). For 3D and SI translation models, glenoid height was used as a fixed factor to account for size differences in subjects and glenoid width was removed. For the AP translation model, glenoid width was used and height was removed. Scapular upward rotation and scapular tilt were negatively correlated (linear regression coefficient –0.58, 95% CI [–1.07, –0.10], *p* = 0.021). Scapular upward rotation was removed from the model, although it must be kept in mind that any significant effect of scapular tilt may be due to an effect of scapular upward rotation since the two are correlated. The final model was as follows:

$$\begin{array}{l} lmer(log(Translation)∼InclinationAngle+RetroversionAngle+MotionType+\\ Loaded(yes/no)+log(GlenoidAsphericity)+log(HumerusAsphericity)+\\ log(GlenoidHeight*)+JointCongruence+ScapIntRot+ScapTilt**+(1|Subject)), \end{array}$$

*^∗^ = width for AP model,^∗∗^ = correlated with scapular upward rotation.*

The assumptions (normality of residuals, linearity and homoscedasticity) of all three models were tested using QQ, Tukey–Anscombe, scale-location plots and factor-residual plots (Appendix B in the supplementary material). *P-*values from the linear mixed model were obtained via drop1 function (test = ‘Chi-square’). Pairwise post hoc comparisons between motions were performed using the general linear hypotheses (glht) function (multcomp package; [54]). The Tukey's HSD setting in glht was used to account for type-1 (false positive) errors due to multiple pairwise testing.

To determine whether the starting point (first frame) of the humeral head was closer to the centre of the inferior glenoid best-fit circle or the geometric mean of the glenoid, summary statistics were calculated on pooled data (all subjects and motions) to determine distances of the starting position to both reference points and to determine the number of trials in which the humeral head centre was closer to one of these reference points than the other. Per-motion analysis was also conducted to account for possible effects of motion type on starting-point position. The anteroposterior positioning of the humeral head in the starting point was also quantified relative to the glenoid midline. The same analysis was also replicated using the end point (last frame) instead of the starting point to determine whether this influenced the results, and start and end point positions in the glenoid XY plane were compared by determining the Euclidean distance between the two. Note all plots are deliberately scaled to glenoid height in both SI and AP directions to preserve the glenoid boundary shape i.e. to prevent distortion.

## 3. Results

Tests for the assumptions of the linear mixed-effects model were met (Appendix B in the supplementary material).

### 3.1. Glenohumeral translation magnitude

Subjects exhibited considerable variability in translation magnitude and range (table 1, figure 2 and supplementary material, Appendix A figures A11–A26).

**Figure 2.**
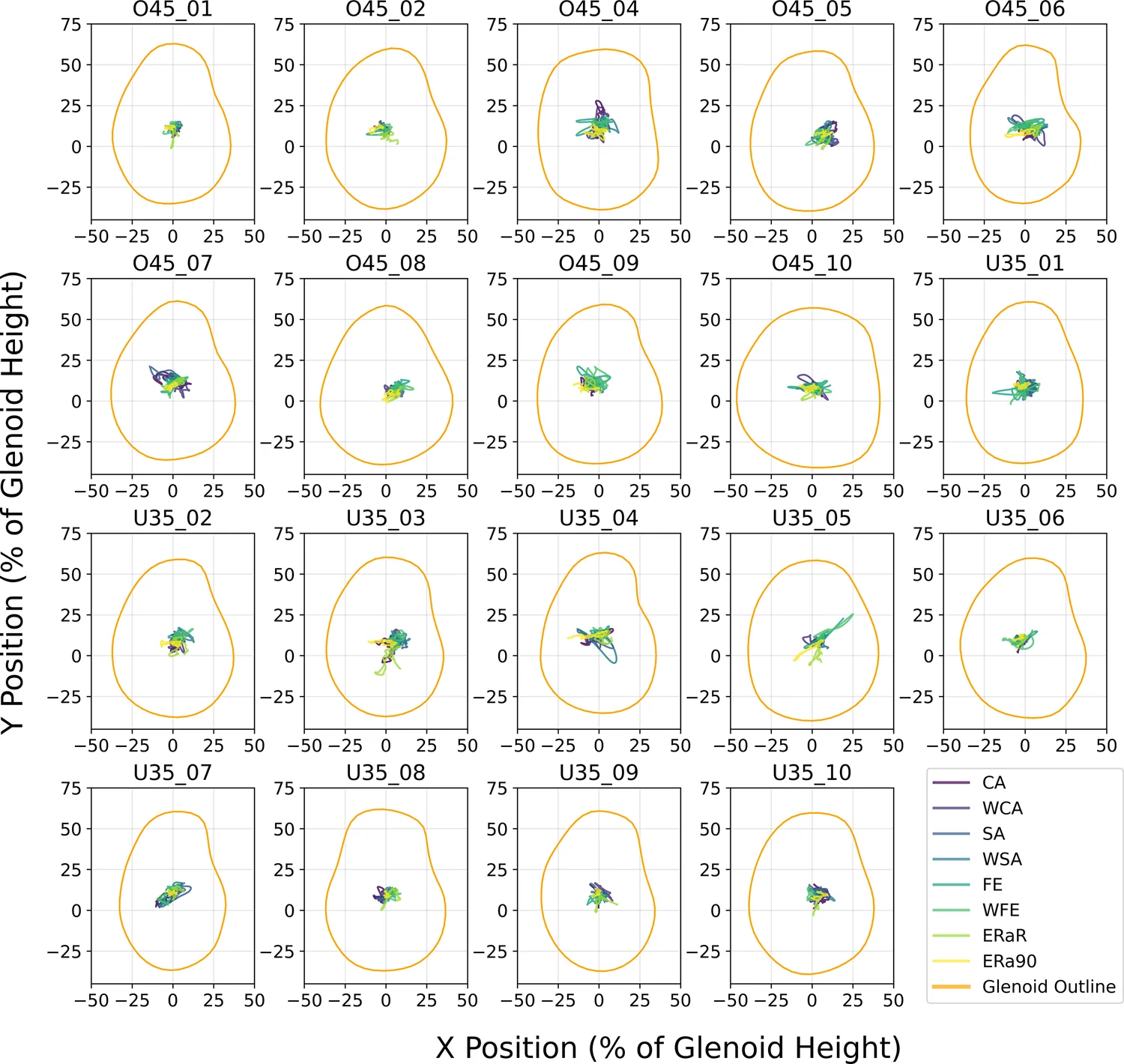
GH translations per subject, colour coded by motion. CA = coronal abduction, SA = scapular abduction, FE = forward elevation, ERaR = low internal/external rotation and ERa90 = high internal/external rotation. W indicates added 2.2kg load.

**Table 1.** Summary statistics of translations, bony anatomy and posture. *ML = mediolateral, AP = anteroposterior, SI = superoinferior*.

variable	mean ± s.d.	range
3D translations (mm)	4.9 ± 1.9	1.9–10.9
3D translations (% glenoid height)	14.7 ± 5.3	6.4–31.6
AP translations (mm)	3.9 ± 1.7	1.2–9.8
AP translations (% glenoid width)	15.4 ± 6.6	4.5–34.4
SI translations (mm)	3.1 ± 1.3	0.9–8.3
SI translations (% glenoid height)	9.5 ± 4.0	2.9–23.0
ML translations (mm)	2.5 ± 0.9	0.5–5.4
ML translations (% glenoid height)	7.4 ± 2.8	1.8–16.4
glenoid height (mm)	33.2 ± 3.5	26.6–39.6
glenoid width (mm)	25.6 ± 3.4	19.7–34.1
inclination angle (^∘^)	12.6 ± 3.8	5.0–19.1
retroversion angle (^∘^)	5.7 ± 4.6	–0.3–19.4
GH congruence	0.6 ± 0.1	0.5–0.7
glenoid asphericity (mm)	0.3 ± 0.1	0.2–0.4
humeral head asphericity (mm)	0.5 ± 0.1	0.3–0.6
scapular upward rotation (^∘^)	–14.3 ± 11.7	–36.4–6.2
scapular internal rotation (^∘^)	47.4 ± 7.2	27.9–60.5
scapular tilt (^∘^)	15.3 ± 10.5	–4.9–38.1

### 3.2. Subject-specific patterns

Individuals exhibited subject-specific translation patterns across similar motions, for example, when comparing SA and CA (figure 3), or FE with versus without handheld load (see supplementary material, Appendix A figure A27). The complete subject-specific translation plots per motion (see supplementary material, Appendix A figures A11-A26) also demonstrate these trends across other motions when comparing the conditions with and without handheld loads.

**Figure 3.**
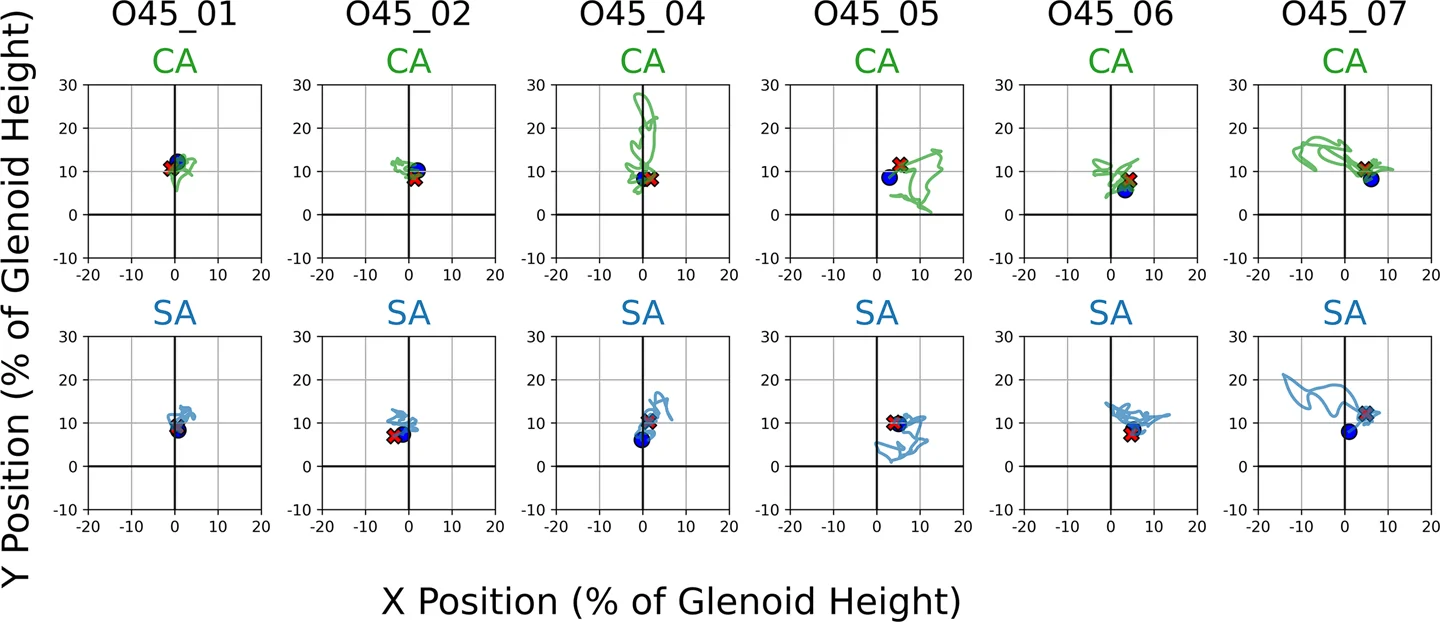
GH translations for coronal abduction (CA, green) and scapular abduction (SA, blue) motions, showing subject-specific trends across motion. Blue dot indicates starting position and red X indicates end position.

### 3.3. Glenoid size effect

Glenoid height significantly affected 3D and SI translation magnitudes (*p* = 0.05 and 0.01, table 2). Note that glenoid height was at the significance threshold for affecting 3D translation magnitude. A 1% increase in glenoid height was associated with a 0.78% increase in translation magnitude (but note large confidence intervals including zero, table 2). Glenoid height also affected SI translations with a 1.02% increase in SI translation magnitude for every 1% increase in glenoid height (note large confidence interval, not approaching zero). The possibility of a simple scaling relationship between glenoid height and 3D/SI translation magnitude was investigated further by creating a new linear mixed-effects model with translations (as a percentage of the glenoid height) as the outcome variable. In this new model, glenoid height was not significant, supporting the scaling relationship rather than a more complex effect of glenoid height on normalized translations (Appendix B in the supplementary material). There was no significant effect of glenoid width on AP translations.

**Table 2.** Bony joint geometry and scapular posture parameters with significant effects (*p*≤ 0.05) on translation magnitude. *AP = anteroposterior, ML = mediolateral, SI = superoinferior and CI = confidence interval.*

parameter	model	effect [95% CI] $e^{\beta }$ or $\beta^{*}$	% change in translation [95% CI]	p
glenoid height (mm)	3D	0.78 [0.00, 1.57]	0.78 [0.00, 1.57]% *for +1% increase in predictor*	0.05
glenoid height (mm)	SI	1.02 [0.27, 1.79]	1.02 [0.27, 1.79]% *for +1% increase in predictor*	0.01
glenoid asphericity^∗∗^ (mm)	3D	0.42 [0.01, 0.83]	0.42 [0.01, 0.83]% *for +1% increase in predictor*	0.04
glenoid asphericity^∗∗^ (mm)	AP	0.66 [0.12, 1.19]	0.66 [0.12, 1.19]% *for +1% increase in predictor*	0.02
inclination angle (^∘^)	3D	1.04 [1.01, 1.06]	3.75 [1.37, 6.20]% *for +1^∘^ increase in predictor*	<0.01
inclination angle (^∘^)	AP	1.05 [1.02, 1.08]	5.28 [2.24, 8.42]% *for +1^∘^ increase in predictor*	<0.01
scapular tilt^∗∗∗^ (^∘^)	3D	1.01 [1.00, 1.02]	1.01 [0.20, 1.83]% *for +1^∘^ increase in predictor*	0.02
scapular tilt^∗∗∗^(^∘^)	AP	1.01 [1.00, 1.02]	1.26 [0.19, 2.35]% *for +1^∘^ increase in predictor*	0.02

Note: ^∗^ The effect column shows $e^{\beta }$ for raw predictors (i.e. all angle measurements). Interpret $e^{\beta }$ as multiplicative factor on translation per +1 unit). Log transformed predictors (i.e. all mm measurements) show direct β and CI, and were not exponentiated since both outcome and predictor on log scale. Calculations for effect interpretations are reported in Appendix B in the supplementary material. ^∗∗^Mean distance from glenoid to fitted sphere. ^∗∗∗^ Scapular tilt correlated with upward rotation and the latter was removed from the model, therefore it must be kept in mind that scapular tilt effects might also be due to indirect effects of scapular upward rotation.

### 3.4. Bony joint geometry and posture

Inclination angle, glenoid asphericity and scapular tilt affected 3D and AP translation magnitudes (table 2), but confidence intervals of effect sizes are large. Note that because of collinearity between scapular tilt and scapular upward rotation, the effect of scapular tilt could also indirectly be due to changes in scapular upward rotation (which was omitted from the model).

No clear correlation between anatomical parameters or posture and translation directionality (i.e. orientation of main translation axis in glenoid XY) could be observed visually (see supplementary material, Appendix A figures A6, A7, A8, A9, A10).

### 3.5. Motion effect

Motion significantly affected 3D, AP and SI translations (*p*< 0.01, Appendix B in the supplementary material). Post hoc comparisons between motions show statistically lower SI translations in ERa90 compared to all other motions (figure 4, table 3). For 3D and AP translations (see supplementary material, Appendix A figures A2, A4), although statistical differences between motion pairs were detected, these effect sizes have large confidence intervals (approaching zero in some cases), and confidence interval bounds are within biplane measurement error in the 3D and AP translations (i.e. the confidence interval bound of 7.0% for ERaR versus FE translations in AP, given a mean translation magnitude of 3.9 mm, would be sub millimetre and, therefore, below biplane measurement error (see §4.8)). SI motion effects tended to have narrower confidence intervals farther away from zero, increasing confidence in these effects (i.e. clearer differences in the motion pairs in SI translations). For all motions, there were several subjects who deviated from the reported motion trend, showing opposite effects, with the exception of ERa90 versus FE, where all individuals had lower SI translation in ERa90 (figure 5; see supplementary material, Appendix A figures A3, A5).

**Figure 4.**
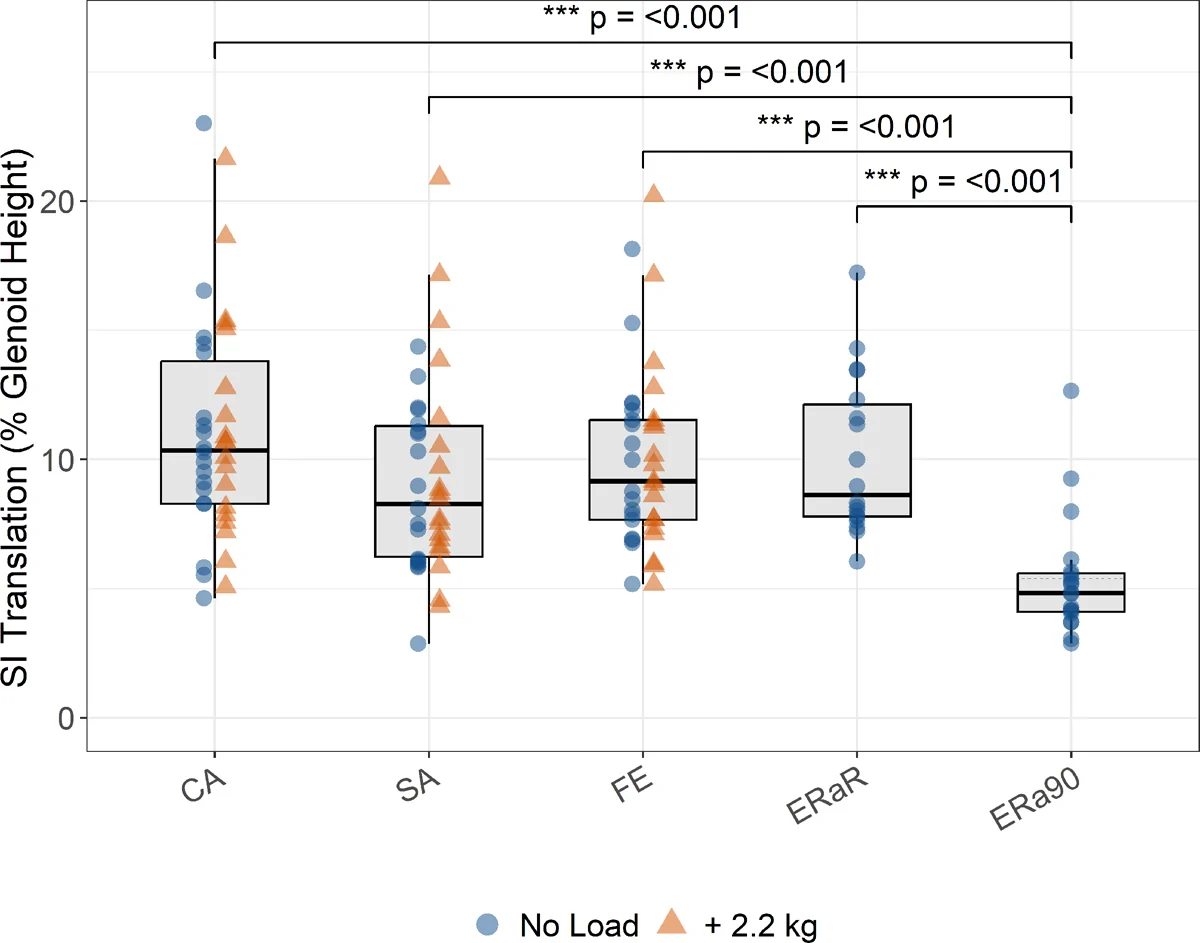
Comparison of superoinferior (SI) translations for all motions, with and without handheld load. Loaded and unloaded coronal abduction (CA), scapular abduction (SA) and forward elevation (FE) were pooled for significance analysis.

**Figure 5.**
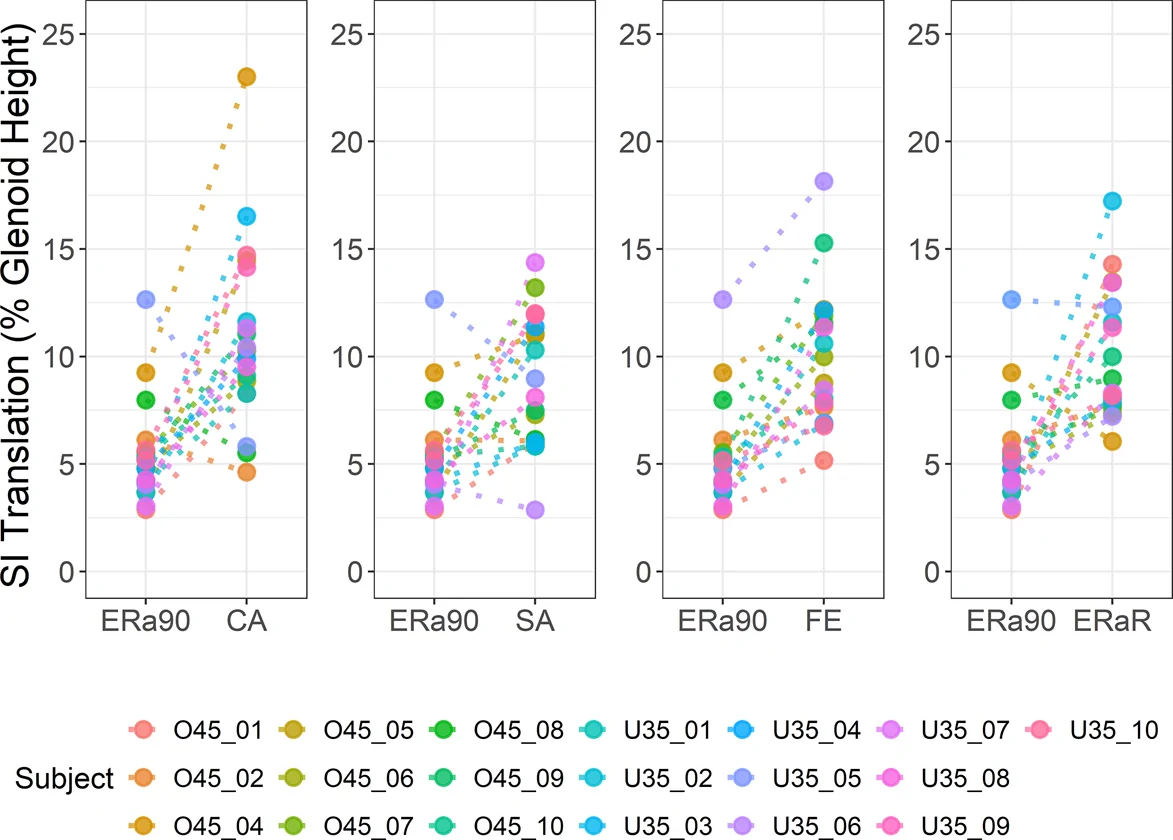
Pairwise comparisons of SI translations for significantly different motions. Dotted lines connect data for the same subject. SI = superoinferior, CA = coronal abduction, SA = scapular abduction, FE = forward elevation, ERaR = adducted internal/external rotation and ERa90 = abducted internal/external rotation.

**Table 3.** Significantly different motions. *CA = coronal abduction, SA = scapular abduction, FE = forward elevation, ERaR = adducted internal/external rotation and ERa90 = abducted internal/external rotation; % change indicates percentage change in overall translations.*

model	motion pair	% change	95% CI	*p*-value
3D	ERa90 < FE	–26.8	[–42.5%, –6.8%]	0.004
SI	ERa90 < CA	–50.5	[–62.4%, –34.8%]	<0.001
SI	ERa90 < SA	–39.5	[–54.1%, –20.2%]	<0.001
SI	ERa90 < FE	–46.0	[–59.1%, –28.7%]	<0.001
SI	ERa90 < ERaR	–48.2	[–61.9%, –29.6%]	<0.001
AP	FE > CA	26.3	[2.2%, 56.1%]	0.022
AP	FE > SA	24.6	[0.9%, 54.0%]	0.037
AP	ERaR < FE	–29.6	[–46.7%, –7.0%]	0.005

No clear correlation between motion and translation directionality could be observed visually, except an apparent decrease in SI translations in ERa90 (figure 6), which is supported by the statistical analysis (table 3). In ERa90, the main motion direction was AP in most subjects (figure 6; see supplementary material, Appendix A figure A26).

**Figure 6.**
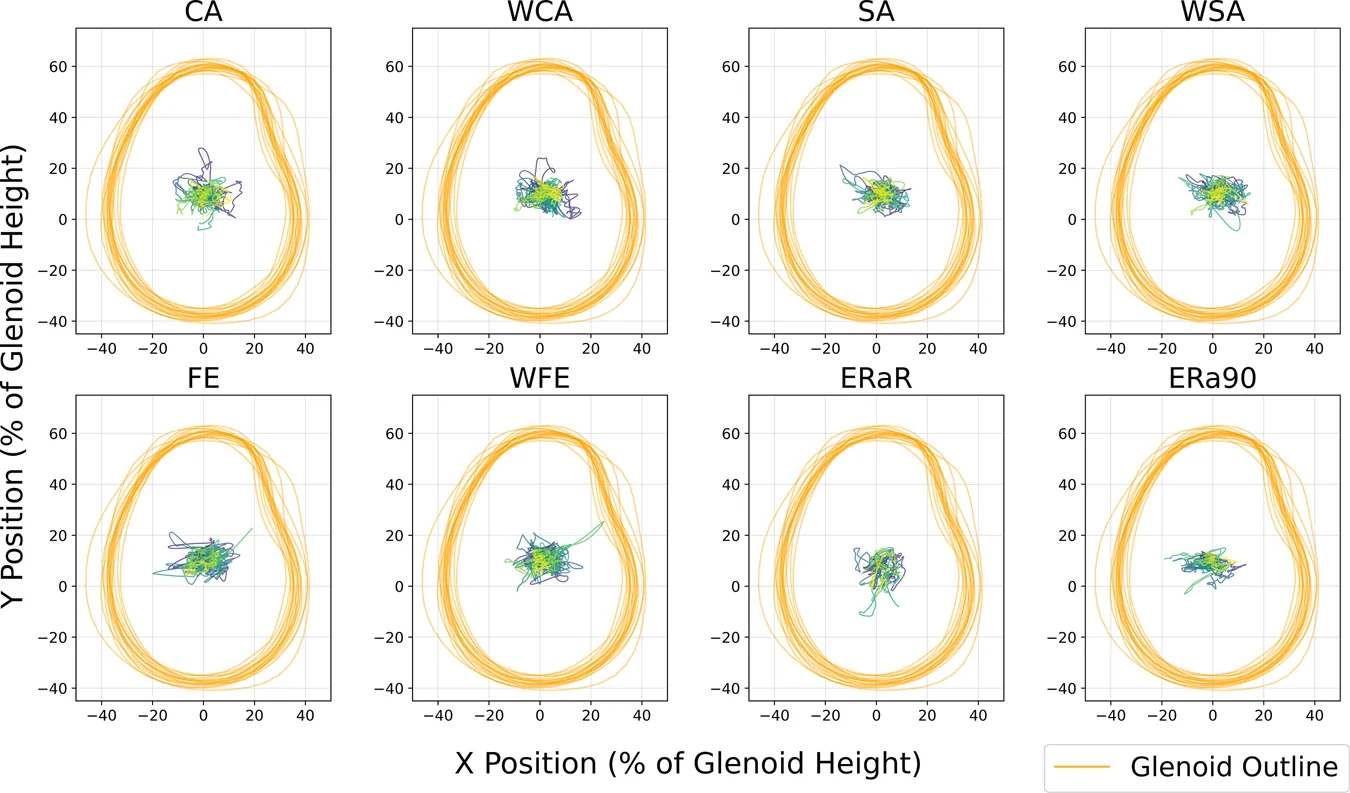
GH translations per motion, colour coded by subject. All subjects' glenoid coordinate system axes were aligned and centred based on the glenoid coordinate system centre used in this study (centre of inferior glenoid best-fit circle). CA = coronal abduction, SA = scapular abduction, FE = forward elevation, ERaR = adducted internal/external rotation and ERa90 = abducted internal/external rotation. CA, SA and FE were also performed with a 2.2 kg handheld load (WCA, WSA and WFE).

### 3.6. Handheld load

Handheld load did not significantly affect translations (figure 4; see Appendix A figures A2, A4 and Appendix B in the supplementary material).

### 3.7. Alignment of humeral head in the starting position

The starting position was closer to the geometric mean in the glenoid XY plane in 89.3% of trials, when pooling all subjects and motions (total of 150 trials) (figure 7). The mean Euclidean XY distance from the starting point to the geometric mean was 4.8% of the glenoid height and 1.6 mm in absolute terms (range: 0.3–20.5% glenoid height / 0.1–7.0 mm, s.d.: 3.1% glenoid height and 1.1 mm). The mean distance from the starting point to the centre of the inferior glenoid circle was 9.6% glenoid height and 3.2 mm in absolute terms (range: 1.6–16.6% glenoid height / 0.6–6.0 mm, s.d.: 2.9% and 1.0 mm). When assessing all motions separately, the starting point was closer to the geometric mean in the majority of cases for all motions (see Appendix A figure A28 and Appendix B in the supplementary material). We also tested whether these conclusions were sensitive to using the start versus end position (last frame) of the trial (both should be in a similar position, and comparing them indicates within subject variability of the ‘rest’ position pre and post trial). The start and end points differed on average by 3.3% glenoid height and 1.1 mm (range: 0.3–20.7% glenoid height / 0.1–6.4 mm, s.d.: 2.9% glenoid height and 0.9 mm) in terms of Euclidean distance in the XY direction (see supplementary material, Appendix A figures A11-25). Using the end point instead of the start point in the analysis of whether the humeral head centre was closer to the inferior circle centre or glenoid mean yielded similar results to using the start point (see supplementary material, Appendix A figure A29, and Appendix B). The end position was closer to the geometric mean in 88.7% of trials, when pooling all subjects and motions (total of 150 trials) (Appendix B in the supplementary material). Regarding anteroposterior positioning of the humeral head, the humeral head in the starting position was anterior to the glenoid midline in 72% of cases and the humeral head in the end position was anterior to the midline in 73% of cases (Appendix B in the supplementary material), but the mean distances to the midline were low with high s.d., demonstrating considerable variability between subjects (figure 7; see supplementary material, Appendix A figures A28, A29, and Appendix B).

**Figure 7.**
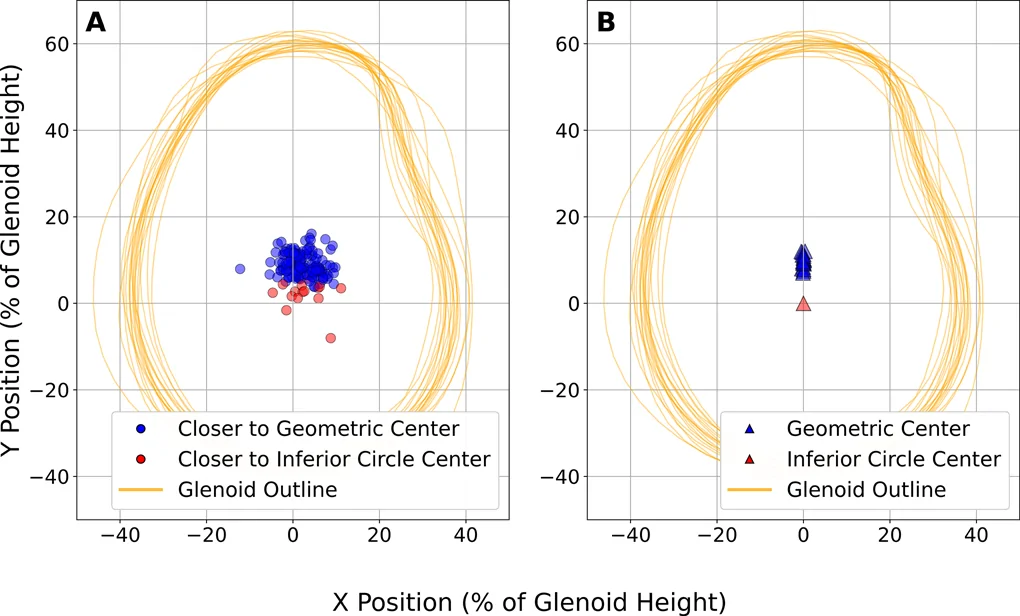
A) Starting point of humeral head centre projected to glenoid coordinate system for all motion trials and subjects, colour coded by whether this point is closer to the geometric mean of the glenoid boundary in the XY plane of the glenoid, or closer to the centre of the inferior best-fit circle. B) Glenoid geometric means and centre of inferior best-fit circle (by definition, this centre coincides with the origin of the glenoid coordinate system).

## 4. Discussion

The purpose of this study was to assess the translations of the humeral head centre relative to the glenoid in healthy subjects in a variety of motions, to comprehensively analyse subject-specific variability in joint kinematics and to test whether bony joint geometry and posture are predictors of translation variability. Our most important finding is the subject-specific variability and consistent subject-specific patterns across motions. Inclination angle, glenoid asphericity and scapular tilt affected 3D and AP translation magnitudes. Glenoid height influenced 3D and SI translations and motion type impacted translations in all directions (although future studies are needed to determine effect size because of the large confidence intervals, and because some confidence intervals approached zero). The starting point was closer to the geometric mean than the inferior glenoid best-fit circle in most trials. Future studies are needed to determine which morphological and neuromuscular features predict subject-specific translational patterns.

Our characterization of healthy GH translations and resting arm positioning is an important reference for studies investigating pathologies or testing effects of implants. Previous studies have used healthy GH translations as a baseline to determine which implant designs best restore GH kinematics [55]. Determining which motions and loads produce the highest translations enables evaluation of treatments/implant designs under ‘worst case’ scenarios where muscle stability is lowest. In addition, characterization of healthy translations is important for biomechanical model development, given that six-degree-of-freedom computational models often compare model translation patterns and magnitudes with experimental data as a form of validation (e.g. [56–60]).

### 4.1. Translation magnitude and pattern

The magnitudes of the AP and SI translations are in agreement with the literature. Previous studies report mean AP translations varying from 2 to 6 mm, SI translations from 1.7 to 2.6 mm and ML translations of 2.5 mm across arm abduction and rotation tasks including healthy subjects and asymptomatic contralateral shoulders of rotator cuff repairs [7,8,11,15]. When comparing our results to other studies, it is important to note that we included both the complete motion to the peak and return to the resting position, whereas most other analyses only report motion to the peak end pose. However, in our data, the start and end positions often, but not always, coincided very closely (see supplementary material, Appendix A figures A11– A26 and Appendix B). The s.d. of translations in this study was similar to the s.d. of 1–2 mm reported in other studies [9,10,14,15,21,22].

Larger AP translations than SI translations have been reported in healthy subjects during abduction and rotation, but the study only investigated five subjects [7]. In our study, mean differences, s.d. and ranges were similar between SI and AP. Our plots also show that higher AP than SI translations do not hold true for all subjects in CA and ERaR (e.g. see supplementary material, Appendix A figures A12, A24), but it does seem to be the case qualitatively for ERa90 (see supplementary material, Appendix A figure A26; where motion was mostly in the AP direction). Regarding the humeral head position relative to the glenoid throughout movement, Giphart *et al.* [9] reported that the humeral head was positioned on an average of 4–5 mm posterior to the glenoid midline during CA, SA and FE. However, in our study, some subjects translated anteriorly and others posteriorly (see supplementary material, Appendix A figures A11-A26), and the starting points were also not consistently more posterior (see §4.7).

We also observed that some individuals took a different path along the glenoid when moving away from the starting position compared with moving towards the end position (see supplementary material, figures 18–33 in Appendix A). Variation in humeral position between adduction and abduction has also been demonstrated by Graichen *et al.* [21] using MRI, although that study analysed static poses with isometric muscle activity to counter external loads. SHR has also been shown to differ during adduction versus abduction (both in unloaded cases, as well as concentric pull down versus push up exercises [13,61,62]). Future studies should investigate effects of scapular kinematics including SHR on GH joint translations, given that SHR has been proposed to provide GH joint stability [61,63] and that it varies between subjects [39].

Regarding the pattern of GH translations, Bey *et al.* [8] found that elevation angle has a significant effect on SI translation, with more superior mean translations in the first 20^∘^ of GH abduction followed by inferior translation. Graichen *et al.* [21] also found an initial superior increase followed by inferior translation in abduction. Nishinaka *et al.* [11] found that with increasing shoulder abduction, the humeral head on average translated more superiorly. However, these averages may obscure subject-specific patterns, and variation in reported mean patterns may be due to variation in the subjects investigated in the study. In our study, we found considerable subject-specific variation in translation pattern, indicating that there is not a consistent superior-inferior translation pattern during arm abduction.

### 4.2. Subject-specific variability

Our analysis demonstrates that subjects vary in both translation magnitude and pattern. This variability in directionality of GH translations should be considered in biomechanical studies, and, if present in pathological individuals, has implications for treatment design. Patient variation in osteoarthritis-associated glenoid wear [64], implant wear location [65] and translations in rotator cuff subjects [66], together with the variation in the healthy subjects analysed in this study, suggests that variability is also likely to be present in pathological subjects. Therefore, shoulder treatment design may benefit from personalization based on individual translation magnitudes and patterns, but further studies are needed to investigate subject variability in pathological subjects. The biomechanical effects of subject-specific translation patterns (i.e. on joint loading and bone stresses) remain uninvestigated.

### 4.3. Glenoid size effect

Most studies do not report translations relative to glenoid height. Glenoid height (mm) affected SI translation magnitude. A change of approximately 10.3 mm increase in glenoid height (from the mean) would be required to change SI translation magnitude by 1 mm from the mean (see Appendix B in the supplementary material for calculations; see §4.8 for context of biplane measurement error). Glenoid height also significantly affected 3D translation magnitude, but the *p-*value was at the significance threshold (*p* = 0.05) and the confidence interval included zero. 3D translations are also affected by ML translations which are probably dictated by muscular recruitment. Additional analyses (Appendix B in the supplementary material) suggest that the glenoid size effect is due to a scaling effect but future studies are needed to better determine the exact relationship.

### 4.4. Bony joint geometry and posture

Unlike previous studies [4,19], we did not observe an effect of GH congruence (ratio of humeral head radius to glenoid radius) on translations. However, these other studies assessed articular surface mismatch, whereas we only assessed bony geometry because the scanned data were not available for segmentation of the cartilage (and the CT scans resolution would probably be too low). Although the humeral head radius of curvature does not differ significantly between subchondral and cartilage based measurements, the glenoid bony radius of curvature is significantly larger than the glenoid radius of curvature when incorporating the cartilage [19] or cartilage and labral complex [18]. This variation in measurements between bony and overall geometry could explain why our study did not find an effect of joint congruence on translations. In addition, our study provided *in vivo* rather than *ex vivo* measurements and controlled for various other factors besides congruence, which may explain the variation in our results compared with these other studies. Including cartilage can also slightly change the location of the humeral head centre, resulting in significantly higher translations in some motions for the bone derived humeral head centres compared with the cartilage derived centres, but the differences in mean translations between the two methods remained less than 1 mm, which is within measurement error [19].

We found that glenoid asphericity (average distance of glenoid from surface of fitted sphere) affected 3D and AP translations. Regarding the effect size, changes of approximately 0.2 mm for 3D and approximately 0.1 mm for AP in glenoid asphericity from the mean would be needed to observe a 1 mm change in translation magnitude from the mean translation (see Appendix B in the supplementary material for calculations). These results must be interpreted with caution: the confidence interval for the percentage change in translations per percentage change in glenoid asphericity approaches 0% for 3D translation and both 3D and AP confidence intervals are large.

Inclination angle affected 3D and AP translation magnitudes, but the effect size was small (table 2). A change of at least 5^∘^ in inclination angle would be needed to observe a 1 mm change in translation magnitude from the sample mean 3D translation magnitude (4.9 mm) and a change of approximately 4^∘^ would be needed to increase AP translation magnitude by 1 mm from the sample mean (Appendix B in the supplementary material), but the confidence interval was large. Studies on larger datasets are warranted to clarify this effect, which may only be relevant in populations with considerable inclination variation. Other studies report decreased superior translation with lower glenoid inclination and decreased overall translation magnitude [23–25]. We found no correlation between inclination angle and SI GH translation magnitude. Translation pattern plots (see supplementary material, Appendix A figures A6, A7 and A10) also do not show a clear positional shift of the GH translations with inclination angle (although note that the translational pattern figures are not able to account for other factors in the way the statistical model can). The other *in vivo* study testing the effects of inclination angle also found no effect of inclination on SI translations [12], and neither did the cadaveric study of Knighton *et al.* [26].

Differences in the effect of inclination angle on SI translations can be due to several factors, including population studied, experimental approach (*in vivo* versus cadaveric versus in silico), as well as coordinate system definitions and inclination measurement methods. In *in vivo* studies, other subject-specific parameters, such as variation in musculature, may obscure the effect of inclination angle in the SI direction (although we did detect an effect in the AP and 3D directions). In addition, while our subjects varied in glenoid inclination angle, no subjects had a downward inclination, whereas some of the studies investigating inclination angle effects decreased the inclination angle to 30^∘^ [23]. Therefore, inclination angle may only affect superior migration of the humeral head once a certain downward inclination is achieved, and these inclinations may not be present in healthy subjects. Furthermore, inclination angle may only have an effect if the rotator cuff is compromised, since most studies reporting an effect of inclination angle investigated rotator cuff subjects ([23,56], but see [24]).

Finally, both this study and the other *in vivo* study [12] only measured the bony glenoid. However, incorporating the superior labral complex into glenoid inclination angle measurements results in a more downward inclination, and this effect is not constant, instead correlating with the original bony inclination angle [18]. Retroversion is also affected when including the labrum, increasing the retroversion compared to bony measurements [67], and the labrum adds passive stability to the joint [68,69]. In addition, anatomical variation of the adjacent capsuloligamentous complex must be considered as a possible factor accounting for inter-individual differences in GH translations, as GH ligaments together with the joint capsule provide passive stabilization of humeral head movements depending on the arm position [70]. Further studies are needed to explore correlations between *in vivo* GH translations and glenoid surface inclination and retroversion measures including soft tissue anatomy rather than just the bony anatomy, ideally controlling for variation in muscular recruitment and lines of action.

In this study, scapular tilt relative to the subject-global coordinate system statistically affected 3D translation magnitude, but the effect size was small (approx. 18.5^∘^ change in tilt required to change translations by 1 mm from mean, Appendix B in the supplementary material) and the confidence interval of the percentage change in translations per degree change in predictor was large with lower limits less than 0.5% translation change per degree change in scapular tilt (table 2). Therefore, further studies are needed to investigate this effect, and effects on translation may only be relevant when comparing subjects at the ends of the postural range (table 1). Furthermore, scapular tilt correlated with scapular upward rotation in our population, so any interpretation of scapular tilt effects might be due to correlations between translations and scapular upward rotation (indirectly captured by the correlation between scapular upward rotation and scapular tilt).

The movement of the humeral head relative to the glenoid is probably affected by a complex interplay between passive and active forces, which are in turn affected by anatomy and posture. The active force vectors affecting humeral movement relative to the glenoid are determined by subject-specific muscle orientation (which will be affected by the orientation of the glenoid relative to the scapula, as well as subject-specific anatomical features such as the acromion, both of which were not investigated in this study). One relevant passive force component is the gravity vector (which is influenced by the orientation of the glenoid in global space). In this study, we tested the effect of posture by measuring scapular orientation in global space to quantify scapular orientation relative to gravity. However, the orientation of the glenoid relative to the scapula may also vary between subjects, and could lead to different orientations of the glenoid relative to gravity. Such variation and the effects on the gravity vector should be explored further. Regardless, the gravity vector is unlikely to be the sole determinant of translation directionality, since the variability in translation directions is much more than the expected variation in glenoid orientation relative to the gravity vector (i.e. visually, the main translation axis direction between some subjects differs over 90^∘^, see supplementary material, Appendix A figure A12), and we expect that the inter-subject difference in glenoid orientation relative to the gravity vector would not be this extreme. Furthermore, while scapular tilt affected translation magnitude in the AP direction, this does not seem to be due to a simple reorientation of the main translation vector because of changes in the glenoid axes relative to the gravity vector (see supplementary material, Appendix A figure A8).

### 4.5. Motion effect

The effect of motion is relevant for both pathology diagnosis and treatment design; when investigating instability, impingement, lesion engagement or implant loosening cases, it is important to know which motions produce the highest translations and could, therefore, lead to complications. Giphart *et al.* [9] found that motion type (CA, FE or SA) significantly affected the amount of GH excursion (5.1 mm mean translation in CA versus 2.4 mm in scaption, although these motions were conducted by different subjects, so these effects could be due to subject-specific variability rather than motion variability). In our study, we accounted for subject variation when considering motion effects. Although various motion pairs differed statistically, the effect size confidence intervals approached zero or the biplane measurement error in 3D and AP translations, therefore we do not infer any clinical difference in these translation directions. The effect of motion type on SI translations was clearer (lower translations in ERa90 compared with ERaR and to arm raising movements, especially FE). A possible explanation for this phenomenon is that in high arm rotation, the muscle effort required to keep the arm elevated stabilizes the joint, whereas in low rotation, the rotator cuff may be more relaxed, enabling larger SI translations. In arm raising motions, more SI translations may occur because of the increased SI shear generated by the deltoid. These trends should be investigated in pathological subjects for future use in implant testing protocols (both virtually and physically). In cases where GH stability is a concern, motions that tend to have higher translations should be tested, but it should be kept in mind that the motions with highest translations in instability subjects may not be the same as in healthy subjects, and the directionality is important. For example, in anterior shoulder instability, shoulder dislocation or the sensation of instability typically occurs with the arm elevated to 90^∘^ and externally rotated [70].

### 4.6. Handheld load

Handheld loading did not affect GH translations, in agreement with Croci *et al.* [17]. This indicates that the musculature actively works to stabilize against external load in the healthy shoulder. It is possible that higher loads could affect GH translations (in this study, only 2.2 kg was added). Previous studies reported that external load affected scapular kinematics and SHR, but these rotational variations may coexist with no translational changes at the joint [28,29]. Indeed, Lee *et al.* [61] proposed that the GH joint may reorient in response to load to stabilize the joint, based on observations of variation in scapulothoracic orientation during different loading tasks.

### 4.7. Alignment of humeral head in the starting position

We found that the starting point of the humeral head (before initiation of the motion) was generally superior to the ‘inferior glenoid best-fit circle’, closer to the geometric midline of the glenoid (figure 7; see supplementary material, Appendix A figure A28). Using the end point instead as a rest position reference yielded similar results. Matsuki *et al.* [15] found that the starting position in SA was lower than the geometric mean but did not investigate the glenoid inferior circle. The starting-point position can serve as a healthy reference point to quantify humeral head decentring and potentially provide a target for restoring neutral humeral head position in shoulder surgery.

It is important to note that these starting positions may differ from the humeral head position in a relaxed resting position, since subjects may recruit muscles and change their external rotation in preparation for a specific motion task. Indeed, the starting points in ERa90 qualitatively exhibited less SI variability than other poses (see supplementary material, Appendix A figure A28), which is in agreement with the kinematic variability (fewer SI translations in ERa90 relative to other poses).

### 4.8. Limitations

This study has several limitations. First, all joint parameters were only quantified as bony geometry, rather than including cartilage, since we did not have access to high-resolution scans to segment the cartilage (see §4.4 about possible effect on congruence and glenoid angles). Our study analysed translation of the humeral head centre relative to the joint surface, which is more relevant to joint loading than quantification of translations in a scapular or global coordinate system. However, variation in humeral surface morphology may also affect joint contact patterns, and may not correlate with translations of the humeral head centre. Indeed, while humeral head translations did not differ significantly between repaired rotator cuff shoulders [8], the position of the *contact centre* did show a difference [13]. However, it is unclear whether these differences in contact point versus humeral head centre translations arise from variations in subjects between these two studies. Future work is needed to determine correlations between humeral head centre translations and GH contact patterns. Our study is also limited by the small sample size, which is inherent to the complexity of collecting biplanar fluoroscopy data. Although the subjects span a range of ages and have equal sex distribution, it is possible that a larger and more anatomically diverse dataset would yield different results.

We did not control for maximum humeral rotational excursion, which could account for some inter-subject variability, since the goal of this study was to investigate effects of static, clinically obtainable parameters on GH translations. Future analyses should investigate the effect of GH rotational excursion on GH translations, as well as the effect of scapular and thoracic kinematics. Regarding posture, we only investigated scapular global starting posture, and did not quantify thoracic posture. While scapular posture is a sum of thoracic-global posture and scapulothoracic posture, different scapulothoracic positions may also affect GH joint kinematics by changing muscular lines of action and should be investigated. We also did not investigate the effects of subject-specific muscle anatomy and recruitment in this study. The shoulder musculature actively stabilizes the GH joint via compressive forces but can also decentre the humeral head via shear forces [71–73]. Muscular recruitment has also been shown to significantly influence GH translations during arm elevation when comparing active versus passive movements [21]. Variation in muscular recruitment and lines of action as well as variation in strength distribution across muscles may therefore explain the subject-variability observed in this study.

In our study, we tested linear relationships with the outcome (glenoid translations) on a log scale. This approach enables assessment of multiplicative effects (e.g. percentage change in translation with percentage change in glenoid height). We did not use a generalized additive model with smooth functions to model more complex relationships because the effects in such models are difficult to interpret, and we did not hypothesize any complex nonlinear patterns. We acknowledge that posture could potentially have a quadratic effect (with deviations in either direction from a mean/standard condition, causing more aberrant translations). However, when investigating the fitted values versus residuals plots (Appendix B in the supplementary material), we do not see any clear effect of the fitted value from the linear model on residuals (model errors), meeting the linear model assumptions (if the residuals had changed with fitted values, this would have indicated that the linear model is not a good fit). The residuals also show no clear correlation with the posture values (Appendix B in the supplementary material residuals versus predictor plots—a correlation would be apparent if the relationship was quadratic but modelled as linear). Therefore, our study only investigated linear effects (in the log scale). We also did not model interaction effects between different parameters, due to the high number of possible combinations and no *a priori* hypotheses of which interactions would be present. Future studies should investigate this. Furthermore, we only modelled random intercepts but not random slopes for subjects, again to reduce complexity and following the assumption that subjects would respond similarly to different motions. Random slopes could be used to account for subject variability in the motion effect, which we observed in this study (i.e. while most subjects had similar responses to motion but different baselines (intercepts), some subjects did not follow the general motion trends, as seen in figure 5 and see supplementary material, figures 10 and 12 in Appendix A). Our data therefore suggest that incorporation of subject-specific slopes in future models may be appropriate to capture differences in subject-specific responses to motion.

Another limitation of the study is the possible error in biplane fluoroscopy. Biplane error is usually quantified against implanted titanium beads, the gold standard for *ex vivo* experiments (note that these beads can also contain small positional registration errors [74]). Mean translational errors of the humerus and scapula (beadless versus bead-based) using both automatic and manual registration range from 0.2–0.4 mm (s.d.: approx. 0.3–0.4 mm) [9,75–78]. These bone-wise errors would sum to almost 1 mm of translational error in the worst case scenario. Other studies have reported errors of 0.36–0.42 mm (s.d.: 0.29–0.32 mm) for GH translations [74]. The error itself also depends on the exact trial and matching. Ideally, multiple trials of the same motions would be performed to investigate inter-trial variation (although inter-trial variation can be affected by actual inter-trial differences, as well as inter-trial registration differences). However, due to the radiation involved in collecting biplane data, multiple trials of the same movements are usually not conducted when assessing different activities. However, we did observe subject-specific translation patterns consistent across similar arm movements (i.e. CA and SA, and FE and WFE (figure 3 and see supplementary material, figure 34 in Appendix A)). This indicates that (i) some individuals have translation patterns that persist across trials and (ii) the variation in movement patterns reported in this paper is not simply due to slight differences in bone registration to the biplane images. In addition, biplane fluoroscopy remains the most accurate technique for analysing bone kinematics *in vivo*, and all studies investigating translations are limited by the small errors inherent in these measurements, as well as the small sample sizes, due to radiation concerns.

Finally, it must be kept in mind that posture, inclination angle and version measurements may be sensitive to variation in GC definition, as well as definitions of the scapular coronal plane. The GC can be defined based on the inferior circle centre [47,48] or the glenoid surface mean projected to the glenoid surface [79]. Two-dimensional and 3D measurements, as well as different measurement approaches of version and inclination, yield different results, at least for some subjects [46,50,80]. The scapular coronal plane for 3D measurements is often based on TS, GC and IA [46,50], with the Z-axis being the line between TS and GC, but other studies have used the supraspinatus fossa to avoid dependence on the GC, which can be difficult to define in cases of osteoarthritis [79]. These two plane definitions differed by a mean of 6^∘^ [79]. Future studies should test whether different definitions of inclination, retroversion and posture show any correlation with translations, since different geometric definitions incorporate different aspects of subject-specific anatomy, especially given that we found the humeral head centre was closer to the geometric mean than the inferior best-fit circle. Similarly, different postural definitions (i.e. different coordinate systems, using Euler angle sequences instead of the clinical projection measurements, or analysing scapulothoracic instead of scapular global posture), may yield different results [40,81]. Note that the posture angle projection method is limited because the orientation in one postural rotation (e.g. internal rotation) affects measurements of the other postural parameters (e.g. upward rotation). However, Euler sequences also have this issue of inter-dependency [40]. We used posture angle projections to increase clinical interpretability and consistency with clinical posture studies. Future studies should test the effects of different posture definitions on study results.

## 5. Conclusion

In subjects with healthy shoulders, on average the humeral head centre translated less than 5 mm (15% of glenoid height) despite low bony constraints in this joint, a magnitude that is similar to that reported in other studies. The main finding of this study is the subject-specific variability in translation magnitude and directionality. These subject-specific patterns were preserved across similar motion trials, demonstrating that they are not artefacts of registration variation between trials. Motion affected translations, which is important to consider in studies investigating joint stability. Glenoid height affected 3D and SI translations, highlighting the importance of reporting GH translations relative to glenoid size in future studies. Inclination angle, scapular tilt and glenoid asphericity increased GH translation magnitude statistically, but these effects must be interpreted with caution because effect sizes remain small and confidence intervals are large, warranting further investigation. The variability in GH joint biomechanics should be considered both in the context of foundational functional biomechanics research as well as clinical applications to facilitate personalized treatments. Prediction of subject-specific patterns remains a challenge, and the effect of other subject-specific parameters (musculature, soft tissue contributions to joint geometry and scapular shape variation) should be investigated further to better understand the links between form and kinematics in the GH joint. The resting position of the humeral head across subjects and motions was closer to the glenoid geometric mean than the centre of the inferior glenoid circle in most cases, providing insights into healthy GH alignment at rest.

## Supplementary Material



## Data Availability

Statistical code and data for statistical analyses can be found on Zenodo [82]. Supplementary material is available online [83].
