# Supplementary material for: Determination of inter-individual differences in humeral head centre translations in subjects with healthy shoulders
Source: R Soc Open Sci. 2026 Oct 7;13(10):252309. doi: 10.1098/rsos.252309 (PMC13641899; doi:10.1098/rsos.252309)
Supplement: Supplementary file 1 [file rsos.252309.zip › rsos.252309/rsos252309_si_001.pdf]

Appendix B: Statistical Analysis Glenohumeral Translations

Load and Prepare Data

Note: the maximum translation range (column "max__translation__[motion]") was defined as the maximum Euclidean distance between any two positions of the humeral head in the glenoid coordinate system throughout the motion trial. "radiusRatio" refers to the ratio of humeral head versus glenoid radius of curvature (i.e. "joint congruence") and Mean__Distance__Glenoid__Sphere and Mean__Distance__HH__Sphere are the mean absolute difference from the glenoid and HH to a sphere fitted to these regions (to assess asphericity - lower values indicate more spherical fit).

```
# Set all FE motion data to NA for subject U35_010_M_27 since there was a recording gap
↪  in this trial (Aliaj et al. 2021, 2022)
# This includes both translation data and starting point data
data_raw$max_translation_FE[data_raw$Subject == "U35_010_M_27"] <- NA
data_raw$X_translation_FE[data_raw$Subject == "U35_010_M_27"] <- NA
data_raw$Y_translation_FE[data_raw$Subject == "U35_010_M_27"] <- NA
data_raw$Z_translation_FE[data_raw$Subject == "U35_010_M_27"] <- NA

# Also exclude starting point and endpoint data for FE motion for this subject
if ("StartingPoint_FE" %in% colnames(data_raw)) {
  data_raw$StartingPoint_FE[data_raw$Subject == "U35_010_M_27"] <- NA
}
if ("EndPoint_FE" %in% colnames(data_raw)) {
  data_raw$EndPoint_FE[data_raw$Subject == "U35_010_M_27"] <- NA
}

# note also that max_translation_ERaR was missing from the database for U35_001_F_26 so
↪  that value is NA already

# drop static motions because we are not analysing them
analysis_data <- data_raw[, !colnames(data_raw) %in%
  c("max_translation_IRaB", "max_translation_IRaM", "max_translation_static",
    "X_translation_IRaB", "X_translation_IRaM", "X_translation_static",
    "Y_translation_IRaB", "Y_translation_IRaM", "Y_translation_static",
    "Z_translation_IRaB", "Z_translation_IRaM", "Z_translation_static",
    "StartingPoint_IRaB", "StartingPoint_IRaM", "StartingPoint_static",
    "EndPoint_IRaB", "EndPoint_IRaM", "EndPoint_static")]


# Print first few rows of dataframe and first 15 columns to check the data structure
# head(analysis_data[, 1:15])
```

Reshape Data

Then, we reshape the data. Previously, we had a column per subject with the translation range for each motion (i.e. max__translation__CA, max__translation__FE, etc) and starting point coordinates for each motion

(i.e. StartingPoint_CA, StartingPoint_FE, etc). Since we explore effects of motion, we instead want to convert motion to a factor, so we split the max_translation_[motion] and StartingPoint_[motion] into the max_translation, StartingPoint and motion components.

```r
# Reshape the data from wide to long for all translation measures (remove StartingPoint
↪  and EndPoint since we don't need them in the main pivoted long format dataframe and
↪  use them in a different analysis later)
analysis_data_long <- analysis_data %>%
  select(-starts_with("StartingPoint_"), -starts_with("EndPoint_")) %>%
  pivot_longer(cols = starts_with(c("max_translation_", "X_translation_",
   ↪  "Y_translation_", "Z_translation_")),
               names_to = c(".value", "motion"),
               names_pattern = "(.*?_[^_]+)_(.*)"
  )


# Add normalized translation columns
# Overall 3D translations, Y translations (SI direction), and Z translations (ML
↪  direction) are normalized by glenoid height
# X translations (AP direction) normalized by glenoid width since AP translations relate
↪  more to width
analysis_data_long$normalized_max_translation <- (analysis_data_long$max_translation /
↪  analysis_data_long$GlenoidHeight) * 100
analysis_data_long$normalized_X_translation <- (analysis_data_long$X_translation /
↪  analysis_data_long$GlenoidWidth) * 100
analysis_data_long$normalized_Y_translation <- (analysis_data_long$Y_translation /
↪  analysis_data_long$GlenoidHeight) * 100
analysis_data_long$normalized_Z_translation <- (analysis_data_long$Z_translation /
↪  analysis_data_long$GlenoidHeight) * 100

# Consolidated long-format dataset: 3D, X, Y, and Z translation measures in one frame.

# Now, we want to add "weight" as a factor (ie if the motion was loaded or unloaded). CA,
↪  SA, and FE have unloaded and loaded trials, the loaded ones have "W" in the name. We
↪  now want to reformat the dataframe to have "base_motion" columns and then a binary
↪  true/false for whether weight was added

analysis_data_long_weight_factor <- analysis_data_long %>%
  mutate(
    weight = startsWith(as.character(motion), "W"),
    base_motion = ifelse(weight, substring(as.character(motion), 2),
     ↪  as.character(motion))
  )

# Reorder base_motion factor levels for plotting
analysis_data_long_weight_factor$base_motion <-
↪  factor(analysis_data_long_weight_factor$base_motion,
                                                        levels = c("CA", "SA", "FE", "ERaR",
                                                         ↪  "ERa90"))

# Display the structure of the reshaped data (showing last 5 columns)
head(analysis_data_long_weight_factor[,
↪  (ncol(analysis_data_long_weight_factor)-4):ncol(analysis_data_long_weight_factor)])
```

```
# A tibble: 6 x 5
  normalized_X_translation normalized_Y_translat~1 normalized_Z_transla~2 weight
                     <dbl>                   <dbl>                  <dbl> <lgl>
1                     8.20                    8.29                   7.03 FALSE
2                    10.0                     5.17                   9.98 FALSE
3                     8.24                    5.99                   6.80 FALSE
4                     6.58                   14.3                    5.71 FALSE
5                     8.28                    2.88                   3.60 FALSE
6                     7.96                    7.85                   7.97 TRUE
# i abbreviated names: 1: normalized_Y_translation, 2: normalized_Z_translation
# i 1 more variable: base_motion <fct>
```

```
# Starting point data will be handled separately in the starting point analysis cell
```

Data Distribution Diagnostics

Let's make some histograms to check the data distribution. We use ggplot with Y = "Count". For brevity, we do not echo the histogram code.

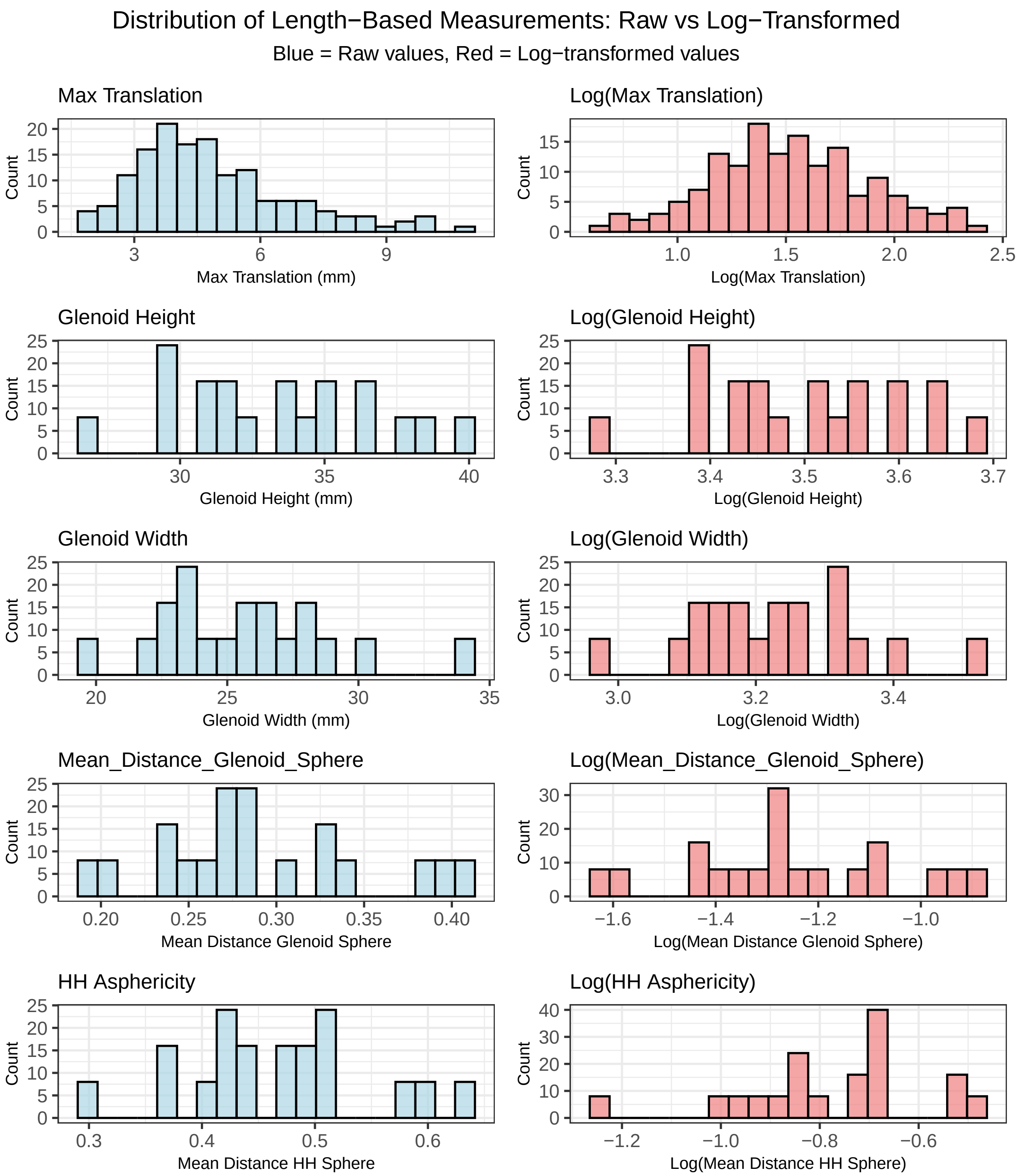

3D Translations vs Fixed Factors

Let's plot each factor vs max translations (as % glenoid height) to look at the data. We use % glenoid height to normalize the data against size differences (assuming there is a scaling effect, which we test later). For our analysis, we log transform all absolute measurements (max translation, glenoid height, glenoid width, and asphericity measures) to better interpret the coefficient effects of these predictors on max_translations and to normalize their distributions.

Loess smoothers are rendered in red. Note these plots give some insight into the data but they do not account for effects of other factors (besides height) - these other effects are included in our statistical model and residuals plots.

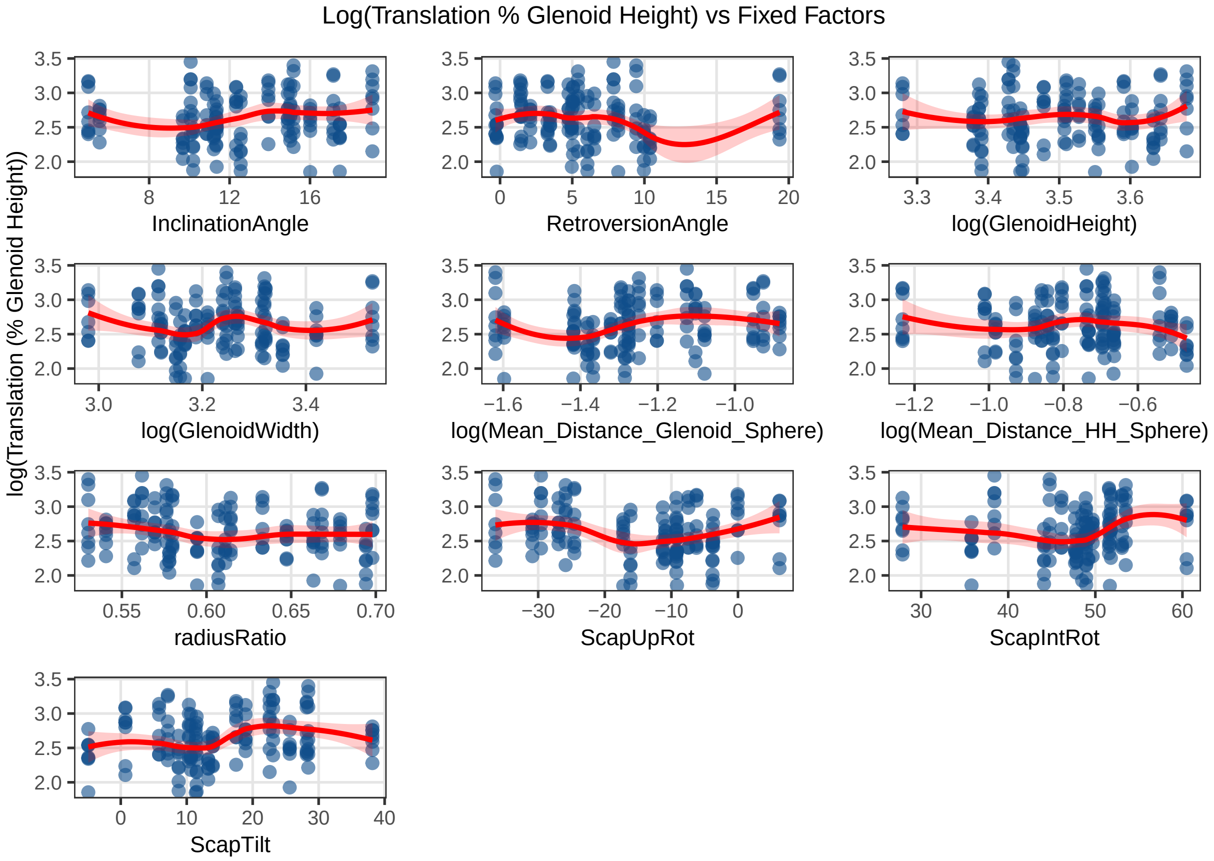

Initial lmer model fit for VIF (variance inflation factor) analysis

Here we check for multicollinearity among predictors. Note for all of our lmer models we log transform max_translations. We also log transform length measurements in the predictors (glenoid height, glenoid width, and asphericity measures) to better interpret the coefficient effects of these predictors on max_translations and to normalize their distributions.

Important Note on Size Controls: - *Overall 3D translations and Y translations (SI direction)*: Use glenoid height as size control - *X translations (AP direction)*: Use glenoid width as size control, since AP translations are more related to glenoid width than height

```
# first check for multicollinearity among predictors in our lmer model with all factors
↪  using VIF.

lmer_for_vif = lmer(log(max_translation) ~ InclinationAngle + RetroversionAngle +
↪  base_motion + weight + log(Mean_Distance_Glenoid_Sphere) +
↪  log(Mean_Distance_HH_Sphere) + log(GlenoidHeight) + log(GlenoidWidth) + radiusRatio +
↪  ScapUpRot + ScapIntRot + ScapTilt + (1 | Subject), na.action = na.exclude, data =
↪  analysis_data_long_weight_factor)
```

```r
# Calculate VIF values
vif_values <- vif(lmer_for_vif)
    print(vif_values)
```

```
                                     GVIF Df GVIF^(1/(2*Df))
InclinationAngle                 3.767013  1        1.940880
RetroversionAngle                3.231731  1        1.797702
base_motion                      1.252836  4        1.028577
weight                           1.251583  1        1.118742
log(Mean_Distance_Glenoid_Sphere) 2.001990  1        1.414917
log(Mean_Distance_HH_Sphere)     1.837145  1        1.355413
log(GlenoidHeight)               5.097069  1        2.257669
log(GlenoidWidth)                6.842306  1        2.615780
radiusRatio                      2.035955  1        1.426869
ScapUpRot                        6.380785  1        2.526022
ScapIntRot                       1.828810  1        1.352335
ScapTilt                         5.483131  1        2.341609
```

Glenoid height, glenoid width, scapular tilt, and scapular upward rotation all have VIFs above 5 (these parameters are correlated with another predictor or linear combination of predictors; generally VIF above 5 means remove if possible). Logically it makes sense that width and height are correlated. ScapUpRot and ScapTilt also have VIF above 5 and it makes sense that they may be correlated. However, to be sure we are not missing any more unexpected correlations (glenoid size with posture) we explicitly check the correlations of these parameters before deciding which factors to remove from the lmer due to multicollinearity. Let's plot all pairwise combinations of these 4 factors where VIF $>$ 5.

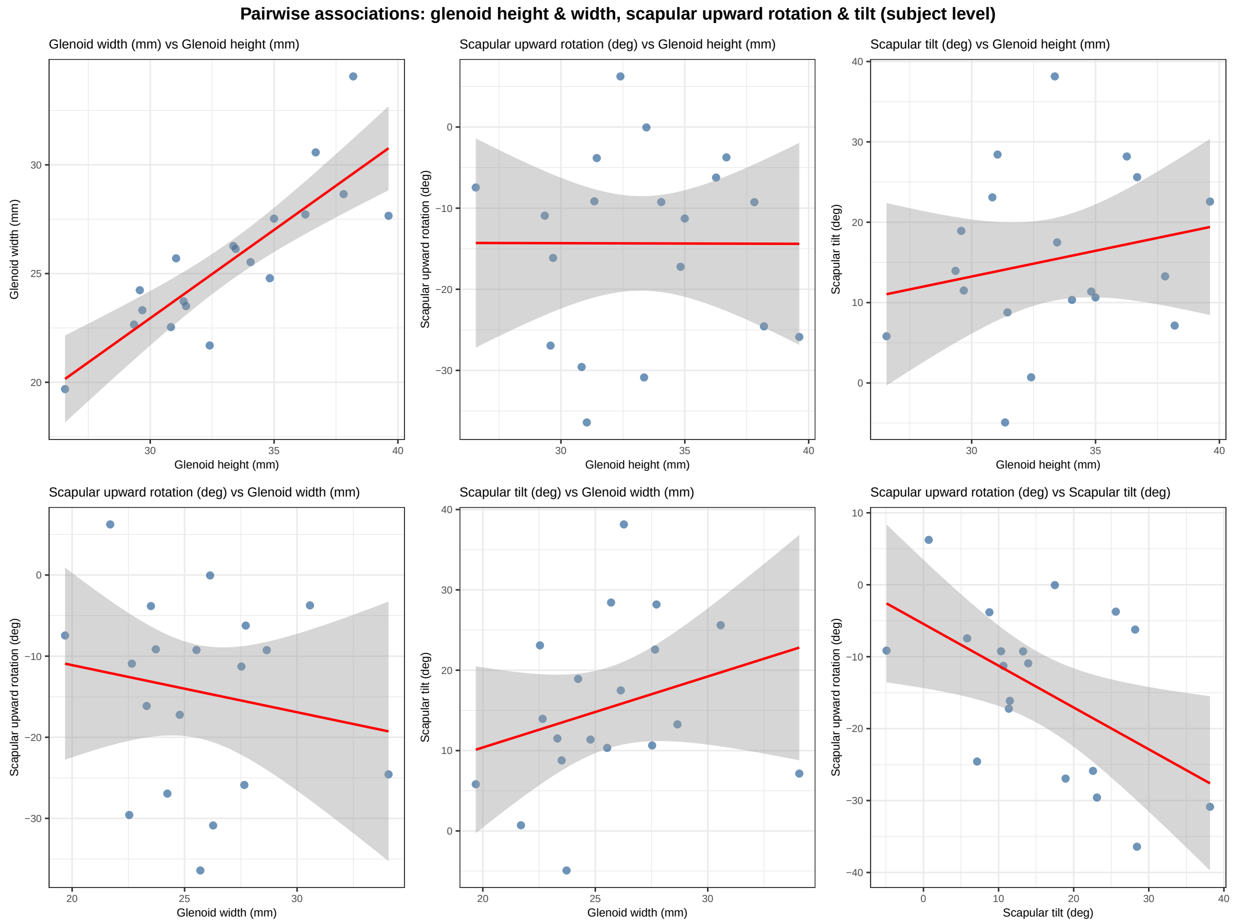

Based on the plots above, it appears that glenoid height and width are correlated and scapular tilt and scapular upward rotation are correlated. Let's check the correlations with linear regressions.

Glenoid height and width correlation:

```
Call:
lm(formula = GlenoidWidth ~ GlenoidHeight, data = analysis_data)

Residuals:
    Min      1Q  Median      3Q     Max
-3.2040 -0.6746 -0.3158  0.6102  4.4611

Coefficients:
              Estimate Std. Error t value Pr(>|t|)
(Intercept)    -1.4295     4.2362  -0.337     0.74
GlenoidHeight   0.8126     0.1268   6.408 6.49e-06 ***
---
Signif. codes:  0 '***' 0.001 '**' 0.01 '*' 0.05 '.' 0.1 ' ' 1

Residual standard error: 1.869 on 17 degrees of freedom
Multiple R-squared:  0.7072,    Adjusted R-squared:   0.69
F-statistic: 41.06 on 1 and 17 DF,  p-value: 6.489e-06
```

```
Coefficient = 0.813, 95% CI [0.545, 1.080], p = <0.001
```

Scapular upward rotation and tilt correlation.

```
Call:
lm(formula = ScapUpRot ~ ScapTilt, data = analysis_data)

Residuals:
     Min       1Q   Median       3Q      Max 
-14.9777  -6.9440   0.3485   5.3064  16.5834 

Coefficients:
            Estimate Std. Error t value Pr(>|t|)  
(Intercept)  -5.4462     4.2284  -1.288   0.2150  
ScapTilt     -0.5810     0.2293  -2.533   0.0214 *
---
Signif. codes:  0 '***' 0.001 '**' 0.01 '*' 0.05 '.' 0.1 ' ' 1

Residual standard error: 10.26 on 17 degrees of freedom
Multiple R-squared:  0.2741,	Adjusted R-squared:  0.2314 
F-statistic: 6.418 on 1 and 17 DF,  p-value: 0.02143
```

```
Coefficient = -0.581, 95% CI [-1.065, -0.097], p = 0.021
```

Based on the VIF factors and correlations above, we will exclude glenoid width and keep glenoid height. Let's rerun VIF with our new model excluding glenoid width to make sure this fixes the multicollinearity between glenoid width and height. We also exclude scap up rot (but keep in mind that any significance in tilt may really be due to significance in scap up rot since the two are correlated).

```
# rerun vif
lmer_for_vif = lmer(log(max_translation) ~ InclinationAngle + RetroversionAngle +
↪  base_motion + weight + log(Mean_Distance_Glenoid_Sphere) +
↪  log(Mean_Distance_HH_Sphere) + log(GlenoidHeight) + radiusRatio + ScapIntRot +
↪  ScapTilt + (1 | Subject), na.action = na.exclude, data =
↪  analysis_data_long_weight_factor)

# Calculate VIF values
vif_values <- vif(lmer_for_vif)
print("new VIF values for 3D model:")
```

```
[1] "new VIF values for 3D model:"
```

```
print(vif_values)
```

```
                                      GVIF Df GVIF^(1/(2*Df))
InclinationAngle                  1.764036  1        1.328170
RetroversionAngle                 1.382623  1        1.175850
base_motion                       1.252680  4        1.028561
weight                            1.251528  1        1.118717
log(Mean_Distance_Glenoid_Sphere) 1.612952  1        1.270020
```

```
log(Mean_Distance_HH_Sphere)       1.687827  1         1.299164
log(GlenoidHeight)                 1.543742  1         1.242474
radiusRatio                        1.244096  1         1.115391
ScapIntRot                         1.507569  1         1.227831
ScapTilt                           1.668350  1         1.291646
```

Great, our VIF values are now below 5 for all predictors.

Note: VIF analysis for AP and SI translation models will be performed later on in the code for those models.

Main Model Fitting and Analysis

Fit LMER Model for Overall Max Translation (3D) - removing glenoid width and scap up rot because of correlations between predictors

```
lmer_main = lmer(log(max_translation) ~ InclinationAngle + RetroversionAngle +
↪  base_motion + weight + log(Mean_Distance_Glenoid_Sphere) +
↪  log(Mean_Distance_HH_Sphere) + log(GlenoidHeight) + radiusRatio + ScapIntRot +
↪  ScapTilt + (1 | Subject), na.action = na.exclude, data =
↪  analysis_data_long_weight_factor)

# Save residuals for Model
analysis_data_long_weight_factor$residuals_3D <- resid(lmer_main)
```

Model Assumption Checks

Before further interpretation of our model, we check the assumptions of normality of residuals, linearity, and homoscedasticity.

QQ Plot for 3D Translations LMER

```
qqnorm(analysis_data_long_weight_factor$residuals_3D, col = "dodgerblue4", cex = 0.55)
qqline(analysis_data_long_weight_factor$residuals_3D, col = "red")
```

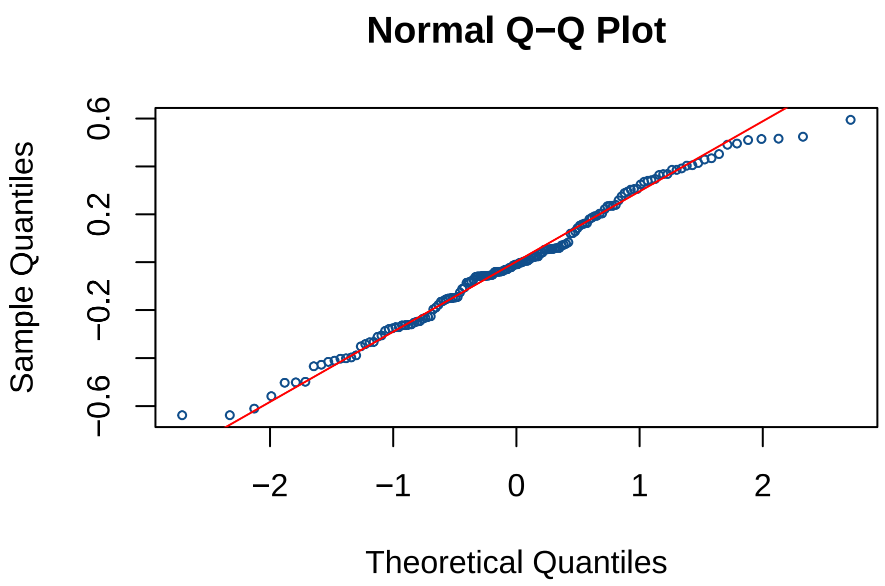


```
# Store plot for saving later
qqplot_saved <- recordPlot()
```

Interpretation Q-Q Plot: *The residuals appear to be normally distributed.*

Tukey-Anscombe Plot for 3D Translations LMER

```
tukey_plot <- ggplot(mapping = aes(y = resid(lmer_main),
                x = fitted(lmer_main))) +
  geom_abline(intercept = 0, slope = 0, color = "red", linetype = "dashed") +
  geom_point(alpha = 0.6, color = "dodgerblue4") +
  geom_smooth(method = "loess", method.args = list(family = "symmetric"), se = TRUE,
  ↪  color = "black", linewidth = 0.55) +
  labs(title = "3D Model",
       x = "Fitted Values",
       y = "Residuals") +
  theme_bw() +
  theme(
    plot.title = element_text(size = 9),
    axis.title = element_text(size = 8),
    axis.text = element_text(size = 7)
  )

print(tukey_plot)
```

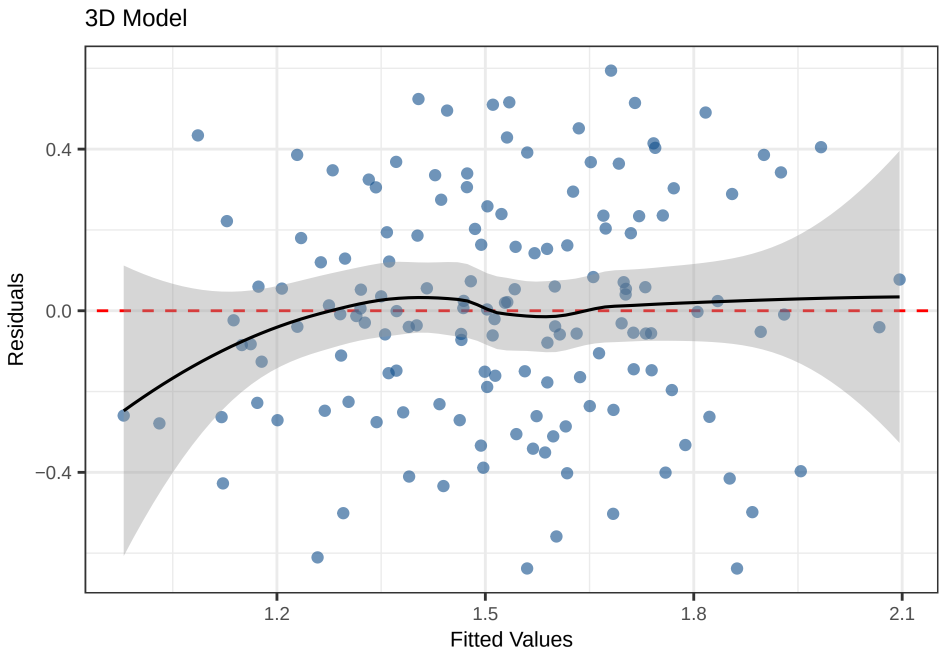

Interpretation of Tukey-Anscombe Plots: *The smoothers here appear centered on 0 across fitted values, supporting the linear model assumptions. The decrease and increase in the smoothers at the lowest and highest fitted values in the plots are likely due to the scarcity of the data in these regions (only 1-2 observations, which affect the smoother - the uncertainty of the smoother in these regions also increases due to low number of observations). The data also looks homoscedastic (equal variance of residuals across fitted values)*

Scale-Location Plot for 3D Translations

```
scale_loc_plot <- ggplot(mapping = aes(y = sqrt(abs(resid(lmer_main))),
                                        x = fitted(lmer_main))) +
  geom_point(alpha = 0.6, color = "dodgerblue4") +
  geom_smooth(method = "loess", method.args = list(family = "symmetric"), se = TRUE,
  ↪  color = "black", linewidth = 0.55) +
  labs(title = "3D Model",
       x = "Fitted Values",
       y = expression(sqrt(abs("Residuals")))) +
  theme_bw() +
  theme(
    plot.title = element_text(size = 9),
    axis.title = element_text(size = 8),
    axis.text = element_text(size = 7)
  )

print(scale_loc_plot)
```

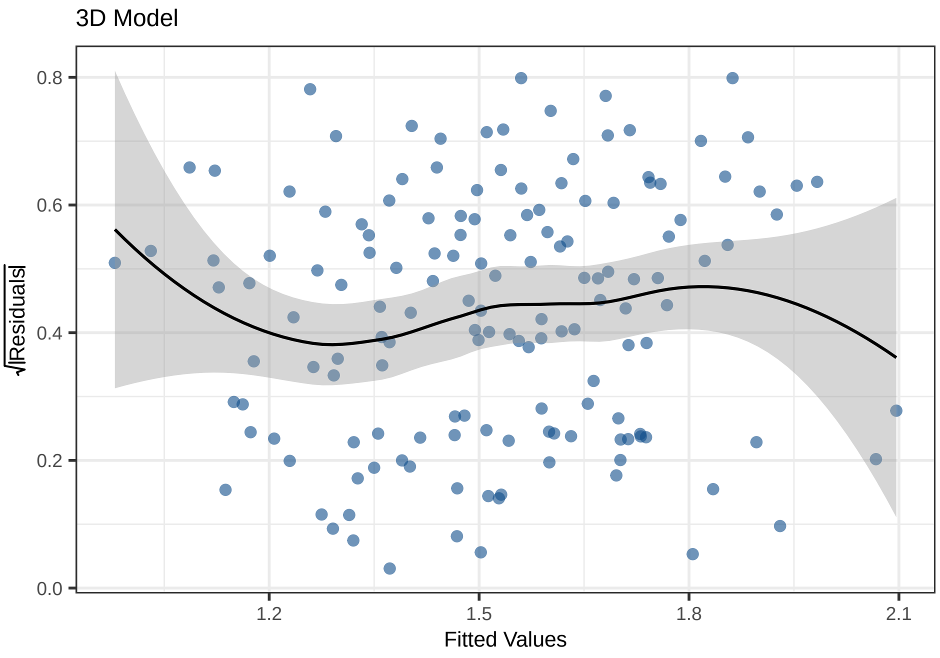

Interpretation of Scale-Location Plots: *The variance of the residuals seems to be fairly constant, confirming homoscedasticity (equal variance of residuals, i.e. the fitted values do not appear to affect the spread of the residuals). Again, the changes at the ends of the plots are likely due to the low sample size in these regions/natural randomness of the data (the uncertainty of the smoothers in these regions also increases due to low number of observations).*

Residuals vs Predictors

These plots help us test both the variance and also linearity (i.e. does the predictor value affect the response)

```
# Create the motion and subject plots using lattice (smaller versions - 90% of current
↪  size)
par(mfrow = c(2, 2), mar = c(2.4, 2.4, 1.6, 0.8))

resid_motion_plot <- lattice::xyplot(analysis_data_long_weight_factor$residuals_3D ~
↪  base_motion, data = analysis_data_long_weight_factor, jitter.x = TRUE,
       abline = 0, type = c("p", "g"), col.line="black",
       main = "Residuals vs Motion (3D LMER)", xlab = "Motion", ylab = "Residuals",
       par.settings = list(plot.symbol = list(col = adjustcolor("dodgerblue4", alpha.f =
        ↪  0.5), pch = 19, cex = 0.49)),
       scales = list(x = list(cex = 0.57), y = list(cex = 0.57)))

resid_subject_plot <- lattice::xyplot(analysis_data_long_weight_factor$residuals_3D ~
↪  Subject, data = analysis_data_long_weight_factor, jitter.x = TRUE,
       abline = 0, type = c("p", "g"), col.line="black",
       main = "Residuals vs Subject (3D LMER)", xlab = "Subject", ylab = "Residuals",
       scales = list(x = list(cex = 0.24, rot = 45), y = list(cex = 0.57)),
       par.settings = list(plot.symbol = list(col = adjustcolor("dodgerblue4", alpha.f =
        ↪  0.5), pch = 19, cex = 0.49)))

# Create weight plot with binary categories
```

```r
resid_weight_plot <- lattice::xyplot(analysis_data_long_weight_factor$residuals_3D ~
↪  factor(weight, levels = c(FALSE, TRUE), labels = c("No Weight", "With Weight")),
       data = analysis_data_long_weight_factor, jitter.x = TRUE,
       abline = 0, type = c("p", "g"), col.line="black",
       main = "Residuals vs Added Weight (3D LMER)", xlab = "Weight Condition", ylab =
       ↪  "Residuals",
       scales = list(x = list(cex = 0.57), y = list(cex = 0.57)),
       par.settings = list(plot.symbol = list(col = adjustcolor("dodgerblue4", alpha.f =
       ↪  0.5), pch = 19, cex = 0.49),
                           add.text = list(cex = 0.8)))

# Print these individual plots
print(resid_motion_plot)
```

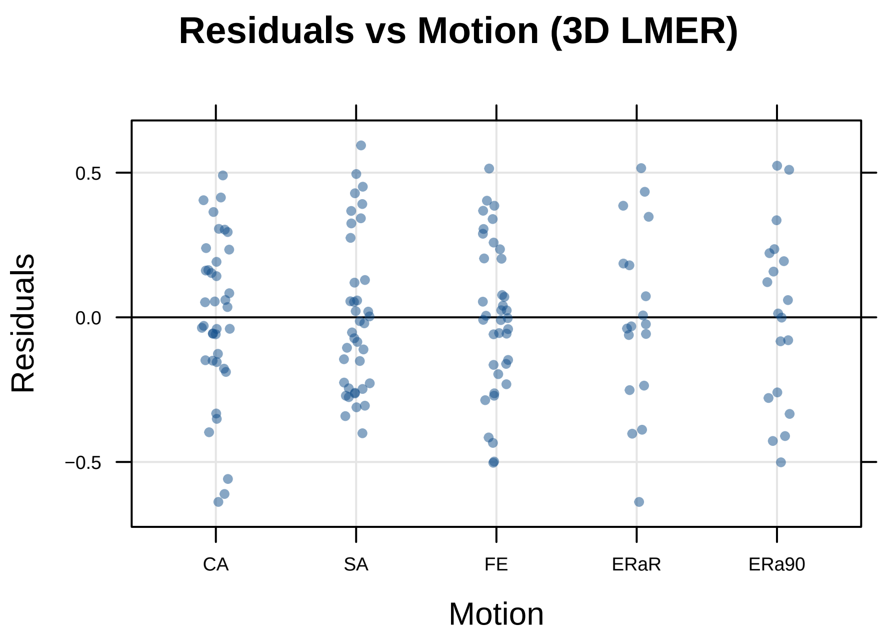


```r
print(resid_subject_plot)
```

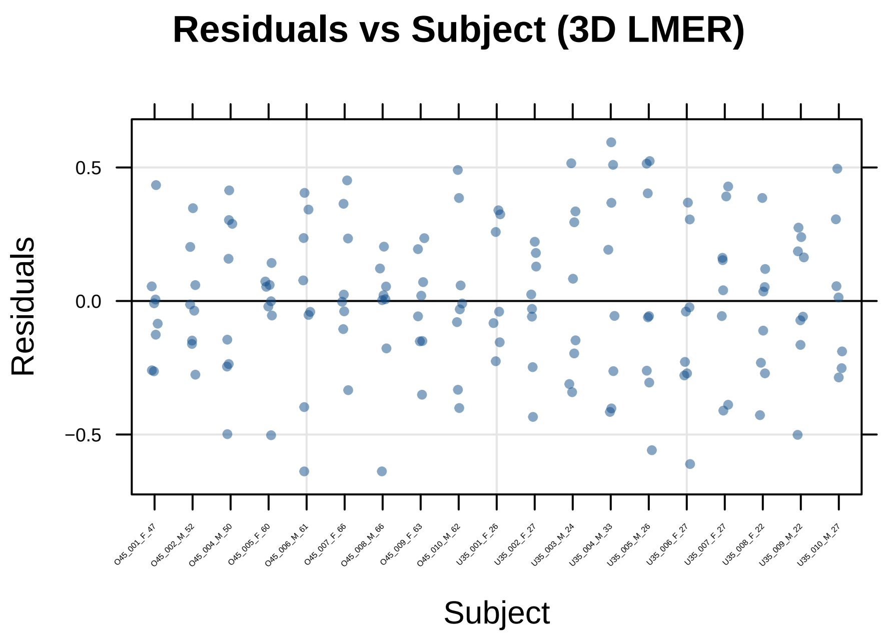


```r
print(resid_weight_plot)
```

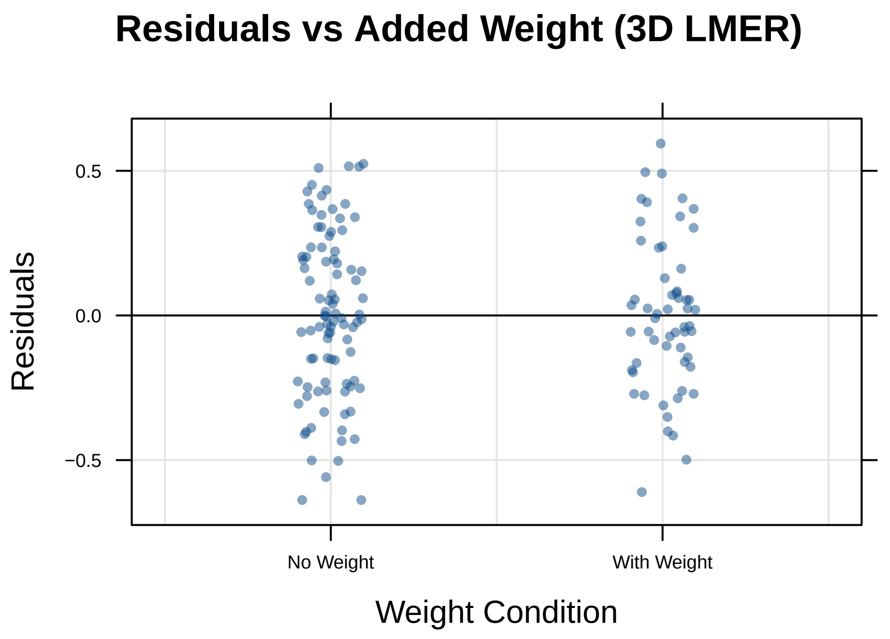

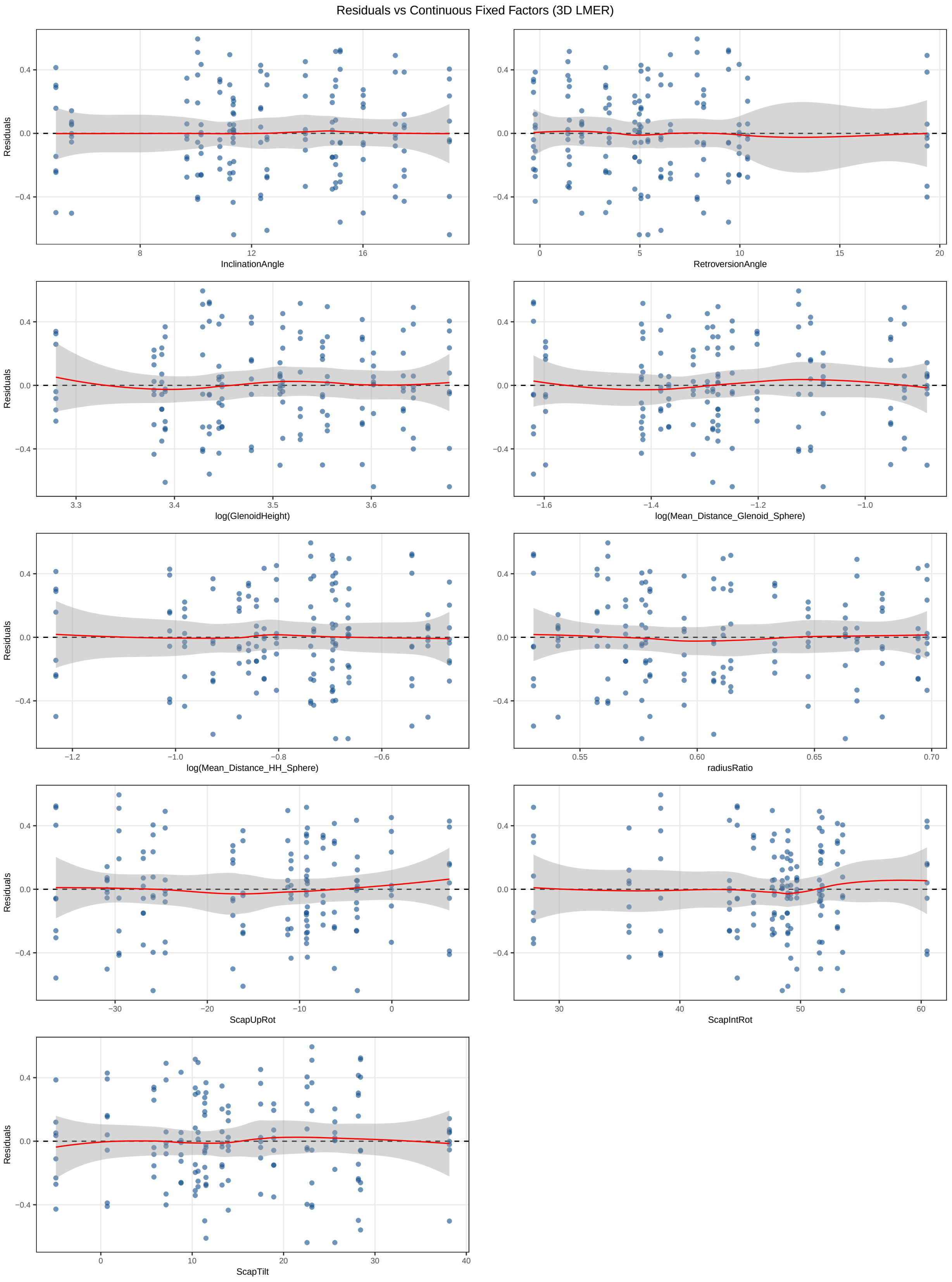

Interpretation of Residual Plots: *These plots show that there is no clear correlation between the predictor*

values and the residuals (smoothers are centered at 0), which supports our linearity assumption. The variance also does not appear to change based on predictor (meeting the homoscedasticity assumption).

Model Results and Post Hoc Analysis

Now that we confirmed that our assumptions were met, we can check the model results.

3D Translations Model Summary

```r
summary(lmer_main)
```

```
Linear mixed model fit by REML ['lmerMod']
Formula: log(max_translation) ~ InclinationAngle + RetroversionAngle +
    base_motion + weight + log(Mean_Distance_Glenoid_Sphere) +
    log(Mean_Distance_HH_Sphere) + log(GlenoidHeight) + radiusRatio +
    ScapIntRot + ScapTilt + (1 | Subject)
   Data: analysis_data_long_weight_factor

REML criterion at convergence: 122.6

Scaled residuals:
     Min       1Q   Median       3Q      Max
-2.14719 -0.65437 -0.03045  0.67452  1.99997

Random effects:
 Groups   Name        Variance Std.Dev.
 Subject  (Intercept) 0.02391  0.1546
 Residual             0.08830  0.2972
Number of obs: 150, groups:  Subject, 19

Fixed effects:
                                   Estimate Std. Error t value
(Intercept)                       -0.899914   1.902060  -0.473
InclinationAngle                   0.036815   0.015554   2.367
RetroversionAngle                 -0.001719   0.011213  -0.153
base_motionSA                     -0.057664   0.068173  -0.846
base_motionFE                      0.113116   0.068681   1.647
base_motionERaR                   -0.091773   0.089674  -1.023
base_motionERa90                  -0.198569   0.088054  -2.255
weightTRUE                         0.029126   0.055940   0.521
log(Mean_Distance_Glenoid_Sphere)  0.422449   0.274121   1.541
log(Mean_Distance_HH_Sphere)      -0.303291   0.308130  -0.984
log(GlenoidHeight)                 0.780131   0.524161   1.488
radiusRatio                       -0.776408   0.942590  -0.824
ScapIntRot                        -0.002844   0.007543  -0.377
ScapTilt                           0.010015   0.005405   1.853
```

```r
# drop1 analysis 3d translations
drop1(lmer_main, test = "Chisq")
```

```
Single term deletions

Model:
log(max_translation) ~ InclinationAngle + RetroversionAngle +
    base_motion + weight + log(Mean_Distance_Glenoid_Sphere) +
    log(Mean_Distance_HH_Sphere) + log(GlenoidHeight) + radiusRatio +
    ScapIntRot + ScapTilt + (1 | Subject)
                                 npar     AIC     LRT  Pr(Chi)
<none>                                 98.050
InclinationAngle                    1 104.517  8.4679 0.003615 **
RetroversionAngle                   1  96.090  0.0406 0.840265
base_motion                         4 104.126 14.0761 0.007056 **
weight                              1  96.337  0.2872 0.592015
log(Mean_Distance_Glenoid_Sphere)   1 100.093  4.0436 0.044339 *
log(Mean_Distance_HH_Sphere)        1  97.836  1.7860 0.181413
log(GlenoidHeight)                  1  99.882  3.8327 0.050260 .
radiusRatio                         1  97.314  1.2646 0.260788
ScapIntRot                          1  96.326  0.2769 0.598707
ScapTilt                            1 101.685  5.6354 0.017602 *
---
Signif. codes:  0 '***' 0.001 '**' 0.01 '*' 0.05 '.' 0.1 ' ' 1
```

Notes on Interpreting Effect Sizes

raw x, log y — % per +1 unit x Effect sizes of raw predictors (as percent change in outcome per + 1 unit increase in predcitor) can be derived from coefficients (β) as follows:

$\log y \sim \cdots + \beta \cdot x + \cdots$ — our model, where β is the coefficient for predictor x

$\Delta \log y = \beta \cdot \Delta x$

$\Delta x = 1$ — one unit increase in the predictor

$\Delta \log y = \beta \cdot 1$

Δy (multiplicative factor) $= \exp(\beta)$. Subtract 1 and multiply by 100 to get percent change in the **outcome**.

raw x, log y — Δ per +1 mm y

If we want the impact of the predictor on absolute translation (which is useful for evaluating the effect size relative to biplane error), then we also need a reference value (since translation outcome is log transformed in the model, the reported effects above are all relative — expressed as multiplicative factors — and therefore calculating absolute change in mm depends on what value we start with). We can calculate the Δx required to achieve a 1 mm change in translation magnitude from the mean translation $\bar{y}$ in our population. The calculations are derived as follows:

$\log y \sim \cdots + \beta \cdot x + \cdots$ — our model, where β is the coefficient for predictor x

$\Delta \log y = \log\big((\bar{y}+1)/\bar{y}\big)$ — target change in $\log y$ when y increases from $\bar{y}$ to $\bar{y}+1$

$\Delta \log y = \beta\, \Delta x$

$\Delta x = \log\big((\bar{y}+1)/\bar{y}\big)/\beta$

log x, log y — % per +1% x

Effect sizes for log-transformed predictors (as percent change in outcome per percent change in predictor) can be derived from coefficients (β) as follows:

$\log y \sim \cdots + \beta \log(x) + \cdots$ — our model, where β is the coefficient for predictor x

$\Delta \log y = \beta \cdot \Delta \log x$

$\Delta \log x = \log(1.01)$ — 1% increase in predictor

$\Delta \log y = \beta \log(1.01)$

$\Delta y = \exp(\beta \log(1.01)) = 1.01^{\beta}$ — Δy is the multiplicative factor; subtract 1 and multiply by 100 to get percent change in the **outcome**

If we want the impact of the predictor on absolute translation, as above, we again need reference values; since x is also on the log scale, we need an x reference value. We use the population mean raw predictor $\bar{x}$.

log x, log y — Δ per +1 mm y

$\log y \sim \cdots + \beta \log x + \cdots$ — our model, where β is the coefficient for predictor x

$\Delta \log y = \log\big((\bar{y}+1)/\bar{y}\big)$ — target change in $\log y$

$\Delta \log y = \beta\, \Delta \log x$

$\Delta \log x = \log\big((\bar{y}+1)/\bar{y}\big)/\beta$

$\Delta \log x = \log(x_{\text{new}}/\bar{x})$ — link $\Delta \log x$ to reference mean $\bar{x}$; with $\Delta \log x = \log\big((\bar{y}+1)/\bar{y}\big)/\beta$ this implies $\log(x_{\text{new}}/\bar{x}) = \log\big((\bar{y}+1)/\bar{y}\big)/\beta$

$x_{\text{new}}/\bar{x} = \big((\bar{y}+1)/\bar{y}\big)^{1/\beta}$ — exponentiate (equivalently $\exp(\Delta \log x)$)

$\Delta x = x_{\text{new}} - \bar{x} = \bar{x}\,\Big(\big((\bar{y}+1)/\bar{y}\big)^{1/\beta} - 1\Big)$ — raw predictor change for +1 mm y

---

Effect of Glenoid Height on 3D Translations

Glenoid height is at the significance threshold (p = 0.05).

An effect of glenoid height could be explained by a scaling factor i.e. larger glenoids will have larger absolute translations — we included glenoid height to control for this in the model.

Coefficient: $\hat{\beta} = 0.7801$ (95% CI -0.0010 to 1.5690) for `log(GlenoidHeight)`.

$\log x$**,** $\log y$ **— % per +1% x.**

Model: $\log y = \cdots + \beta \log x + \cdots$ (other terms held fixed).

Formula: $100\,(1.01^{\beta} - 1)$ with $\hat{\beta} = 0.7801$; $1.01^{\hat{\beta}} \approx 1.0078$ (95% CI 1.0000 to 1.0157).

Interpretation: 0.78% (95% CI -0.00% to 1.57%) change in maximum 3D translation per 1% increase in *glenoid height (mm).*

$\log x$**,** $\log y$ **— raw Δx for +1 mm y at $\bar{y}$.**

Model: $\log y = \cdots + \beta \log x + \cdots$ (other terms held fixed).

Reference (sample means): $\bar{y} = 4.9$ mm; $\hat{x} = 33.2\,\text{mm}$ (mean raw predictor).

Formula: $F_y = (\bar{y}+1)/\bar{y}$, $\Delta x = \hat{x}\,\big(F_y^{1/\beta} - 1\big)$; $F_y = 1.20379$, $F_y^{1/\hat{\beta}} \approx 1.26840$, $\Delta x \approx 8.9\,\text{mm}$.

Interpretation: Approximately 8.9 mm change in *glenoid height (mm)* from $\hat{x}$ corresponds to a +1 mm increase in maximum 3D translation from $\bar{y} = 4.9$ mm. *Note the large confidence interval including 0.*

We can explore some plots for height effect but note that plots do not control for other factors like the lmer does, so the direct effects from the lmer may not be matched in the plots.

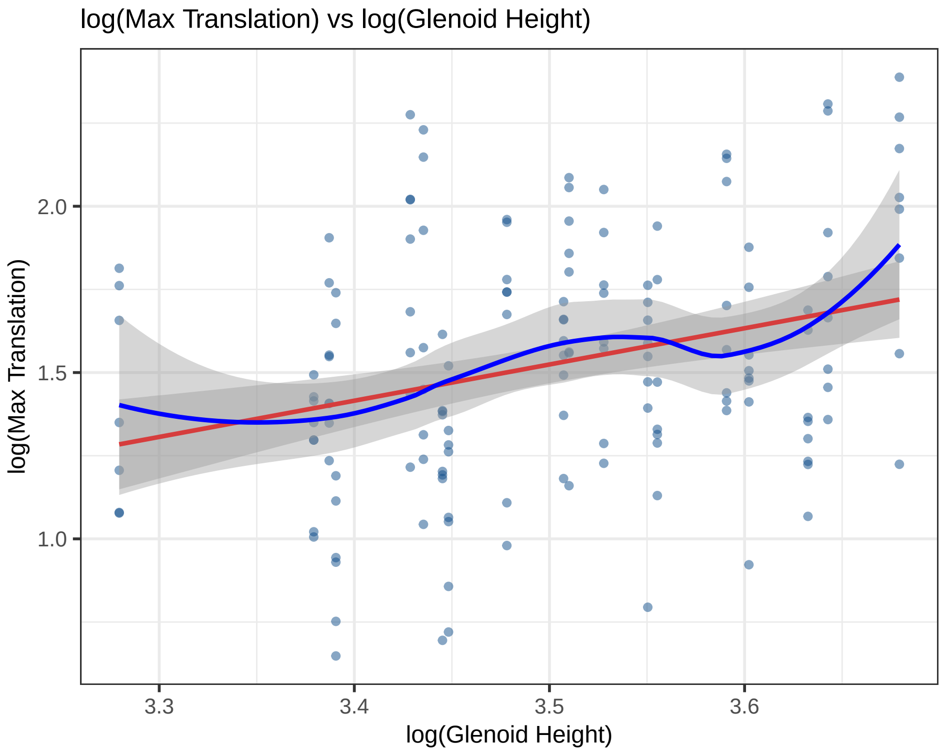

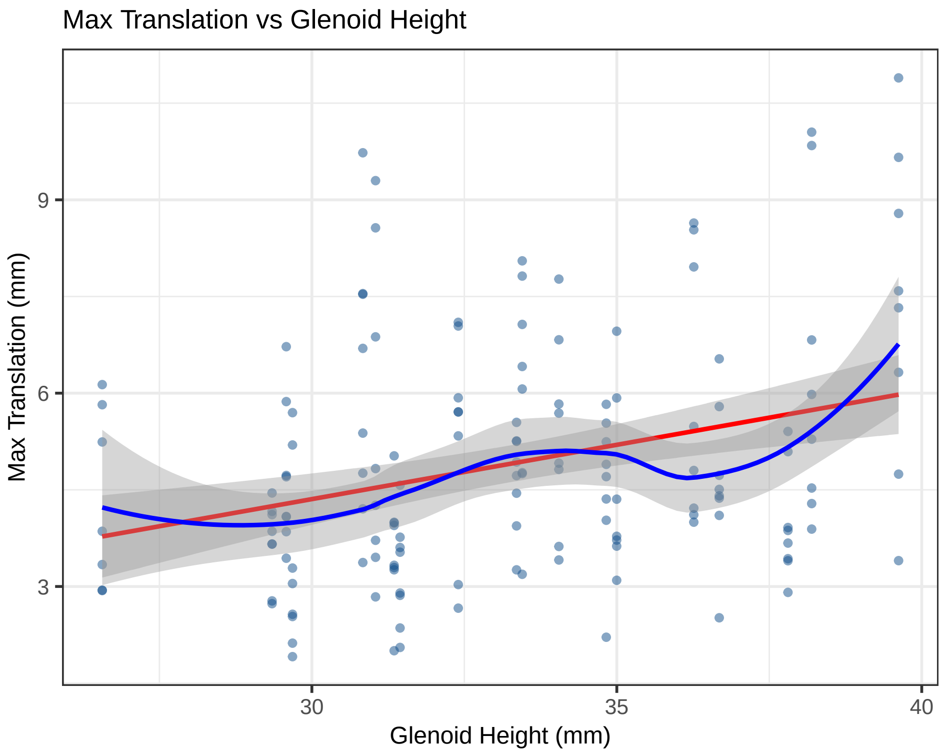

Interpretation of Glenoid Height plots: *We see that when plotting glenoid height against translation in the log and raw scale, there is a positive correlation between glenoid height and translation, as we already observed in our statistical model. The smoother (blue) is close to the linear relationship line (red).*

Is the effect of glenoid height a simple scaling factor or does glenoid height also affect max 3D translations normalized as a percent of glenoid height?

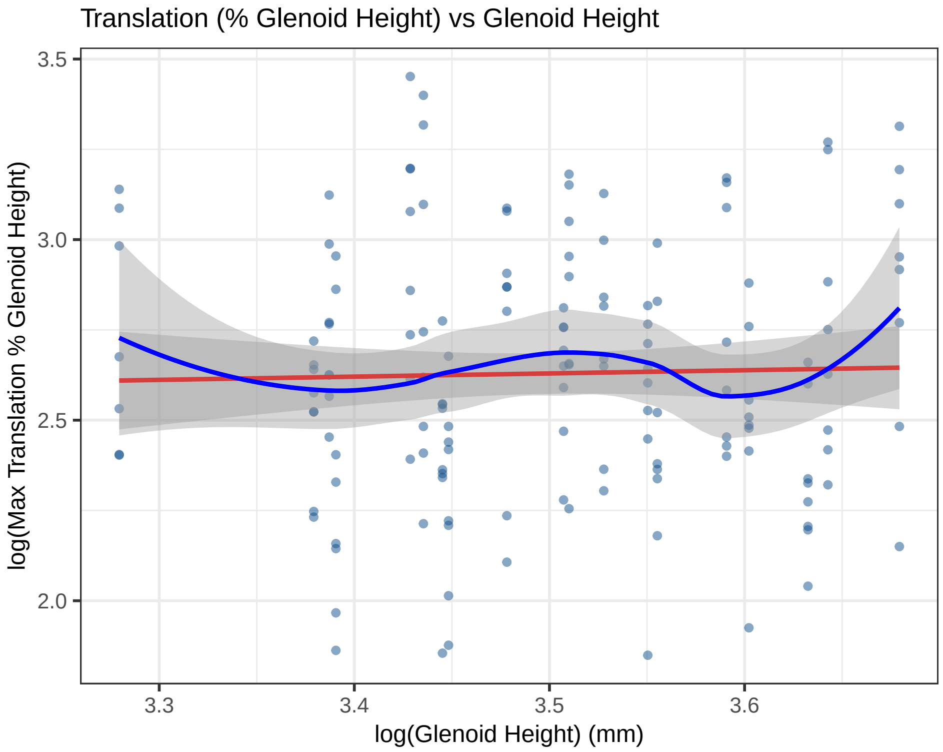


```
Linear mixed model fit by REML ['lmerMod']
Formula:
log(normalized_max_translation) ~ InclinationAngle + RetroversionAngle +
    base_motion + weight + log(Mean_Distance_Glenoid_Sphere) +
    log(Mean_Distance_HH_Sphere) + log(GlenoidHeight) + radiusRatio +
    ScapIntRot + ScapTilt + (1 | Subject)
   Data: analysis_data_long_weight_factor

REML criterion at convergence: 122.6

Scaled residuals:
     Min       1Q   Median       3Q      Max
-2.14719 -0.65437 -0.03045  0.67452  1.99997

Random effects:
 Groups   Name        Variance Std.Dev.
 Subject  (Intercept) 0.02391  0.1546
 Residual             0.08830  0.2972
Number of obs: 150, groups:  Subject, 19

Fixed effects:
                                   Estimate Std. Error t value
(Intercept)                        3.705256   1.902060   1.948
InclinationAngle                   0.036815   0.015554   2.367
RetroversionAngle                 -0.001719   0.011213  -0.153
```

```
base_motionSA                      -0.057664   0.068173  -0.846
base_motionFE                       0.113116   0.068681   1.647
base_motionERaR                    -0.091773   0.089674  -1.023
base_motionERa90                   -0.198569   0.088054  -2.255
weightTRUE                          0.029126   0.055940   0.521
log(Mean_Distance_Glenoid_Sphere)   0.422449   0.274121   1.541
log(Mean_Distance_HH_Sphere)       -0.303291   0.308130  -0.984
log(GlenoidHeight)                 -0.219869   0.524161  -0.419
radiusRatio                        -0.776408   0.942590  -0.824
ScapIntRot                         -0.002844   0.007543  -0.377
ScapTilt                            0.010015   0.005405   1.853

Single term deletions

Model:
log(normalized_max_translation) ~ InclinationAngle + RetroversionAngle +
    base_motion + weight + log(Mean_Distance_Glenoid_Sphere) +
    log(Mean_Distance_HH_Sphere) + log(GlenoidHeight) + radiusRatio +
    ScapIntRot + ScapTilt + (1 | Subject)
                                  npar     AIC     LRT  Pr(Chi)
<none>                                  98.050
InclinationAngle                     1 104.517  8.4679 0.003615 **
RetroversionAngle                    1  96.090  0.0406 0.840265
base_motion                          4 104.126 14.0761 0.007056 **
weight                               1  96.337  0.2872 0.592015
log(Mean_Distance_Glenoid_Sphere)    1 100.093  4.0436 0.044339 *
log(Mean_Distance_HH_Sphere)         1  97.836  1.7860 0.181413
log(GlenoidHeight)                   1  96.362  0.3122 0.576304
radiusRatio                          1  97.314  1.2646 0.260788
ScapIntRot                           1  96.326  0.2769 0.598707
ScapTilt                             1 101.685  5.6354 0.017602 *
---
Signif. codes:  0 '***' 0.001 '**' 0.01 '*' 0.05 '.' 0.1 ' ' 1
```

Interpretation of Glenoid Height Effect: *The glenoid height is no longer significant when investigating normalized translations in the lmer. Therefore the effect of glenoid height on absolute translations seems to be due to a scaling effect.*

Effect of Inclination Angle on 3D Translations

Coefficient: $\hat{\beta} = 0.0368$ (95% CI 0.0136 to 0.0601) for `InclinationAngle`.

Raw x, $\log y$ — % per +1 unit x.

Model: $\log y = \cdots + \beta x + \cdots$ (other terms held fixed).

Formula: $100\,(e^{\beta} - 1)$ with $\hat{\beta} = 0.0368$.

Interpretation: 3.8% (95% CI 1.4% to 6.2%) change in maximum 3D translation per degree change in *inclination angle.*

Raw x, $\log y$ — raw Δx for +1 mm y at $\bar{y}$.

Model: $\log y = \cdots + \beta x + \cdots$ (other terms held fixed).

Reference (sample means): $\bar{y} = 4.9$ mm (mean translation).

Formula: $F_y = (\bar{y}+1)/\bar{y}$, $\Delta x = \log(F_y)/\beta$; $F_y = 1.20379$, $\log(F_y) = 0.18548$, $\Delta x \approx 5.0\,\mathrm{deg}$.

Interpretation: Approximately 5.0 deg change in *inclination angle* corresponds to a +1 mm increase in maximum 3D translation from $\bar{y} = 4.9$ mm.

Effect of Mean_Distance_Glenoid_Sphere on 3D Translations

Coefficient: $\hat{\beta} = 0.4224$ (95% CI 0.0118 to 0.8314) for `log(Mean_Distance_Glenoid_Sphere)`.

$\log x$, $\log y$ — % per +1% x.

Model: $\log y = \cdots + \beta \log x + \cdots$ (other terms held fixed).

Formula: $100\,(1.01^{\beta} - 1)$ with $\hat{\beta} = 0.4224$; $1.01^{\hat{\beta}} \approx 1.0042$ (95% CI 1.0001 to 1.0083).

Interpretation: 0.42% (95% CI 0.01% to 0.83%) change in maximum 3D translation per 1% increase in *glenoid asphericity (mean distance to fitted sphere, mm).*

$\log x$, $\log y$ — raw Δx for +1 mm y at $\bar{y}$.

Model: $\log y = \cdots + \beta \log x + \cdots$ (other terms held fixed).

Reference (sample means): $\bar{y} = 4.9$ mm; $\hat{x} = 0.3\,\mathrm{mm}$ (mean raw predictor).

Formula: $F_y = (\bar{y}+1)/\bar{y}$, $\Delta x = \hat{x}\left(F_y^{1/\beta} - 1\right)$; $F_y = 1.20379$, $F_y^{1/\hat{\beta}} \approx 1.55124$, $\Delta x \approx 0.2\,\mathrm{mm}$.

Interpretation: Approximately 0.2 mm change in *glenoid asphericity (mean distance to fitted sphere, mm)* from $\hat{x}$ corresponds to a +1 mm increase in maximum 3D translation from $\bar{y} = 4.9$ mm.

Effect of Scapular Tilt on 3D Translations

Coefficient: $\hat{\beta} = 0.0100$ (95% CI 0.0020 to 0.0181) for `ScapTilt`.

Raw x, $\log y$ — % per +1 unit x.

Model: $\log y = \cdots + \beta x + \cdots$ (other terms held fixed).

Formula: $100\,(\mathrm{e}^{\beta} - 1)$ with $\hat{\beta} = 0.0100$.

Interpretation: 1.0% (95% CI 0.2% to 1.8%) change in maximum 3D translation per degree change in *scapular tilt.*

Raw x, $\log y$ — raw Δx for +1 mm y at $\bar{y}$.

Model: $\log y = \cdots + \beta x + \cdots$ (other terms held fixed).

Reference (sample means): $\bar{y} = 4.9$ mm (mean translation).

Formula: $F_y = (\bar{y}+1)/\bar{y}$, $\Delta x = \log(F_y)/\beta$; $F_y = 1.20379$, $\log(F_y) = 0.18548$, $\Delta x \approx 18.5\,\mathrm{deg}$.

Interpretation: Approximately 18.5 deg change in *scapular tilt* corresponds to a +1 mm increase in maximum 3D translation from $\bar{y} = 4.9$ mm.

Post Hoc Comparisons for 3D Motion

Motion also had a significant effect on translations. Let's investigate this further. Note that weighted and unweighted motions are both included in the effect of that motion, but that our lmer model controls for addition of weight by including weight as a fixed factor.

```r
# Function to perform post-hoc analysis for a given model
perform_posthoc_analysis <- function(model, model_name) {


  # Perform pairwise comparisons for all motion levels using Tukey method
  pairwise_contrasts <- glht(model, linfct = mcp(base_motion = "Tukey"))

  # Summary of pairwise comparisons
  summary_motion_contrasts <- summary(pairwise_contrasts)


  # Check the summary of pairwise comparisons
  print(summary_motion_contrasts)

  # Get confidence intervals
  ci_results <- confint(pairwise_contrasts)

  # Find significant pairs directly
  significant_indices <- which(summary_motion_contrasts$test$pvalues <= 0.05)

  # Print significant comparisons
  if (length(significant_indices) > 0) {
    cat("Significant motion differences:\n")
    for (i in significant_indices) {
      comparison_name <- names(summary_motion_contrasts$test$coefficients)[i]
      cat(comparison_name, "p =",
       ↪  format_p_value(summary_motion_contrasts$test$pvalues[i]), "\n")
    }
  } else {
    cat("No significant motion differences found.\n")
  }

  # Loop through each significant pair for interpretations
  for (idx in significant_indices) {
    # Get values for this comparison directly from the original objects
    comparison <- names(summary_motion_contrasts$test$coefficients)[idx]
    estimate <- summary_motion_contrasts$test$coefficients[idx]
    lower_ci <- ci_results$confint[idx, "lwr"]
    upper_ci <- ci_results$confint[idx, "upr"]
    p_val <- summary_motion_contrasts$test$pvalues[idx]

    # Calculate exponentiated values (since output is log transformed)
    exp_est <- exp(estimate)
    exp_lower <- exp(lower_ci)
    exp_upper <- exp(upper_ci)

    # for percent change, need to use -1
    percent_change <- (exp_est - 1) * 100
    percent_ci_lower <- (exp_lower - 1) * 100
    percent_ci_upper <- (exp_upper - 1) * 100

    motion_parts <- strsplit(comparison, " - ")[[1]]

    if (percent_change > 0) {
```

```
    cat(sprintf("%s has %.1f%% higher translations than %s (95%% CI: %.1f%% to %.1f%%,
    ↪  p=%s)\n\n",
                 motion_parts[1], abs(percent_change), motion_parts[2],
↪  percent_ci_lower, percent_ci_upper, format_p_value(p_val)))
   } else {
     cat(sprintf("%s has %.1f%% lower translations than %s (95%% CI: %.1f%% to %.1f%%,
     ↪  p=%s)\n\n",
                 motion_parts[1], abs(percent_change), motion_parts[2],
↪  abs(percent_ci_upper), abs(percent_ci_lower), format_p_value(p_val)))
   }
  }

  cat("\n")
  return(list(pairwise_contrasts = pairwise_contrasts,
              summary_motion_contrasts = summary_motion_contrasts,
              significant_indices = significant_indices))
}

# Perform post-hoc analysis
posthoc_results_3D <- perform_posthoc_analysis(lmer_main, "3D Translations")
```

```
     Simultaneous Tests for General Linear Hypotheses

Multiple Comparisons of Means: Tukey Contrasts


Fit: lmer(formula = log(max_translation) ~ InclinationAngle + RetroversionAngle +
    base_motion + weight + log(Mean_Distance_Glenoid_Sphere) +
    log(Mean_Distance_HH_Sphere) + log(GlenoidHeight) + radiusRatio +
    ScapIntRot + ScapTilt + (1 | Subject), data = analysis_data_long_weight_factor,
    na.action = na.exclude)

Linear Hypotheses:
                  Estimate Std. Error z value Pr(>|z|)
SA - CA == 0      -0.05766    0.06817  -0.846  0.91444
FE - CA == 0       0.11312    0.06868   1.647  0.46155
ERaR - CA == 0    -0.09177    0.08967  -1.023  0.84171
ERa90 - CA == 0   -0.19857    0.08805  -2.255  0.15623
FE - SA == 0       0.17078    0.06868   2.487  0.09133 .
ERaR - SA == 0    -0.03411    0.08967  -0.380  0.99545
ERa90 - SA == 0   -0.14091    0.08805  -1.600  0.49155
ERaR - FE == 0    -0.20489    0.09033  -2.268  0.15180
ERa90 - FE == 0   -0.31169    0.08871  -3.514  0.00396 **
ERa90 - ERaR == 0 -0.10680    0.09789  -1.091  0.80788
---
Signif. codes:  0 '***' 0.001 '**' 0.01 '*' 0.05 '.' 0.1 ' ' 1
(Adjusted p values reported -- single-step method)

Significant motion differences:
ERa90 - FE p = 0.004
ERa90 has 26.8% lower translations than FE (95% CI: 6.8% to 42.5%, p=0.004)
```

```r
# Store results for plotting
pairwise_contrasts <- posthoc_results_3D$pairwise_contrasts
summary_motion_contrasts <- posthoc_results_3D$summary_motion_contrasts
significant_indices <- posthoc_results_3D$significant_indices
```

Interpretation Motion Effects: *The post-hoc analysis shows significant pairwise differences between some motions. This is also shown graphically in Appendix A (Supplementary Plots) in the publication.*

SI Model Analysis (Y Translation)

Now let's fit and analyze the SI (Y translation) model first.

Histogram Plots for SI Model

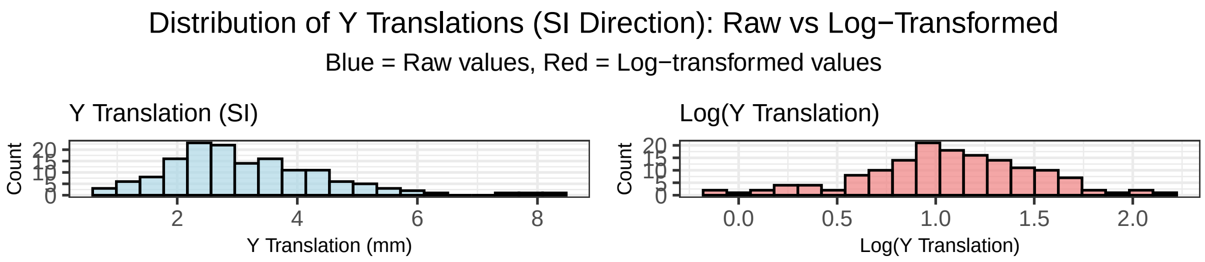

SI Translations (Y) vs Fixed Factors

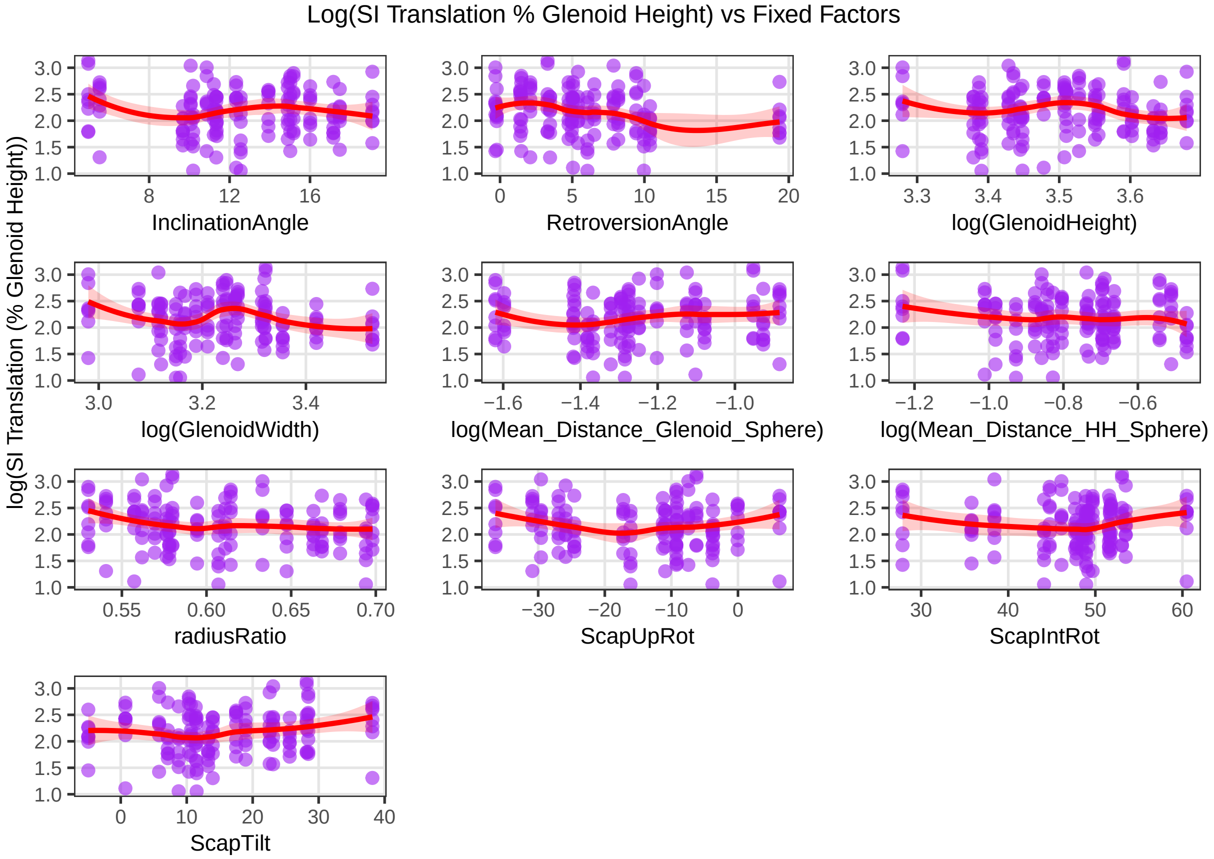

VIF Analysis for SI Model

```
# Check for multicollinearity among predictors for Y translation (SI direction) model
lmer_for_vif_Y = lmer(log(Y_translation) ~ InclinationAngle + RetroversionAngle +
↪   base_motion + weight + log(Mean_Distance_Glenoid_Sphere) +
↪   log(Mean_Distance_HH_Sphere) + log(GlenoidHeight) + log(GlenoidWidth) + radiusRatio +
↪   ScapUpRot + ScapIntRot + ScapTilt + (1 | Subject), na.action = na.exclude, data =
↪   analysis_data_long_weight_factor)

# Calculate VIF values
vif_values_Y <- vif(lmer_for_vif_Y)
print("VIF values for Y translation (SI direction) model:")
```

```
[1] "VIF values for Y translation (SI direction) model:"
```

```
print(vif_values_Y)
```

```
                                      GVIF Df GVIF^(1/(2*Df))
InclinationAngle                  3.771538  1        1.942045
```

```
RetroversionAngle                 3.231844  1        1.797733
base_motion                       1.253108  4        1.028605
weight                            1.251587  1        1.118744
log(Mean_Distance_Glenoid_Sphere) 2.003610  1        1.415489
log(Mean_Distance_HH_Sphere)      1.837651  1        1.355600
log(GlenoidHeight)                5.085619  1        2.255132
log(GlenoidWidth)                 6.831607  1        2.613734
radiusRatio                       2.036747  1        1.427146
ScapUpRot                         6.383368  1        2.526533
ScapIntRot                        1.829888  1        1.352733
ScapTilt                          5.489073  1        2.342877
```

We already know from the 3D analysis that width and height are correlated, and scap up rot and tilt are correlated. VIF analysis again shows that VIF values above 5 so we remove glenoid width and scap up rot.

```
# rerun vif
lmer_for_vif_Y = lmer(log(Y_translation) ~ InclinationAngle + RetroversionAngle +
↪  base_motion + weight + log(Mean_Distance_Glenoid_Sphere) +
↪  log(Mean_Distance_HH_Sphere) + log(GlenoidHeight) + radiusRatio + ScapIntRot +
↪  ScapTilt + (1 | Subject), na.action = na.exclude, data =
↪  analysis_data_long_weight_factor)

# Calculate VIF values
vif_values_Y <- vif(lmer_for_vif_Y)
print("New VIF values for Y translation (SI direction) model:")
```

```
[1] "New VIF values for Y translation (SI direction) model:"
```

```
print(vif_values_Y)
```

```
                                      GVIF Df GVIF^(1/(2*Df))
InclinationAngle                  1.765576  1        1.328750
RetroversionAngle                 1.381362  1        1.175314
base_motion                       1.253122  4        1.028606
weight                            1.251515  1        1.118711
log(Mean_Distance_Glenoid_Sphere) 1.614283  1        1.270544
log(Mean_Distance_HH_Sphere)      1.689610  1        1.299850
log(GlenoidHeight)                1.542638  1        1.242030
radiusRatio                       1.244519  1        1.115580
ScapIntRot                        1.508282  1        1.228121
ScapTilt                          1.669722  1        1.292177
```

Great, our VIF values are all below 5 now.

Fit LMER Model for SI Translations (removing glenoid width and scap up rot because of correlations between predictors)

```
# Y translation (SI direction)
lmer_main_Y = lmer(log(Y_translation) ~ InclinationAngle + RetroversionAngle +
↪  base_motion + weight + log(Mean_Distance_Glenoid_Sphere) +
↪  log(Mean_Distance_HH_Sphere) + log(GlenoidHeight) + radiusRatio + ScapIntRot +
↪  ScapTilt + (1 | Subject), na.action = na.exclude, data =
↪  analysis_data_long_weight_factor)
```

```r
# Save residuals for SI model
analysis_data_long_weight_factor$residuals_SI <- resid(lmer_main_Y)
```

Model Assumption Checks for SI Model

Now let's examine the model assumptions for the SI model using the same diagnostic plots we used for the 3D model.

QQ Plot for SI Model

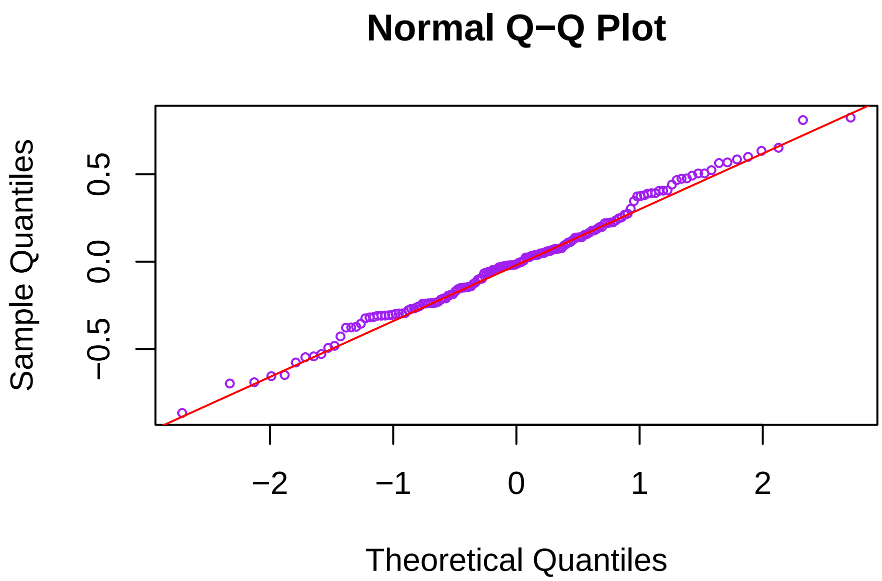

Interpretation Q-Q Plot for SI Model: *The residuals for the SI model appear to be normally distributed.*

Tukey-Anscombe Plot for SI Model

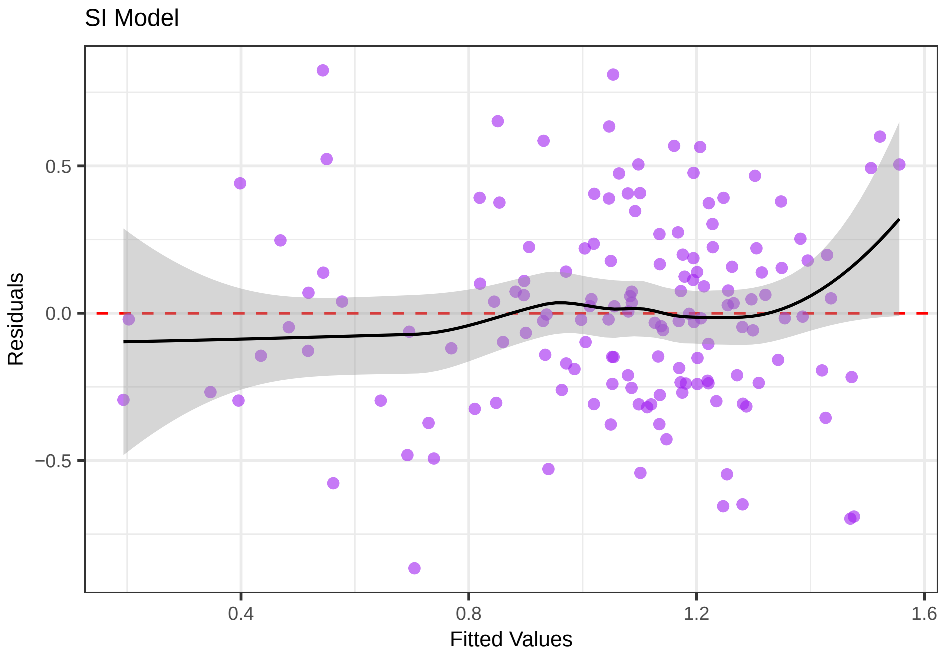

Interpretation of Tukey-Anscombe Plot for SI Model: *The smoother here appears centered on 0 across fitted values, supporting the linear model (if the linear model was not an adequate fit, the residuals would change across fitted values). The decrease and increase in the smoother at the lowest and highest fitted values in the plot are likely due to the scarcity of the data in these regions (only 1-2 observations, which affect the smoother). The data also looks homoscedastic (equal variance of residuals across fitted values) in this plot.*

Scale-Location Plot for SI Model

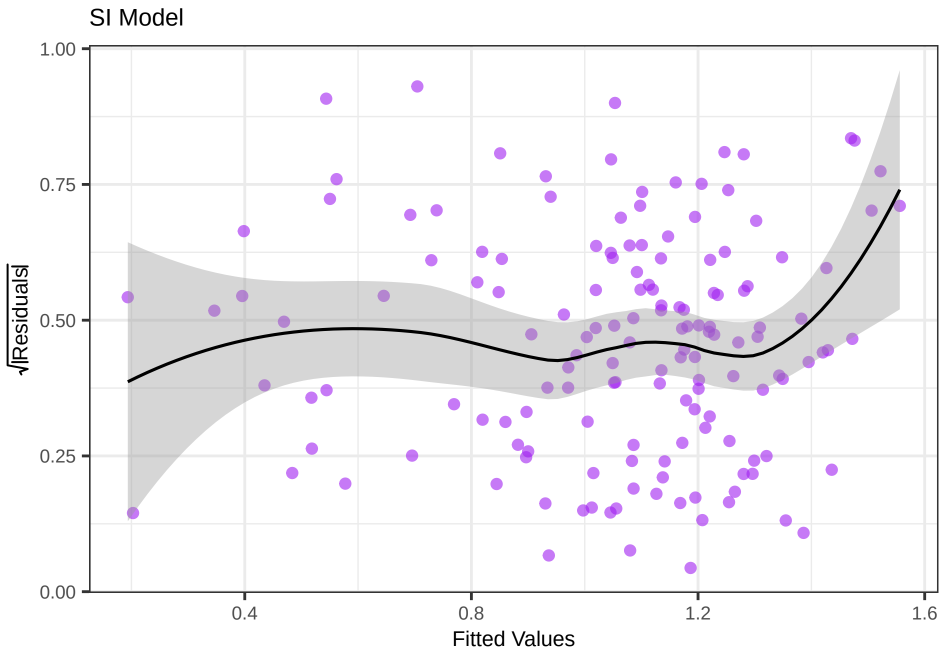

Interpretation of Scale-Location Plot for SI Model: *The variance of the residuals appears to be fairly constant across fitted values for the SI model, confirming homoscedasticity. Again, the changes at the ends of*

the plots are likely due to the low sample size in these regions/natural randomness of the data (the uncertainty of the smoothers in these regions also increases due to low number of observations).

Residuals vs Predictors for SI Model

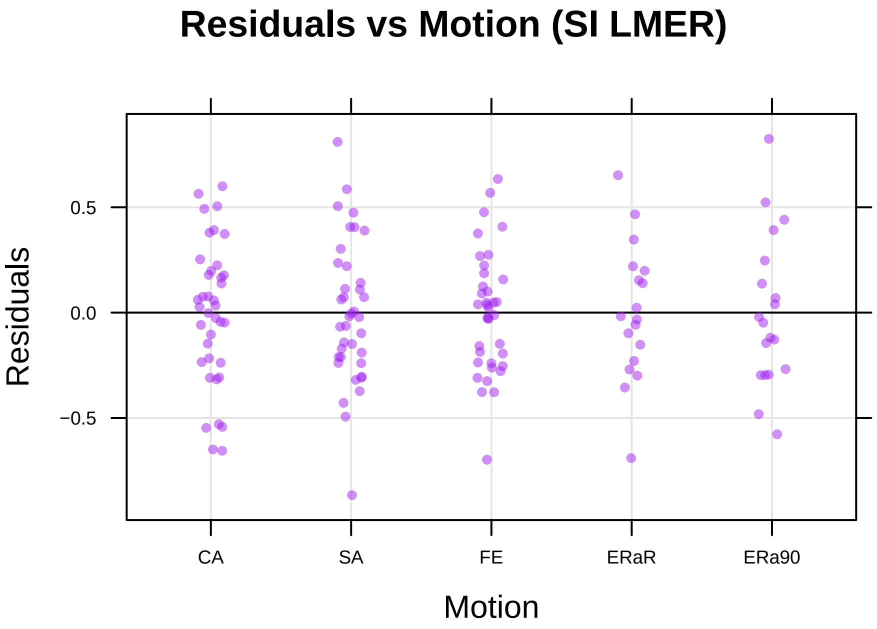

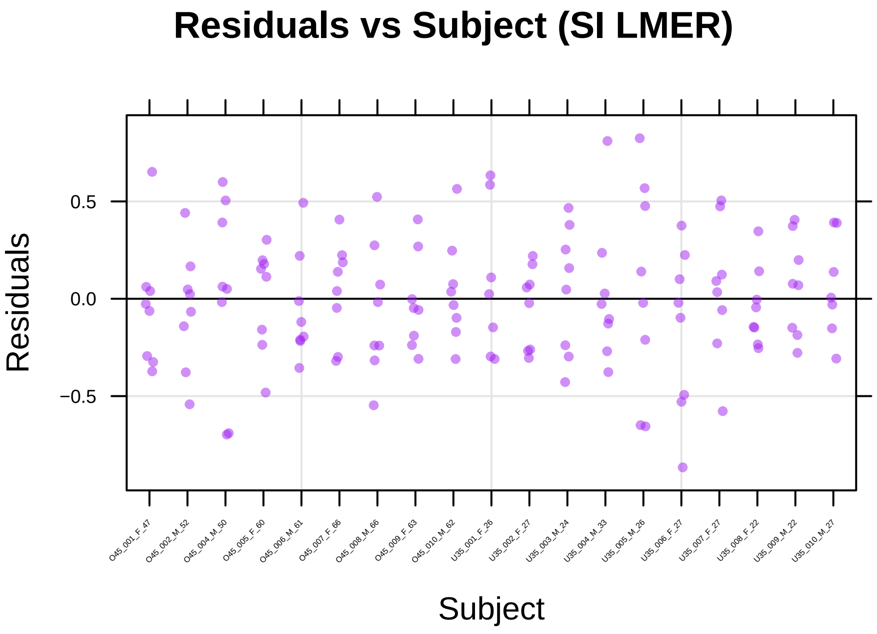

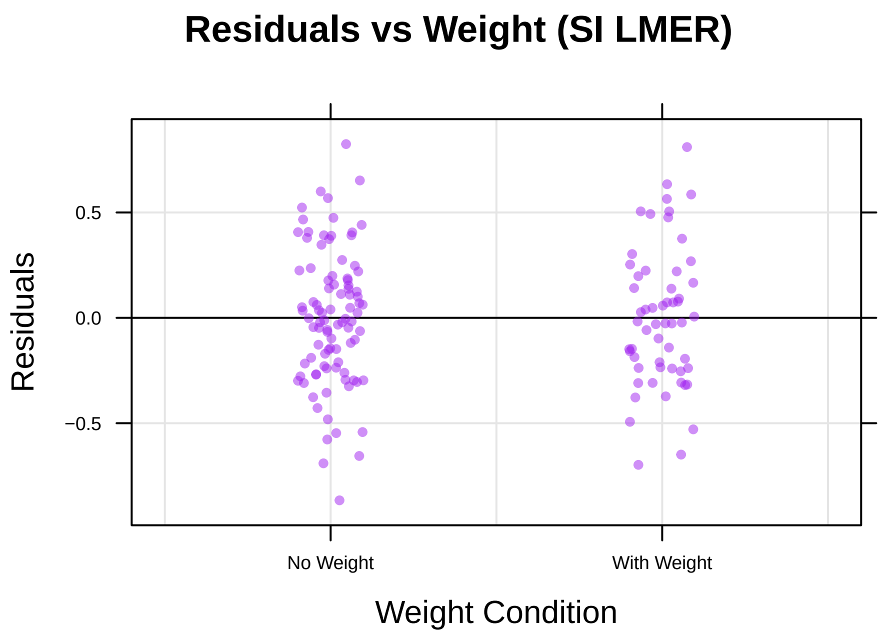
Residuals vs Weight (SI LMER)
Residuals
0.5
0.0
−0.5
No Weight
With Weight
Weight Condition

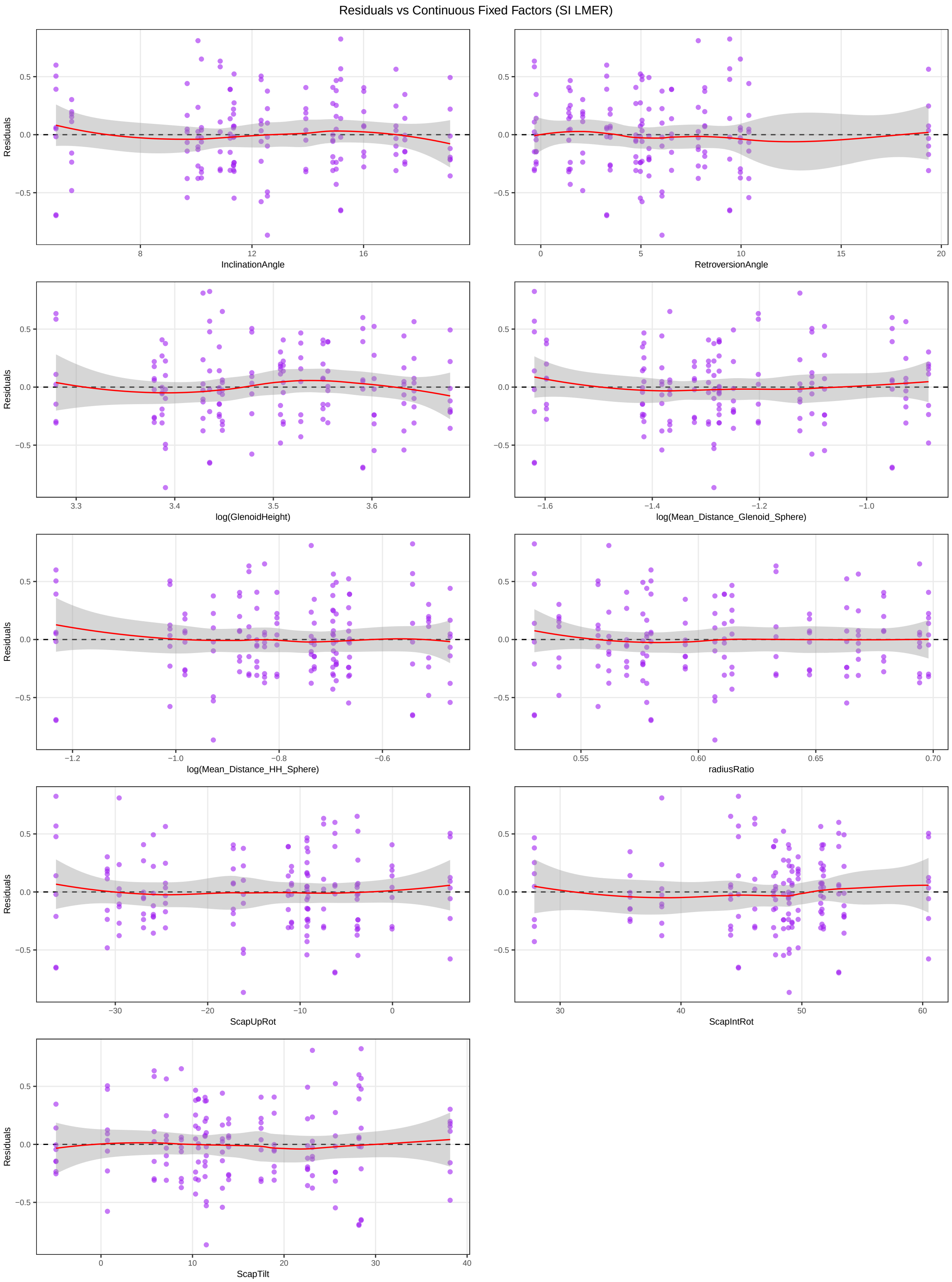

Interpretation of Residual Plots for SI Model: *These plots show that there is no clear correlation*

between the predictor values and the residuals (in other words, the smoothers are centered at 0), which supports our linearity assumption. There is also no clear correlation between predictor and variance (meeting the homoscedasticity assumption).

Model Results for SI Model

```r
summary(lmer_main_Y)
```

```
Linear mixed model fit by REML ['lmerMod']
Formula:
log(Y_translation) ~ InclinationAngle + RetroversionAngle + base_motion +
    weight + log(Mean_Distance_Glenoid_Sphere) + log(Mean_Distance_HH_Sphere) +
    log(GlenoidHeight) + radiusRatio + ScapIntRot + ScapTilt +
    (1 | Subject)
   Data: analysis_data_long_weight_factor

REML criterion at convergence: 157.7

Scaled residuals:
     Min       1Q   Median       3Q      Max
-2.52761 -0.68928 -0.04221  0.56932  2.40629

Random effects:
 Groups   Name        Variance Std.Dev.
 Subject  (Intercept) 0.01762  0.1327
 Residual             0.11736  0.3426
Number of obs: 150, groups:  Subject, 19

Fixed effects:
                                   Estimate Std. Error t value
(Intercept)                       -1.857720   1.831635  -1.014
InclinationAngle                   0.012718   0.014959   0.850
RetroversionAngle                 -0.015497   0.010782  -1.437
base_motionSA                     -0.201210   0.078593  -2.560
base_motionFE                     -0.086051   0.079169  -1.087
base_motionERaR                   -0.045719   0.103358  -0.442
base_motionERa90                  -0.702966   0.101512  -6.925
weightTRUE                         0.034031   0.064485   0.528
log(Mean_Distance_Glenoid_Sphere)  0.150328   0.263585   0.570
log(Mean_Distance_HH_Sphere)      -0.222984   0.296393  -0.752
log(GlenoidHeight)                 1.022880   0.504783   2.026
radiusRatio                       -0.940078   0.906192  -1.037
ScapIntRot                        -0.001792   0.007251  -0.247
ScapTilt                           0.006120   0.005199   1.177
```

```r
drop1(lmer_main_Y, test = "Chisq")
```

```
Single term deletions

Model:
log(Y_translation) ~ InclinationAngle + RetroversionAngle + base_motion +
```

```
    weight + log(Mean_Distance_Glenoid_Sphere) + log(Mean_Distance_HH_Sphere) +
    log(GlenoidHeight) + radiusRatio + ScapIntRot + ScapTilt +
    (1 | Subject)
                                 npar    AIC    LRT   Pr(Chi)
<none>                                 133.82
InclinationAngle                    1 133.18  1.362   0.24319
RetroversionAngle                   1 135.38  3.558   0.05924 .
base_motion                         4 174.18 48.358 7.948e-10 ***
weight                              1 132.13  0.304   0.58131
log(Mean_Distance_Glenoid_Sphere)   1 132.42  0.602   0.43778
log(Mean_Distance_HH_Sphere)        1 132.92  1.095   0.29541
log(GlenoidHeight)                  1 138.41  6.584   0.01029 *
radiusRatio                         1 133.80  1.978   0.15958
ScapIntRot                          1 131.95  0.126   0.72256
ScapTilt                            1 134.35  2.529   0.11178
---
Signif. codes:  0 '***' 0.001 '**' 0.01 '*' 0.05 '.' 0.1 ' ' 1
```

Effect of Glenoid Height on SI Translations

Glenoid height is significant for SI translations.

This could be explained by a scaling factor (i.e. larger glenoids will have larger absolute translations) — we included glenoid height to control for this in the model. Below we quantify the effect of glenoid height on SI translations.

Coefficient: $\hat{\beta} = 1.0229$ (95% CI 0.2727 to 1.7823) for `log(GlenoidHeight)`.

$\log x$, $\log y$ — % per +1% x.

Model: $\log y = \cdots + \beta \log x + \cdots$ (other terms held fixed).

Formula: $100\,(1.01^{\beta} - 1)$ with $\hat{\beta} = 1.0229$; $1.01^{\hat{\beta}} \approx 1.0102$ (95% CI 1.0027 to 1.0179).

Interpretation: 1.02% (95% CI 0.27% to 1.79%) change in SI translation per 1% increase in *glenoid height (mm)*.

$\log x$, $\log y$ — raw Δx for +1 mm y at $\bar{y}$.

Model: $\log y = \cdots + \beta \log x + \cdots$ (other terms held fixed).

Reference (sample means): $\bar{y} = 3.1$ mm; $\hat{x} = 33.2\,\text{mm}$ (mean raw predictor).

Formula: $F_y = (\bar{y} + 1)/\bar{y}$, $\Delta x = \hat{x}\left(F_y^{1/\beta} - 1\right)$; $F_y = 1.31749$, $F_y^{1/\hat{\beta}} \approx 1.30939$, $\Delta x \approx 10.3\,\text{mm}$.

Interpretation: Approximately 10.3 mm change in *glenoid height (mm)* from $\hat{x}$ corresponds to a +1 mm increase in SI translation from $\bar{y} = 3.1$ mm.

We can explore some plots for height effect but note that plots do not control for other factors like the lmer does, so the direct effects from the lmer may not be matched in the plots.

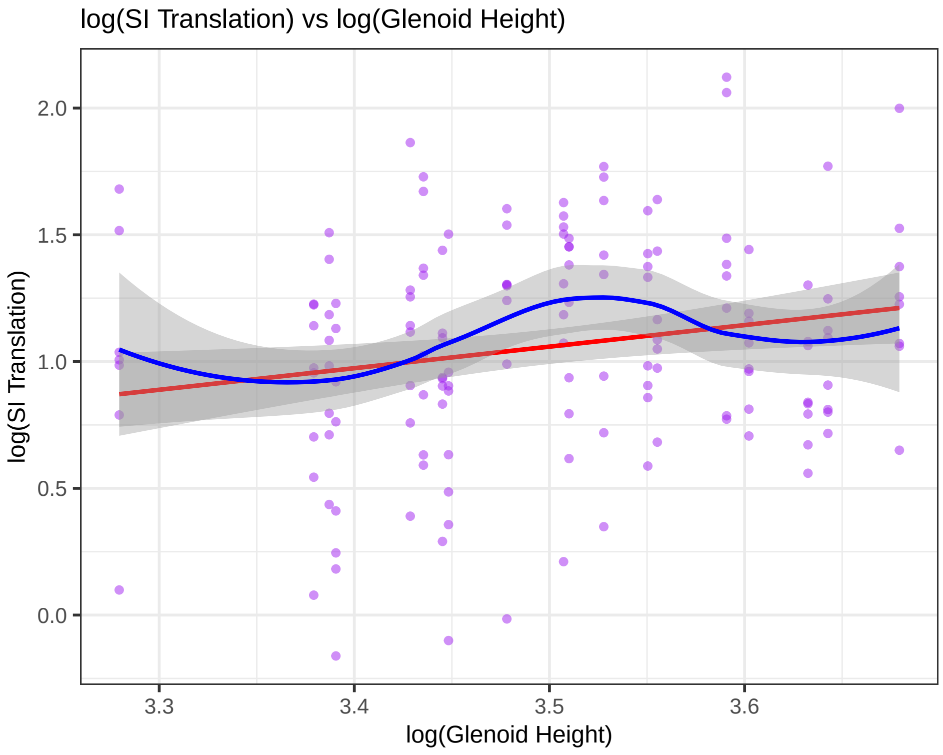

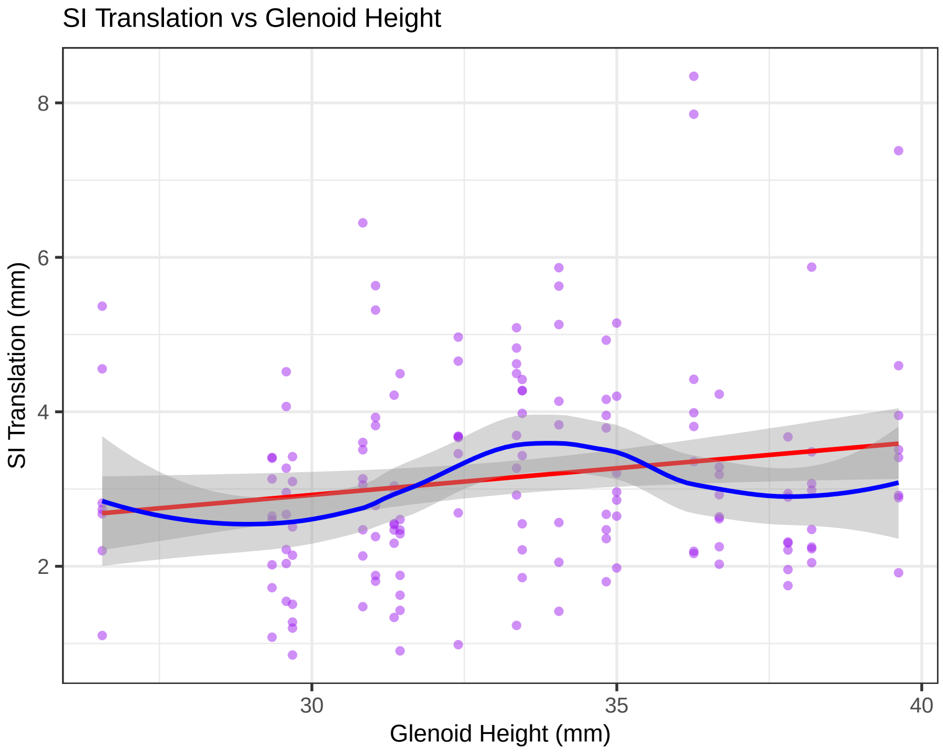

Interpretation of Glenoid Height plots for SI Model: *We see that when plotting glenoid height against SI translation in the log and raw scale, there is a positive correlation between glenoid height and SI translation, as we already observed in our statistical model. The smoother (blue) is close to the linear relationship line (red).*

Is the effect of glenoid height a simple scaling factor or does glenoid height also affect SI translations normalized as a percent of glenoid height?

```
plot_height_normalized_translation_si <- ggplot(analysis_data_long_weight_factor, aes(x =
↪  log(GlenoidHeight), y = log(normalized_Y_translation))) +
  geom_point(alpha = 0.5, color = "purple", size = 1) +
  geom_smooth(method = "lm", color = "red", se = TRUE, linewidth = 0.8) +
  geom_smooth(method = "loess", method.args = list(family = "symmetric"), color = "blue",
  ↪  se = TRUE, linewidth = 0.8) +
  labs(title = "SI Translation (% Glenoid Height) vs Glenoid Height",
       x = "log(Glenoid Height)",
       y = "log(SI Translation % Glenoid Height)") +
  theme_bw() +
  theme(
    plot.title = element_text(size = 10),
    axis.title = element_text(size = 9),
    axis.text = element_text(size = 8)
  )

print(plot_height_normalized_translation_si)
```

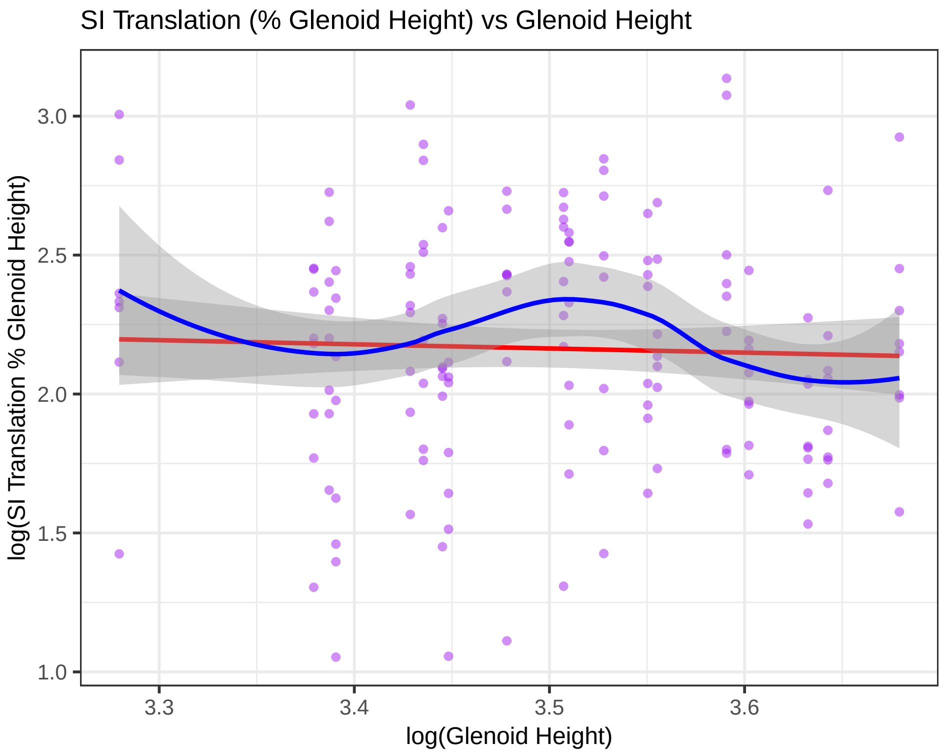


```
# as a check, also make a new lmer with normalized_Y_translation as the output
lmer_main_normalized_si_translations = lmer(log(normalized_Y_translation) ~
↪  InclinationAngle + RetroversionAngle + base_motion + weight +
↪  log(Mean_Distance_Glenoid_Sphere) + log(Mean_Distance_HH_Sphere) + log(GlenoidHeight)
↪  + radiusRatio + ScapIntRot + ScapTilt + (1 | Subject), na.action = na.exclude, data =
↪  analysis_data_long_weight_factor)
```

```r
summary(lmer_main_normalized_si_translations)
```

```
Linear mixed model fit by REML ['lmerMod']
Formula:
log(normalized_Y_translation) ~ InclinationAngle + RetroversionAngle +
    base_motion + weight + log(Mean_Distance_Glenoid_Sphere) +
    log(Mean_Distance_HH_Sphere) + log(GlenoidHeight) + radiusRatio +
    ScapIntRot + ScapTilt + (1 | Subject)
   Data: analysis_data_long_weight_factor

REML criterion at convergence: 157.7

Scaled residuals:
     Min       1Q   Median       3Q      Max
-2.52761 -0.68928 -0.04221  0.56932  2.40629

Random effects:
 Groups   Name        Variance Std.Dev.
 Subject  (Intercept) 0.01762  0.1327
 Residual             0.11736  0.3426
Number of obs: 150, groups:  Subject, 19

Fixed effects:
                                   Estimate Std. Error t value
(Intercept)                        2.747450   1.831635   1.500
InclinationAngle                   0.012718   0.014959   0.850
RetroversionAngle                 -0.015497   0.010782  -1.437
base_motionSA                     -0.201210   0.078593  -2.560
base_motionFE                     -0.086051   0.079169  -1.087
base_motionERaR                   -0.045719   0.103358  -0.442
base_motionERa90                  -0.702966   0.101512  -6.925
weightTRUE                         0.034031   0.064485   0.528
log(Mean_Distance_Glenoid_Sphere)  0.150328   0.263585   0.570
log(Mean_Distance_HH_Sphere)      -0.222984   0.296393  -0.752
log(GlenoidHeight)                 0.022880   0.504783   0.045
radiusRatio                       -0.940078   0.906192  -1.037
ScapIntRot                        -0.001792   0.007251  -0.247
ScapTilt                           0.006120   0.005199   1.177
```

```r
drop1(lmer_main_normalized_si_translations, test = "Chisq")
```

```
Single term deletions

Model:
log(normalized_Y_translation) ~ InclinationAngle + RetroversionAngle +
    base_motion + weight + log(Mean_Distance_Glenoid_Sphere) +
    log(Mean_Distance_HH_Sphere) + log(GlenoidHeight) + radiusRatio +
    ScapIntRot + ScapTilt + (1 | Subject)
                                  npar    AIC    LRT   Pr(Chi)
<none>                                 133.82
InclinationAngle                     1 133.18  1.362   0.24319
RetroversionAngle                    1 135.38  3.558   0.05924 .
```

```
base_motion                        4 174.18 48.358 7.948e-10 ***
weight                             1 132.13  0.304   0.58131
log(Mean_Distance_Glenoid_Sphere)  1 132.42  0.602   0.43778
log(Mean_Distance_HH_Sphere)       1 132.92  1.095   0.29541
log(GlenoidHeight)                 1 131.83  0.007   0.93353
radiusRatio                        1 133.80  1.978   0.15958
ScapIntRot                         1 131.95  0.126   0.72256
ScapTilt                           1 134.35  2.529   0.11178
---
Signif. codes:  0 '***' 0.001 '**' 0.01 '*' 0.05 '.' 0.1 ' ' 1
```

Interpretation of Glenoid Height Effect for SI Model: *The glenoid height is no longer significant when investigating normalized SI translations in the lmer. Therefore the effect of glenoid height on absolute SI translations seems to be due to a scaling effect.*

Post Hoc Comparisons for SI Model

Motion also had a significant effect on translations. Let's investigate this further for all three models: Note that weighted and unweighted motions are both included in the effect of that motion, but that our lmer model controls for addition of weight by including weight as a fixed factor.

```
# Perform post-hoc analysis for SI model
posthoc_results_SI <- perform_posthoc_analysis(lmer_main_Y, "Y Translation (SI
↪  Direction)")
```

```
     Simultaneous Tests for General Linear Hypotheses

Multiple Comparisons of Means: Tukey Contrasts


Fit: lmer(formula = log(Y_translation) ~ InclinationAngle + RetroversionAngle +
    base_motion + weight + log(Mean_Distance_Glenoid_Sphere) +
    log(Mean_Distance_HH_Sphere) + log(GlenoidHeight) + radiusRatio +
    ScapIntRot + ScapTilt + (1 | Subject), data = analysis_data_long_weight_factor,
    na.action = na.exclude)

Linear Hypotheses:
                  Estimate Std. Error z value Pr(>|z|)
SA - CA == 0      -0.20121    0.07859  -2.560   0.0759 .
FE - CA == 0      -0.08605    0.07917  -1.087   0.8100
ERaR - CA == 0    -0.04572    0.10336  -0.442   0.9919
ERa90 - CA == 0   -0.70297    0.10151  -6.925   <1e-04 ***
FE - SA == 0       0.11516    0.07917   1.455   0.5868
ERaR - SA == 0     0.15549    0.10336   1.504   0.5541
ERa90 - SA == 0   -0.50176    0.10151  -4.943   <1e-04 ***
ERaR - FE == 0     0.04033    0.10410   0.387   0.9951
ERa90 - FE == 0   -0.61691    0.10226  -6.033   <1e-04 ***
ERa90 - ERaR == 0 -0.65725    0.11283  -5.825   <1e-04 ***
---
Signif. codes:  0 '***' 0.001 '**' 0.01 '*' 0.05 '.' 0.1 ' ' 1
(Adjusted p values reported -- single-step method)
```

```
Significant motion differences:
ERa90 - CA p = <0.001
ERa90 - SA p = <0.001
ERa90 - FE p = <0.001
ERa90 - ERaR p = <0.001
ERa90 has 50.5% lower translations than CA (95% CI: 34.8% to 62.4%, p=<0.001)

ERa90 has 39.5% lower translations than SA (95% CI: 20.2% to 54.1%, p=<0.001)

ERa90 has 46.0% lower translations than FE (95% CI: 28.7% to 59.1%, p=<0.001)

ERa90 has 48.2% lower translations than ERaR (95% CI: 29.6% to 61.9%, p=<0.001)
```

Interpretation Motion Effects: *The post-hoc analysis shows significant pairwise differences between some motions. This is also shown graphically in Appendix A (Supplementary Plots) in the publication.*

AP Model Analysis (X Translation)

Now let's fit and analyze the AP (X translation) model.

Histogram Plots for AP Model

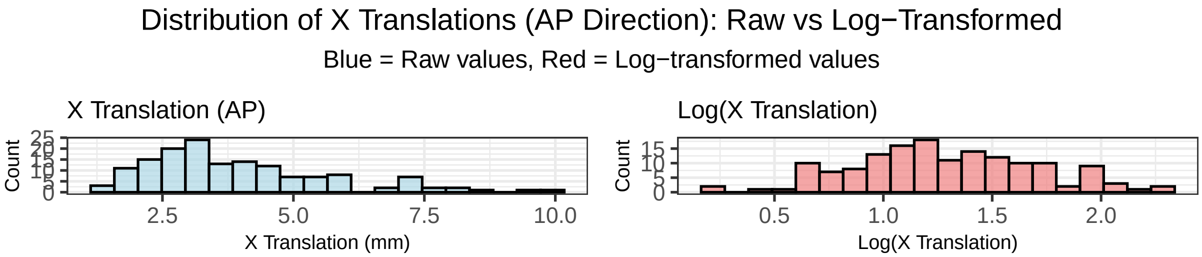

AP Translations vs Fixed Factors

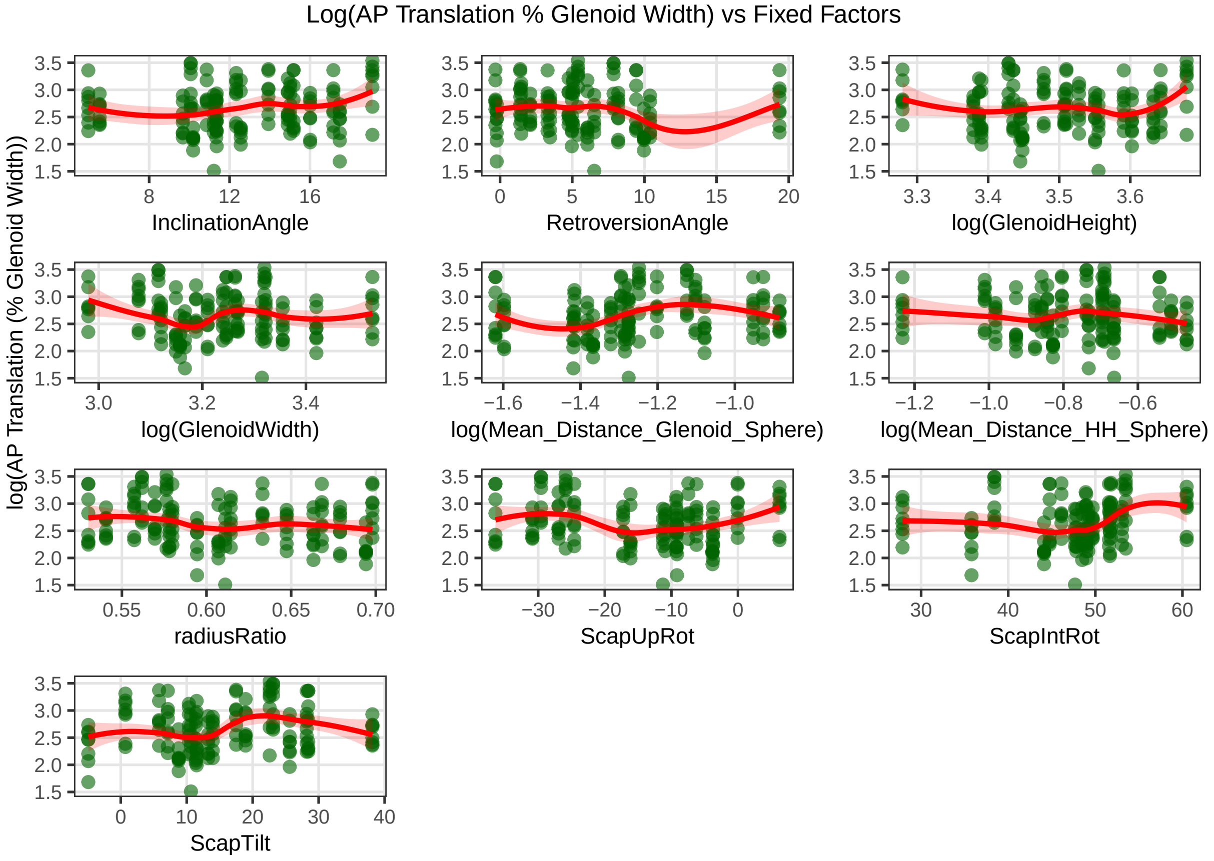

VIF Analysis for AP Model

```
# Check for multicollinearity among predictors for X translation (AP direction) model
# For X translation (AP direction), use glenoid width instead of height as size control
lmer_for_vif_X = lmer(log(X_translation) ~ InclinationAngle + RetroversionAngle +
↪  base_motion + weight + log(Mean_Distance_Glenoid_Sphere) +
↪  log(Mean_Distance_HH_Sphere) +  log(GlenoidHeight) + log(GlenoidWidth) + radiusRatio
↪  + ScapUpRot + ScapIntRot + ScapTilt + (1 | Subject), na.action = na.exclude, data =
↪  analysis_data_long_weight_factor)

# Calculate VIF values
vif_values_X <- vif(lmer_for_vif_X)
print("VIF values for X translation (AP direction) model:")
```

```
[1] "VIF values for X translation (AP direction) model:"
```

```
print(vif_values_X)
```

```
                                      GVIF Df GVIF^(1/(2*Df))
```

```
InclinationAngle                  3.765944  1        1.940604
RetroversionAngle                 3.231706  1        1.797695
base_motion                       1.252771  4        1.028570
weight                            1.251582  1        1.118741
log(Mean_Distance_Glenoid_Sphere) 2.001607  1        1.414782
log(Mean_Distance_HH_Sphere)      1.837026  1        1.355369
log(GlenoidHeight)                5.099782  1        2.258270
log(GlenoidWidth)                 6.844841  1        2.616265
radiusRatio                       2.035769  1        1.426804
ScapUpRot                         6.380176  1        2.525901
ScapIntRot                        1.828555  1        1.352241
ScapTilt                          5.481727  1        2.341309
```

We already know from the 3D analysis that width and height are correlated, and scap up rot and tilt are correlated. VIF analysis again shows that VIF values above 5 - since AP makes more sense to interpret relative to width, we remove height instead of width in this case We also remove scap up rot again because of its correlation with tilt.

```r
# rerun vif
lmer_for_vif_X = lmer(log(X_translation) ~ InclinationAngle + RetroversionAngle +
↪  base_motion + weight + log(Mean_Distance_Glenoid_Sphere) +
↪  log(Mean_Distance_HH_Sphere) + log(GlenoidWidth) + radiusRatio + ScapIntRot +
↪  ScapTilt + (1 | Subject), na.action = na.exclude, data =
↪  analysis_data_long_weight_factor)

# Calculate VIF values
vif_values_X <- vif(lmer_for_vif_X)
print("New VIF values for X translation (AP direction) model:")
```

```
[1] "New VIF values for X translation (AP direction) model:"
```

```r
print(vif_values_X)
```

```
                                      GVIF Df GVIF^(1/(2*Df))
InclinationAngle                  1.857766  1        1.362999
RetroversionAngle                 1.619633  1        1.272648
base_motion                       1.252596  4        1.028552
weight                            1.251552  1        1.118728
log(Mean_Distance_Glenoid_Sphere) 1.830490  1        1.352956
log(Mean_Distance_HH_Sphere)      1.778876  1        1.333745
log(GlenoidWidth)                 2.425408  1        1.557372
radiusRatio                       1.377025  1        1.173467
ScapIntRot                        1.463365  1        1.209696
ScapTilt                          1.918993  1        1.385277
```

Great, our VIF values are all below 5 now.

Fit LMER Model for AP Translations (removing height and scap up rot due to correlations between predictors)

```r
# For X translation (AP direction), use glenoid width instead of height as size control
lmer_main_X = lmer(log(X_translation) ~ InclinationAngle + RetroversionAngle +
↪   base_motion + weight + log(Mean_Distance_Glenoid_Sphere) +
↪   log(Mean_Distance_HH_Sphere) + log(GlenoidWidth) + radiusRatio + ScapIntRot +
↪   ScapTilt + (1 | Subject), na.action = na.exclude, data =
↪   analysis_data_long_weight_factor)

# Save residuals for AP model
analysis_data_long_weight_factor$residuals_AP <- resid(lmer_main_X)
```

Model Assumption Checks for AP Model

Now let's examine the model assumptions for the AP model using the same diagnostic plots we used for the 3D model.

QQ Plot for AP Model

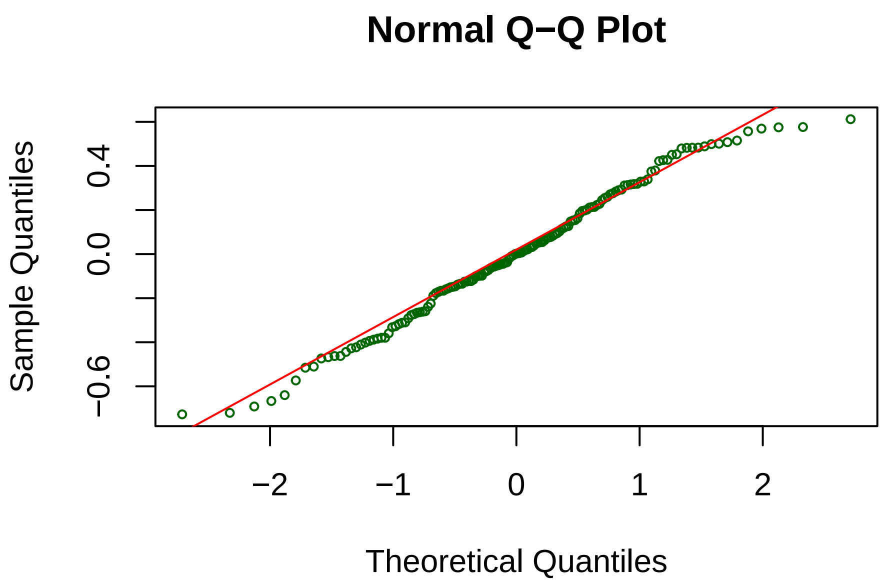

Interpretation Q-Q Plot for AP Model: *The residuals for the AP model appear to be normally distributed, similar to the 3D model.*

Tukey-Anscombe Plot for AP Model

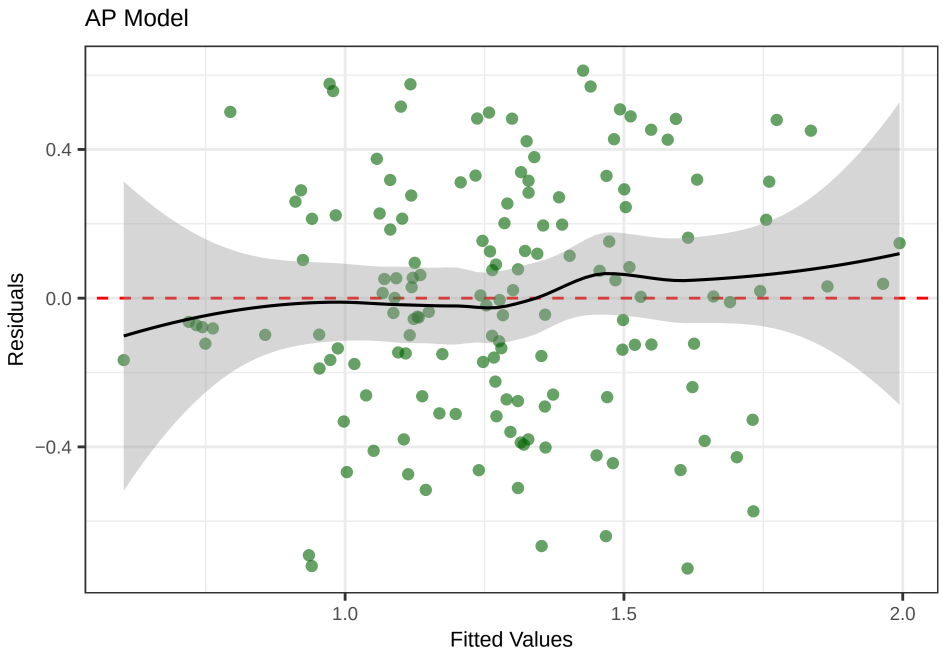

Interpretation of Tukey-Anscombe Plot for AP Model: The smoother here appears centered on 0 across fitted values, supporting the linear model (if the linear model was not an adequate fit, the residuals would change across fitted values). The decrease and increase in the smoother at the lowest and highest fitted values in the plot are likely due to the scarcity of the data in these regions (only 1-2 observations, which affect the smoother - the uncertainty of the smoother in these regions also increases due to low number of observations). The data also looks homoscedastic (equal variance of residuals across fitted values) in this plot.

Scale-Location Plot for AP Model

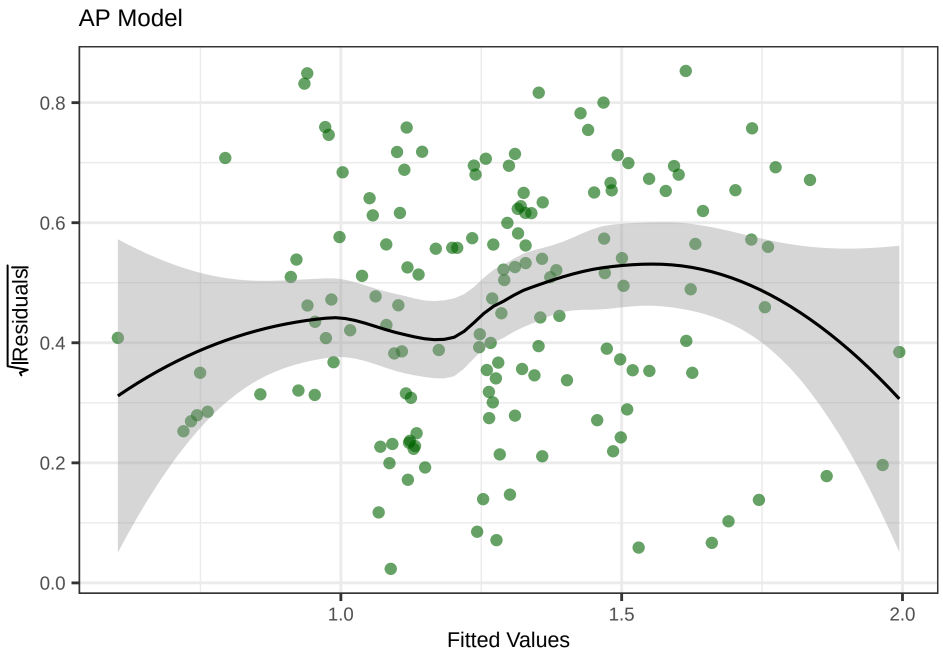

Interpretation of Scale-Location Plot for AP Model: *The variance of the residuals seems to be fairly constant, confirming homoscedasticity (equal variance of residuals, i.e. the fitted values do not appear to affect the spread of the residuals). Again, the changes at the ends of the plots are likely due to the low sample size in these regions/natural randomness of the data (the uncertainty of the smoothers in these regions also increases due to low number of observations).*

Residuals vs Predictors for AP Model

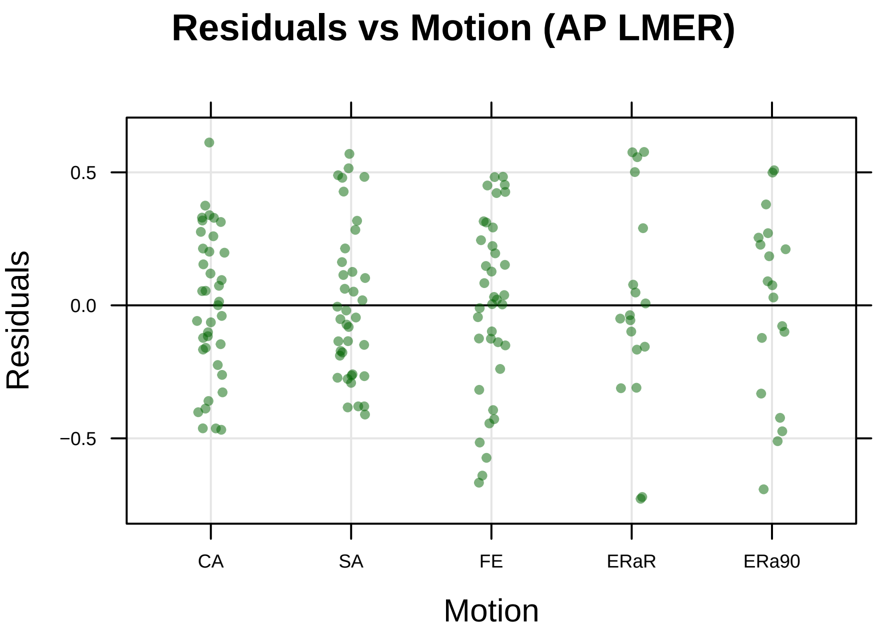

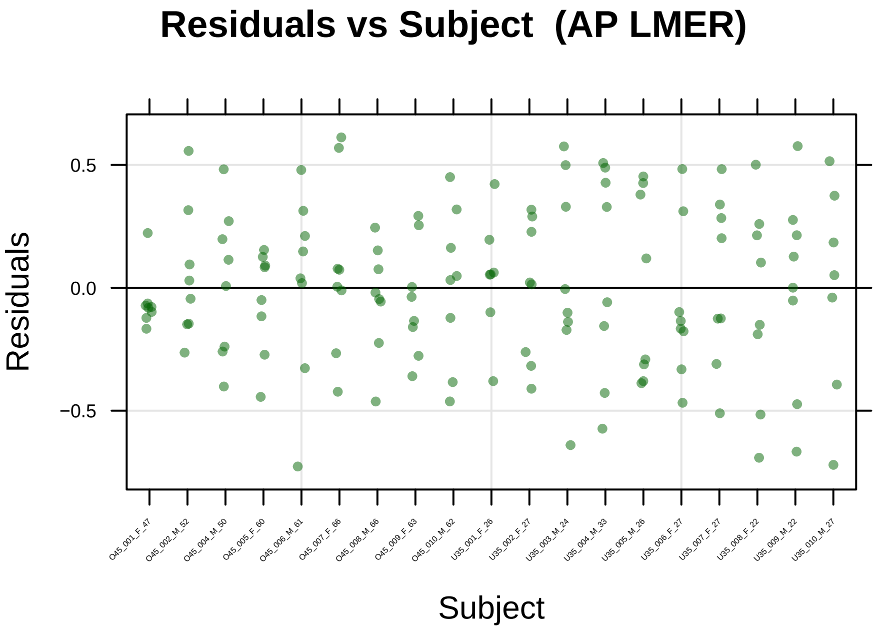

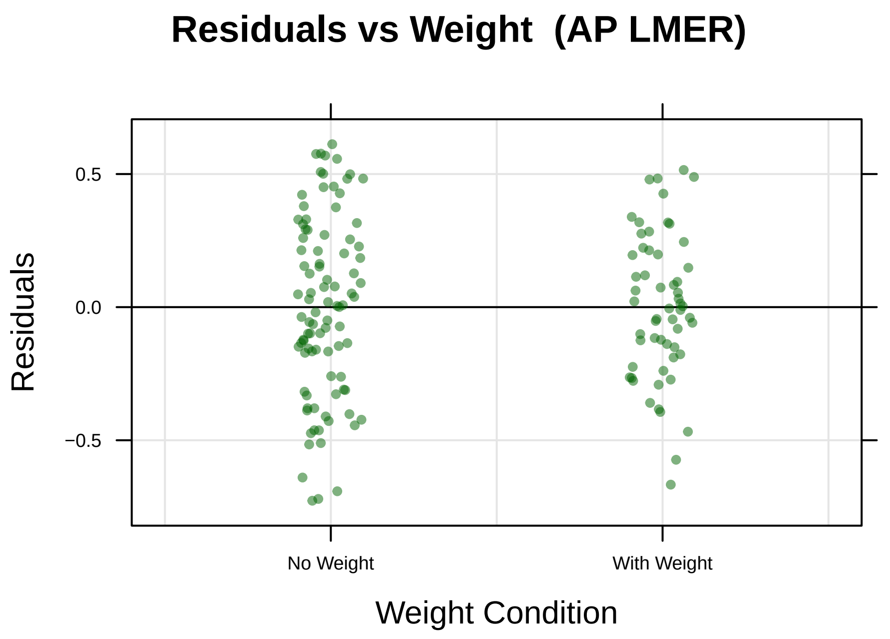
Residuals vs Weight (AP LMER)
0.5
0.0
−0.5
Residuals
No Weight
With Weight
Weight Condition

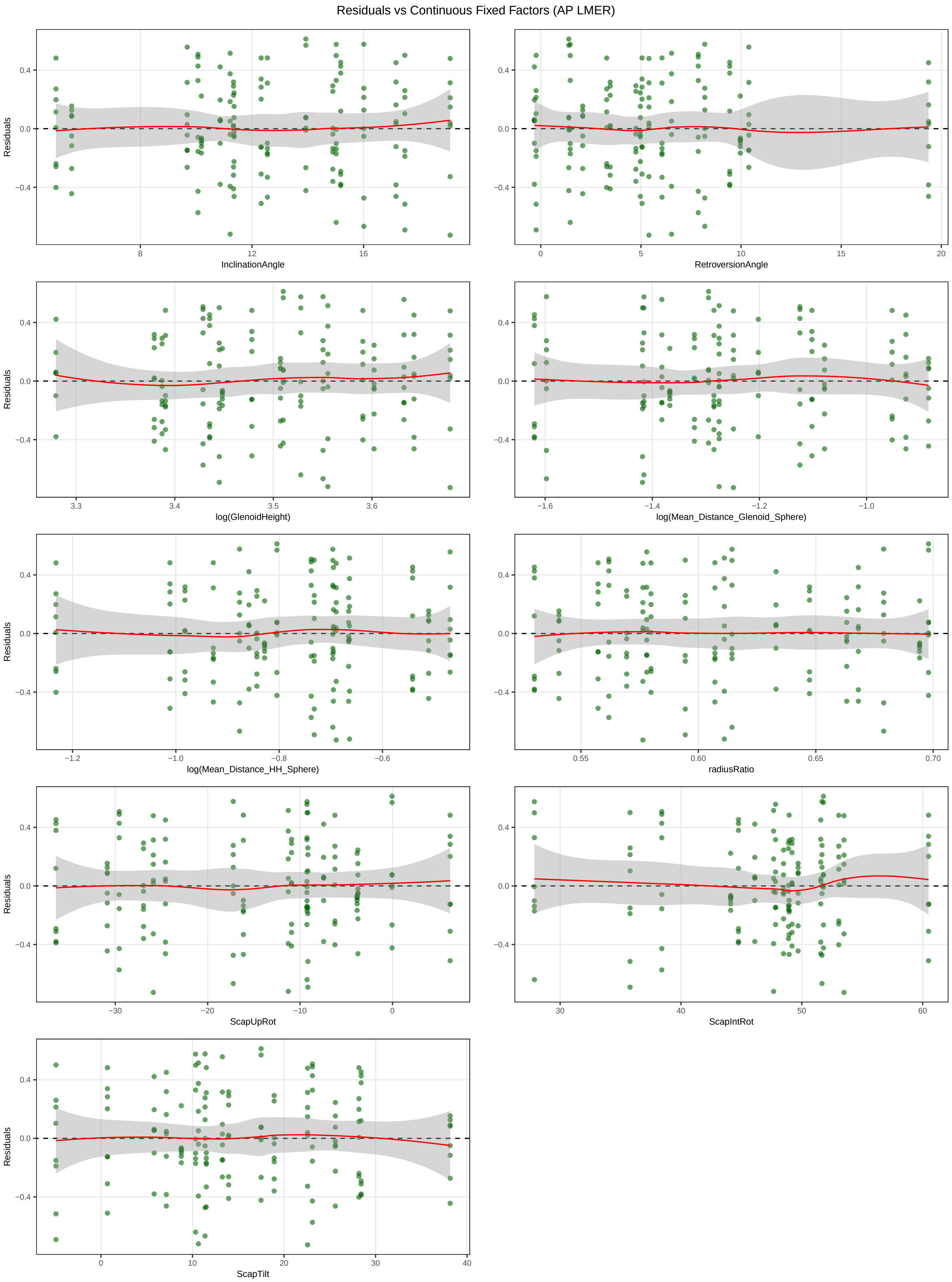

Interpretation of Residual Plots for AP Model: *These plots show that there is no clear correlation*

between the predictor values and the residuals (in other words, the smoothers are centered at 0), which supports our linearity assumption. The variance also does not appear to change based on predictor (meeting the homoscedasticity assumption).

Model Results for AP Model

```r
summary(lmer_main_X)
```

```
Linear mixed model fit by REML ['lmerMod']
Formula:
log(X_translation) ~ InclinationAngle + RetroversionAngle + base_motion +
    weight + log(Mean_Distance_Glenoid_Sphere) + log(Mean_Distance_HH_Sphere) +
    log(GlenoidWidth) + radiusRatio + ScapIntRot + ScapTilt +
    (1 | Subject)
   Data: analysis_data_long_weight_factor

REML criterion at convergence: 158.3

Scaled residuals:
     Min       1Q   Median       3Q      Max
-2.15844 -0.55247  0.00595  0.67248  1.81661

Random effects:
 Groups   Name        Variance Std.Dev.
 Subject  (Intercept) 0.03845  0.1961
 Residual             0.11351  0.3369
Number of obs: 150, groups:  Subject, 19

Fixed effects:
                                   Estimate Std. Error t value
(Intercept)                        1.137403   2.199140   0.517
InclinationAngle                   0.051493   0.019587   2.629
RetroversionAngle                  0.003376   0.014889   0.227
base_motionSA                      0.013498   0.077294   0.175
base_motionFE                      0.233786   0.077874   3.002
base_motionERaR                   -0.116730   0.101679  -1.148
base_motionERa90                   0.024208   0.099836   0.242
weightTRUE                         0.029690   0.063427   0.468
log(Mean_Distance_Glenoid_Sphere)  0.657466   0.358349   1.835
log(Mean_Distance_HH_Sphere)      -0.176103   0.388149  -0.454
log(GlenoidWidth)                  0.114470   0.659080   0.174
radiusRatio                       -0.862255   1.216887  -0.709
ScapIntRot                         0.001361   0.009119   0.149
ScapTilt                           0.012526   0.007112   1.761
```

```r
# drop1 analysis AP translations
drop1(lmer_main_X, test = "Chisq")
```

```
Single term deletions

Model:
```

```
log(X_translation) ~ InclinationAngle + RetroversionAngle + base_motion +
    weight + log(Mean_Distance_Glenoid_Sphere) + log(Mean_Distance_HH_Sphere) +
    log(GlenoidWidth) + radiusRatio + ScapIntRot + ScapTilt +
    (1 | Subject)
                                 npar    AIC     LRT  Pr(Chi)
<none>                                138.73
InclinationAngle                    1 146.71  9.9780 0.001584 **
RetroversionAngle                   1 136.83  0.0989 0.753156
base_motion                         4 146.44 15.7141 0.003428 **
weight                              1 136.96  0.2268 0.633910
log(Mean_Distance_Glenoid_Sphere)   1 142.23  5.4951 0.019070 *
log(Mean_Distance_HH_Sphere)        1 137.13  0.3942 0.530107
log(GlenoidWidth)                   1 136.79  0.0626 0.802377
radiusRatio                         1 137.67  0.9409 0.332037
ScapIntRot                          1 136.77  0.0417 0.838226
ScapTilt                            1 141.86  5.1298 0.023518 *
---
Signif. codes:  0 '***' 0.001 '**' 0.01 '*' 0.05 '.' 0.1 ' ' 1
```

Effect of Inclination Angle on AP Translations

Coefficient: $\hat{\beta} = 0.0515$ (95% CI 0.0221 to 0.0808) for `InclinationAngle`.

Raw x, $\log y$ — % per +1 unit x.

Model: $\log y = \cdots + \beta x + \cdots$ (other terms held fixed).

Formula: $100\,(e^{\beta} - 1)$ with $\hat{\beta} = 0.0515$.

Interpretation: 5.3% (95% CI 2.2% to 8.4%) change in AP translation per degree change in *inclination angle.*

Raw x, $\log y$ — raw Δx for +1 mm y at $\bar{y}$.

Model: $\log y = \cdots + \beta x + \cdots$ (other terms held fixed).

Reference (sample means): $\bar{y} = 3.9$ mm (mean translation).

Formula: $F_y = (\bar{y} + 1)/\bar{y}$, $\Delta x = \log(F_y)/\beta$; $F_y = 1.25364$, $\log(F_y) = 0.22605$, $\Delta x \approx 4.4\,\text{deg}$.

Interpretation: Approximately 4.4 deg change in *inclination angle* corresponds to a +1 mm increase in AP translation from $\bar{y} = 3.9$ mm.

Effect of log(Mean_Distance_Glenoid_Sphere) on AP Translations

Coefficient: $\hat{\beta} = 0.6575$ (95% CI 0.1196 to 1.1931) for `log(Mean_Distance_Glenoid_Sphere)`.

$\log x$, $\log y$ — % per +1% x.

Model: $\log y = \cdots + \beta \log x + \cdots$ (other terms held fixed).

Formula: $100\,(1.01^{\beta} - 1)$ with $\hat{\beta} = 0.6575$; $1.01^{\hat{\beta}} \approx 1.0066$ (95% CI 1.0012 to 1.0119).

Interpretation: 0.66% (95% CI 0.12% to 1.19%) change in AP translation per 1% increase in *glenoid asphericity (mean distance to fitted sphere, mm).*

$\log x$, $\log y$ — raw Δx for +1 mm y at $\bar{y}$.

Model: $\log y = \cdots + \beta \log x + \cdots$ (other terms held fixed).

Reference (sample means): $\bar{y} = 3.9$ mm; $\hat{x} = 0.3\,\text{mm}$ (mean raw predictor).

Formula: $F_y = (\bar{y}+1)/\bar{y}$, $\Delta x = \hat{x}\left(F_y^{1/\beta} - 1\right)$; $F_y = 1.25364$, $F_y^{1/\hat{\beta}} \approx 1.41033$, $\Delta x \approx 0.1\,\text{mm}$.

Interpretation: Approximately 0.1 mm change in *glenoid asphericity (mean distance to fitted sphere, mm)* from $\hat{x}$ corresponds to a +1 mm increase in AP translation from $\bar{y} = 3.9$ mm.

Effect of Scapular Tilt on AP Translations

Coefficient: $\hat{\beta} = 0.0125$ (95% CI 0.0019 to 0.0232) for `ScapTilt`.

Raw x, $\log y$ — % per +1 unit x.

Model: $\log y = \cdots + \beta x + \cdots$ (other terms held fixed).

Formula: $100\,(e^{\beta} - 1)$ with $\hat{\beta} = 0.0125$.

Interpretation: 1.3% (95% CI 0.2% to 2.3%) change in AP translation per degree change in *scapular tilt*.

Raw x, $\log y$ — raw Δx for +1 mm y at $\bar{y}$.

Model: $\log y = \cdots + \beta x + \cdots$ (other terms held fixed).

Reference (sample means): $\bar{y} = 3.9$ mm (mean translation).

Formula: $F_y = (\bar{y}+1)/\bar{y}$, $\Delta x = \log(F_y)/\beta$; $F_y = 1.25364$, $\log(F_y) = 0.22605$, $\Delta x \approx 18.0\,\text{deg}$.

Interpretation: Approximately 18.0 deg change in *scapular tilt* corresponds to a +1 mm increase in AP translation from $\bar{y} = 3.9$ mm.

Post Hoc Comparisons for AP Model

```r
# Perform post-hoc analysis for AP model
posthoc_results_AP <- perform_posthoc_analysis(lmer_main_X, "X Translation (AP
↪  Direction)")
```

```
     Simultaneous Tests for General Linear Hypotheses

Multiple Comparisons of Means: Tukey Contrasts


Fit: lmer(formula = log(X_translation) ~ InclinationAngle + RetroversionAngle +
    base_motion + weight + log(Mean_Distance_Glenoid_Sphere) +
    log(Mean_Distance_HH_Sphere) + log(GlenoidWidth) + radiusRatio +
    ScapIntRot + ScapTilt + (1 | Subject), data = analysis_data_long_weight_factor,
    na.action = na.exclude)

Linear Hypotheses:
                  Estimate Std. Error z value Pr(>|z|)
SA - CA == 0       0.01350    0.07729   0.175  0.99979
FE - CA == 0       0.23379    0.07787   3.002  0.02189 *
ERaR - CA == 0    -0.11673    0.10168  -1.148  0.77703
ERa90 - CA == 0    0.02421    0.09984   0.242  0.99922
FE - SA == 0       0.22029    0.07787   2.829  0.03667 *
ERaR - SA == 0    -0.13023    0.10168  -1.281  0.69850
ERa90 - SA == 0    0.01071    0.09984   0.107  0.99997
ERaR - FE == 0    -0.35052    0.10243  -3.422  0.00549 **
```

```
ERa90 - FE == 0   -0.20958    0.10059  -2.084  0.22278
ERa90 - ERaR == 0  0.14094    0.11099   1.270  0.70527
---
Signif. codes:  0 '***' 0.001 '**' 0.01 '*' 0.05 '.' 0.1 ' ' 1
(Adjusted p values reported -- single-step method)

Significant motion differences:
FE - CA p = 0.022
FE - SA p = 0.037
ERaR - FE p = 0.005
FE has 26.3% higher translations than CA (95% CI: 2.2% to 56.1%, p=0.022)

FE has 24.6% higher translations than SA (95% CI: 0.9% to 54.0%, p=0.037)

ERaR has 29.6% lower translations than FE (95% CI: 7.0% to 46.7%, p=0.005)
```

Interpretation Motion Effects: *The post-hoc analysis shows significant pairwise differences between some motions. This is also shown graphically in Appendix A (Supplementary Plots) in the publication.*

Starting Point Assessment

For this analysis, we check the Euclidean distance (in XY, i.e. in the glenoid plane) of the starting point to the center of the inferior glenoid best fit circle (i.e. the origin of the glenoid coordinate system). We then check how many subjects and trials have the starting point closer to the geometric mean versus to the origin of the glenoid coordinate system. Then, we check the AP positioning of the starting point by testing whether the X value (in the glenoid coordinate system) is positive (anterior) or negative (posterior). All analyses are conducted by pooling all subjects and trials, as well as on a per motion basis.

Table: Starting Point to Reference Points - Distances as % Glenoid Height

Motion	Dist to Origin (%)	Dist to GeoMean (%)	Closer to GeoMean
All	9.6%+/-2.9 (1.6-16.6)	4.8%+/-3.1 (0.3-20.5)	134/150
CA	7.4%+/-3.5 (1.6-13.0)	5.3%+/-3.0 (1.2-12.2)	13/19
ERa90	10.2%+/-1.9 (7.3-14.6)	3.9%+/-2.8 (0.3-12.2)	19/19
ERaR	8.9%+/-2.8 (3.9-13.7)	6.6%+/-4.6 (0.9-20.5)	13/18
FE	10.8%+/-2.1 (7.3-16.6)	4.6%+/-3.2 (1.0-9.6)	17/18
SA	9.0%+/-2.5 (4.6-12.7)	4.7%+/-2.7 (0.6-10.0)	17/19
WCA	9.1%+/-3.3 (3.5-16.6)	4.4%+/-2.7 (0.8-10.1)	18/19
WFE	10.6%+/-2.8 (5.3-15.7)	4.4%+/-2.5 (0.6-10.7)	19/19
WSA	10.6%+/-2.9 (5.8-16.2)	4.8%+/-3.0 (0.6-11.0)	18/19

Table: Starting Point to Reference Points - Distances in mm

Motion	Dist to Origin (mm)	Dist to GeoMean (mm)	Closer to GeoMean
All	3.2+/-1.0 (0.6-6.0)	1.6+/-1.1 (0.1-7.0)	134/150
CA	2.4+/-1.2 (0.6-4.3)	1.8+/-1.1 (0.4-4.1)	13/19
ERa90	3.4+/-0.6 (2.3-4.6)	1.3+/-0.9 (0.1-3.6)	19/19

ERaR	3.0+/-1.0 (1.3-5.4)	2.2+/-1.6 (0.3-7.0)	13/18	
FE	3.6+/-0.8 (2.5-6.0)	1.6+/-1.2 (0.3-3.5)	17/18	
SA	3.0+/-0.9 (1.6-4.5)	1.6+/-1.0 (0.2-3.2)	17/19	
WCA	3.0+/-1.0 (1.2-5.1)	1.5+/-1.0 (0.3-3.4)	18/19	
WFE	3.5+/-1.0 (1.6-5.2)	1.5+/-1.0 (0.2-4.2)	19/19	
WSA	3.5+/-0.9 (1.9-5.4)	1.6+/-1.1 (0.2-3.7)	18/19	

Starting Point - Violin and Paired Plots

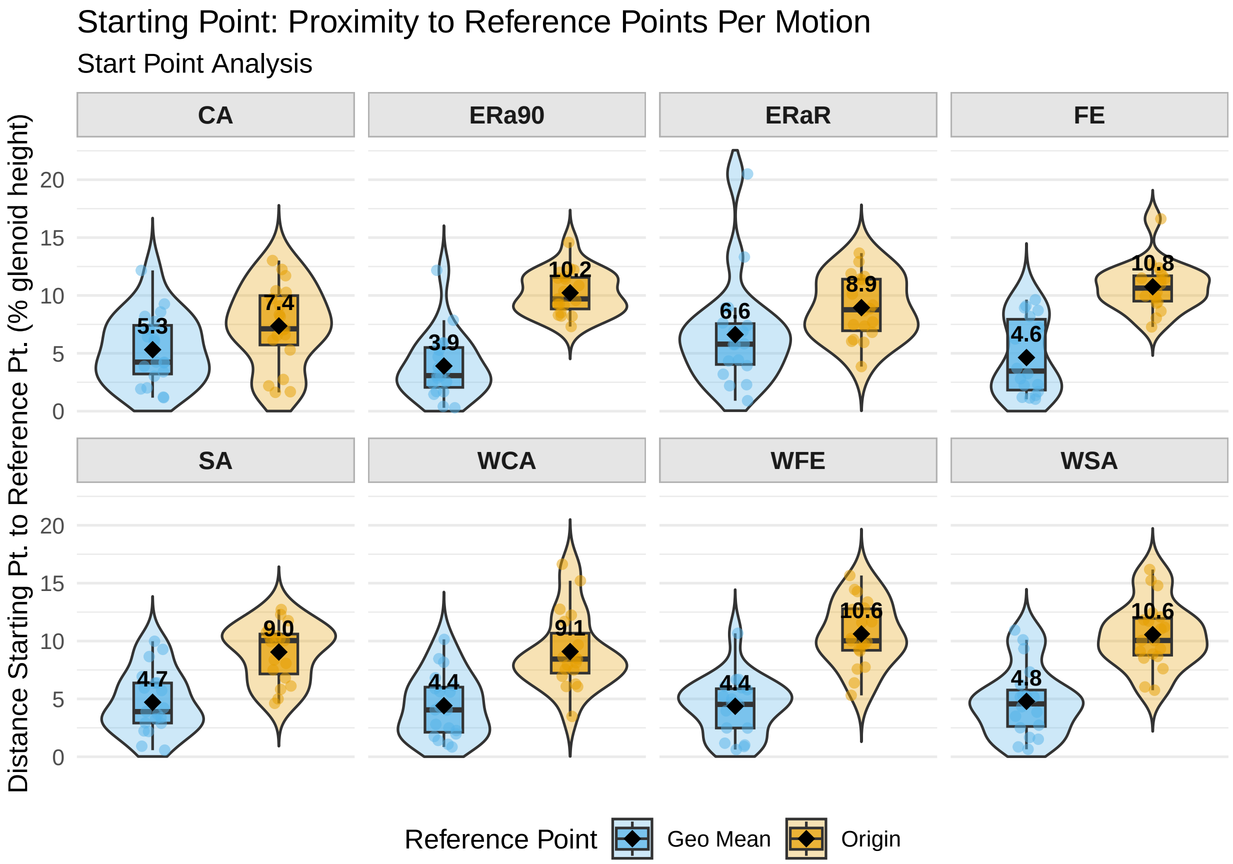

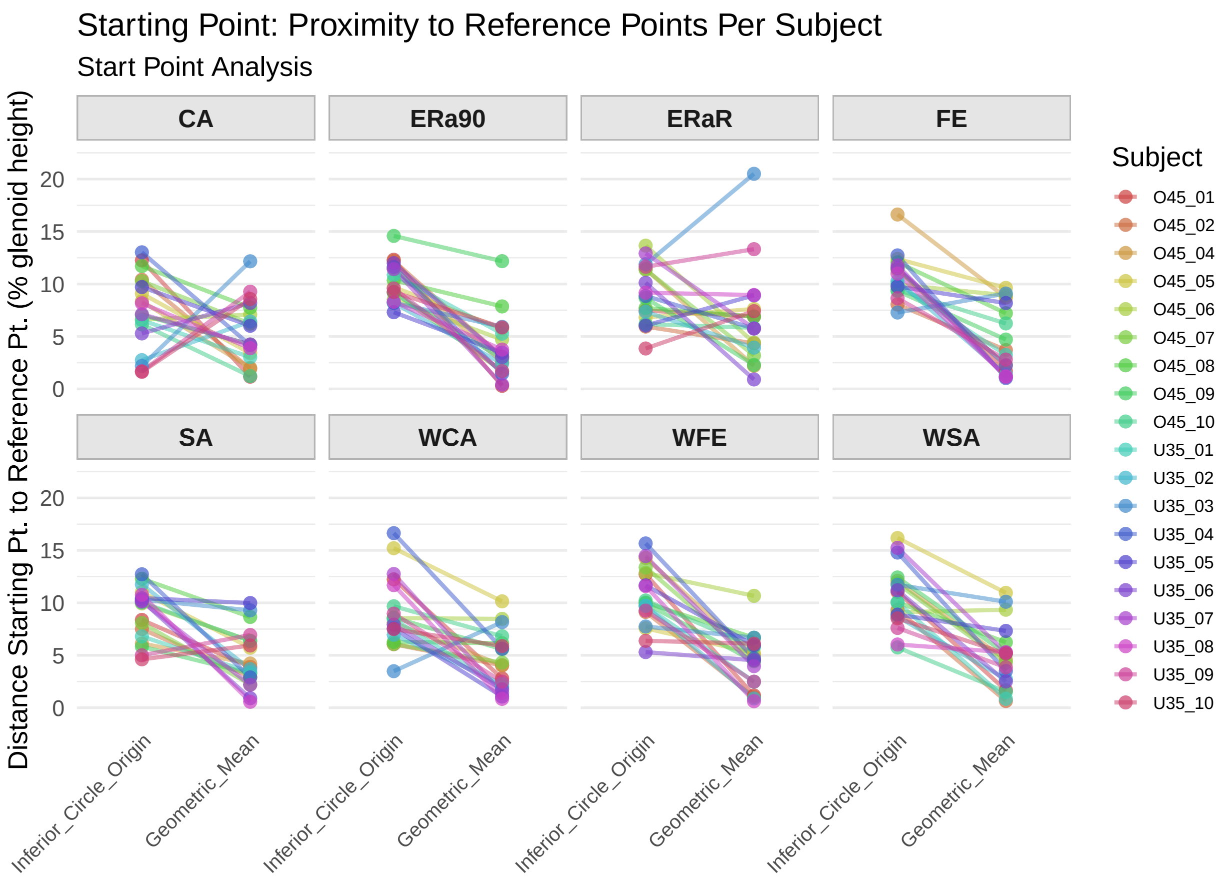

Table: X-Axis Starting Point Analysis - Distances as % Glenoid Width

Motion	X Distance to Origin (%)	Positive X	Negative X
All	3.0%+/-4.8 (-14.9-15.6)	108	42
CA	1.6%+/-4.1 (-6.0-9.2)	13	6
ERa90	0.9%+/-5.6 (-14.9-9.4)	12	7
ERaR	5.4%+/-4.7 (-2.9-15.6)	15	3
FE	3.8%+/-5.1 (-4.3-11.7)	13	5
SA	3.6%+/-4.3 (-3.3-11.4)	15	4
WCA	2.2%+/-4.4 (-6.6-11.1)	14	5
WFE	2.5%+/-5.1 (-4.6-14.0)	11	8
WSA	4.0%+/-4.8 (-4.8-12.6)	15	4

Table: X-Axis Starting Point Analysis - Distances in mm

Motion	X Distance to Origin (mm)	Positive X	Negative X
All	0.8+/-1.3 (-3.6-3.9)	108	42
CA	0.5+/-1.1 (-1.4-2.8)	13	6
ERa90	0.3+/-1.4 (-3.6-2.9)	12	7

ERaR	1.4+/-1.2 (-0.7-3.9)	15	3
FE	1.1+/-1.4 (-1.0-3.1)	13	5
SA	1.0+/-1.2 (-0.8-3.1)	15	4
WCA	0.6+/-1.2 (-1.6-2.9)	14	5
WFE	0.7+/-1.4 (-1.3-3.9)	11	8
WSA	1.1+/-1.3 (-1.2-3.2)	15	4

Start-End Point Pairwise Difference (StartEndDiff)

This section computes the pairwise difference (StartEndDiff) between starting and ending points in the glenoid XY plane, and repeats the starting-point-style analysis for endpoint positions.

```
**StartEndDiff (pairwise XY distance start to end) summary:**

  In mm: Mean = 1.11, SD = 0.93, Range = [0.08, 6.39]

  Scaled to glenoid height (%): Mean = 3.33%, SD = 2.86%, Range = [0.25%, 20.73%]

  n = 150 subject-motion pairs
```

Table: StartEndDiff per motion - pairwise XY distance from start to end

Motion	n	Mean (mm)	SD (mm)	Min (mm)	Max (mm)	Mean (% GH)	SD (% GH)	Min (% GH)	Max (% GH)
All	150	1.11	0.93	0.08	6.39	3.33	2.86	0.25	20.73
CA	19	0.89	0.52	0.08	2.30	2.71	1.61	0.25	6.59
ERa90	19	1.59	1.45	0.16	6.39	4.76	4.51	0.61	20.73
ERaR	18	1.11	0.71	0.10	2.61	3.32	2.11	0.32	6.98
FE	18	1.29	1.46	0.13	6.32	3.87	4.61	0.38	20.35
SA	19	0.92	0.67	0.24	2.43	2.79	2.11	0.76	7.83
WCA	19	1.02	0.59	0.20	2.34	3.02	1.69	0.56	7.52
WFE	19	1.18	0.93	0.12	3.10	3.49	2.69	0.38	7.82
WSA	19	0.90	0.51	0.14	2.03	2.72	1.50	0.45	5.60

Endpoint Analysis**

We do the same analysis as we did for start points but using the end point (which should be similar to the start point since they are both at rest) to check the sensivity of our analyses.

Table: Endpoint to Reference Points - Distances as % Glenoid Height

Motion	Dist to Origin (%)	Dist to GeoMean (%)	Closer to GeoMean
All	10.2%+/-3.3 (2.6-29.7)	5.0%+/-3.5 (0.5-24.2)	133/150
CA	8.6%+/-2.7 (3.3-13.7)	4.8%+/-2.9 (1.4-13.3)	16/19
ERa90	10.8%+/-4.0 (4.0-21.8)	5.1%+/-4.3 (0.6-19.4)	18/19

ERaR	8.4%+/-3.2 (2.6-13.3)	7.4%+/-3.9 (0.5-14.1)	10/18
FE	12.0%+/-5.0 (6.2-29.7)	5.2%+/-5.4 (1.0-24.2)	17/18
SA	10.3%+/-2.2 (5.5-13.8)	4.3%+/-2.4 (1.3-11.7)	18/19
WCA	9.1%+/-2.5 (4.8-14.4)	4.7%+/-2.6 (1.3-10.8)	17/19
WFE	10.8%+/-2.5 (6.1-15.3)	4.0%+/-2.4 (0.7-8.6)	18/19
WSA	11.2%+/-2.4 (7.0-14.8)	4.6%+/-2.8 (1.5-11.1)	19/19

Table: Endpoint to Reference Points - Distances in mm

Motion	Dist to Origin (mm)	Dist to GeoMean (mm)	Closer to GeoMean
All	3.4+/-1.1 (1.0-9.2)	1.7+/-1.2 (0.2-7.5)	133/150
CA	2.9+/-1.0 (1.1-4.2)	1.6+/-1.0 (0.4-4.5)	16/19
ERa90	3.6+/-1.3 (1.5-6.7)	1.7+/-1.3 (0.2-6.0)	18/19
ERaR	2.8+/-1.1 (1.0-5.3)	2.5+/-1.3 (0.2-4.8)	10/18
FE	4.0+/-1.6 (2.2-9.2)	1.7+/-1.8 (0.3-7.5)	17/18
SA	3.4+/-0.7 (1.5-4.4)	1.4+/-0.9 (0.4-4.0)	18/19
WCA	3.0+/-0.9 (1.7-4.4)	1.6+/-0.9 (0.4-3.7)	17/19
WFE	3.6+/-0.8 (2.3-4.9)	1.3+/-0.8 (0.2-2.9)	18/19
WSA	3.7+/-0.8 (2.5-4.9)	1.5+/-1.0 (0.6-3.8)	19/19

Table: X-Axis Endpoint Analysis - Distances as % Glenoid Width

Motion	X Distance to Origin (%)	Positive X	Negative X
All	2.7%+/-5.7 (-26.0-23.1)	110	40
CA	2.9%+/-3.8 (-4.8-9.5)	14	5
ERa90	-4.6%+/-6.9 (-26.0-3.4)	4	15
ERaR	6.4%+/-3.9 (-0.6-11.8)	17	1
FE	4.1%+/-6.2 (-2.1-23.1)	13	5
SA	3.0%+/-4.3 (-4.4-13.3)	16	3
WCA	3.4%+/-4.2 (-5.1-9.5)	16	3
WFE	2.6%+/-4.3 (-7.4-10.1)	14	5
WSA	4.0%+/-4.9 (-6.5-13.6)	16	3

Table: X-Axis Endpoint Analysis - Distances in mm

Motion	X Distance to Origin (mm)	Positive X	Negative X
All	0.7+/-1.4 (-5.9-5.9)	110	40
CA	0.8+/-1.0 (-1.2-2.9)	14	5
ERa90	-1.1+/-1.6 (-5.9-1.0)	4	15
ERaR	1.6+/-0.9 (-0.1-2.9)	17	1
FE	1.1+/-1.7 (-0.4-5.9)	13	5
SA	0.8+/-1.2 (-1.3-3.4)	16	3
WCA	0.9+/-1.1 (-1.2-2.4)	16	3
WFE	0.7+/-1.1 (-1.7-2.6)	14	5
WSA	1.0+/-1.3 (-1.6-3.5)	16	3

Endpoint - Violin and Paired Plots

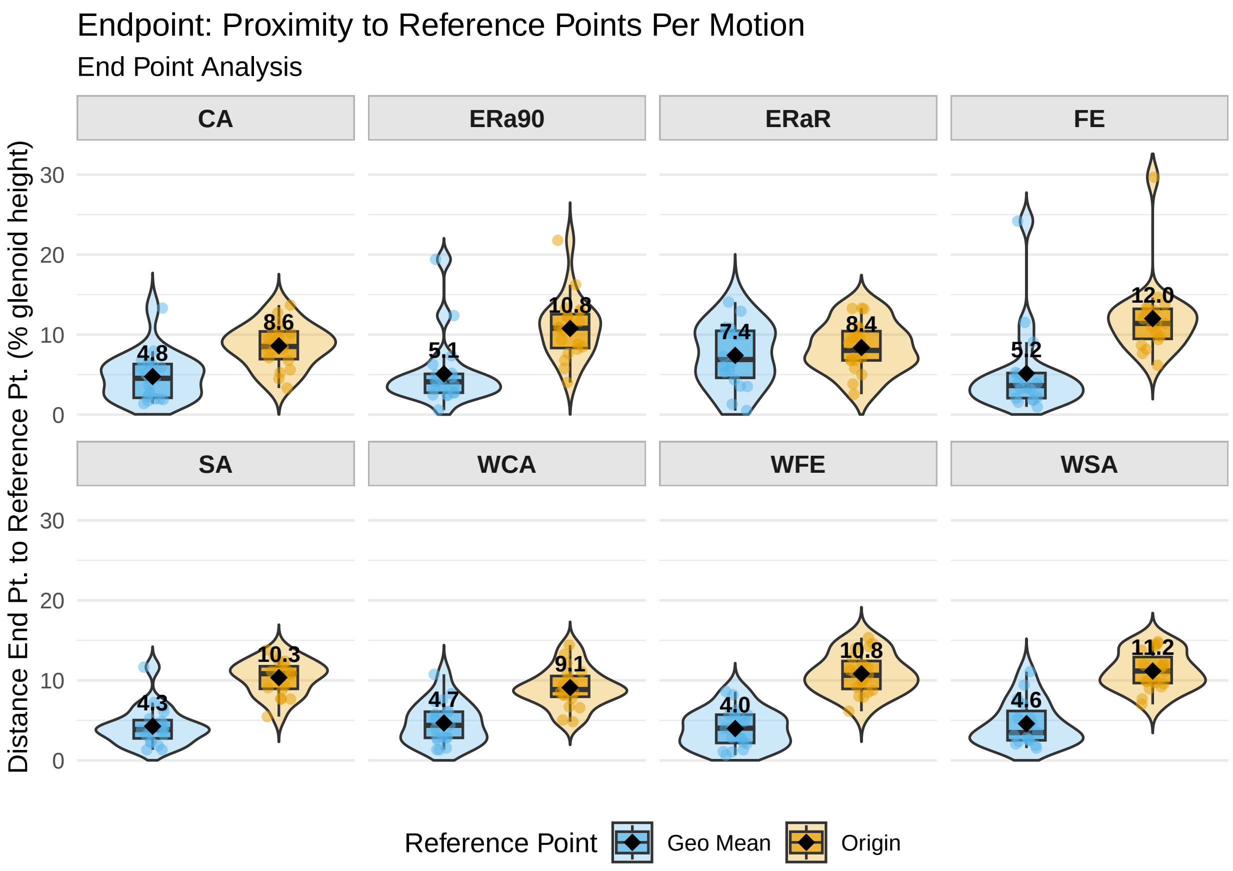

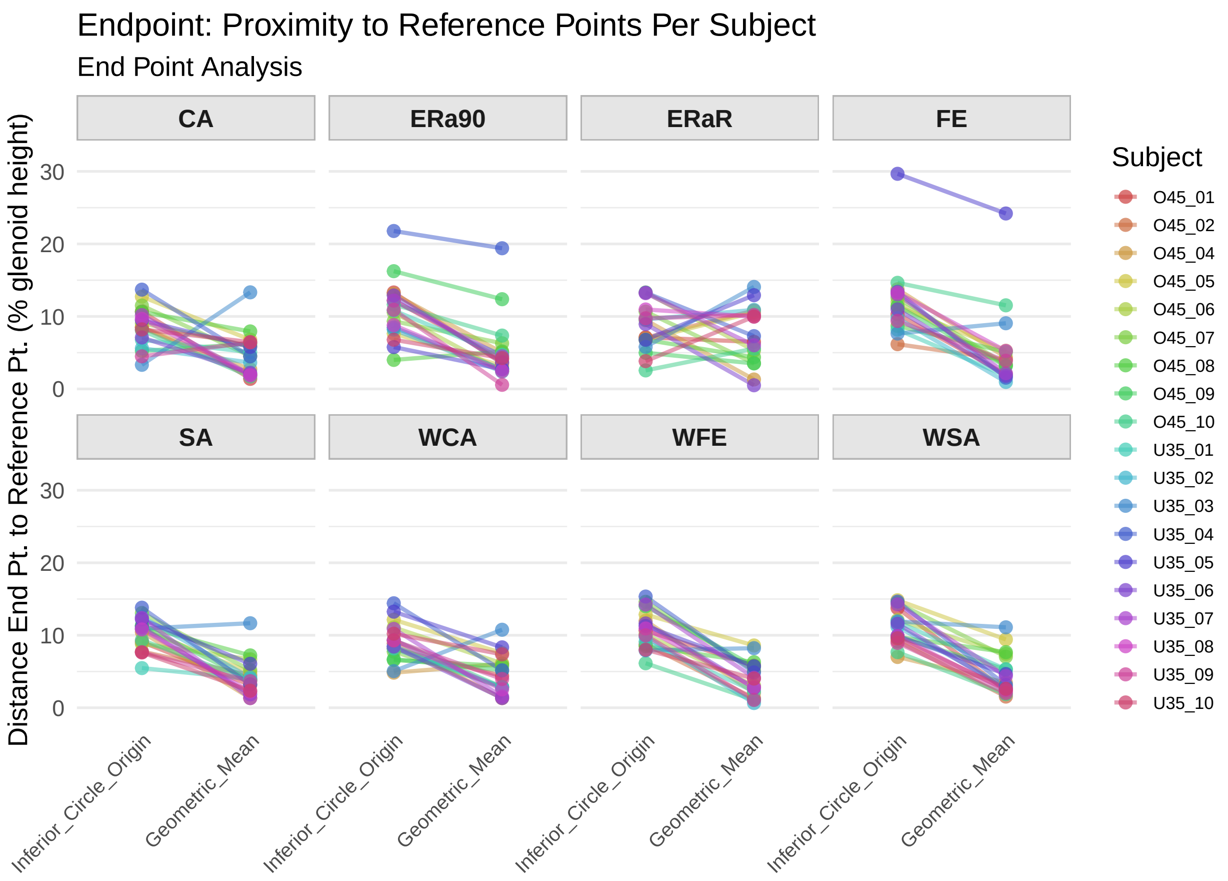


```
Table: Summary of Significant Post-hoc Motion Effects

|              |Model |Motion Pair     | % Change|95% CI                | CI Range (%)|p-value |
|:-------------|:-----|:---------------|--------:|:---------------------|------------:|:-------|
|ERa90 - FE    |3D    |ERa90 $<$ FE    |    -26.8|[-42.5\%, -6.8\%]     |         35.6|0.004   |
|ERa90 - CA    |SI    |ERa90 $<$ CA    |    -50.5|[-62.4\%, -34.8\%]    |         27.7|<0.001  |
|ERa90 - SA    |SI    |ERa90 $<$ SA    |    -39.5|[-54.1\%, -20.2\%]    |         33.8|<0.001  |
|ERa90 - FE1   |SI    |ERa90 $<$ FE    |    -46.0|[-59.1\%, -28.7\%]    |         30.4|<0.001  |
|ERa90 - ERaR  |SI    |ERa90 $<$ ERaR  |    -48.2|[-61.9\%, -29.6\%]    |         32.3|<0.001  |
|FE - CA       |AP    |FE $>$ CA       |     26.3|[2.2\%, 56.1\%]       |         53.9|0.022   |
|FE - SA       |AP    |FE $>$ SA       |     24.6|[0.9\%, 54.0\%]       |         53.1|0.037   |
|ERaR - FE     |AP    |ERaR $<$ FE     |    -29.6|[-46.7\%, -7.0\%]     |         39.7|0.005   |
```
